# Interface synergism and engineering of Pd/Co@N-C for direct ethanol fuel cells

**DOI:** 10.1038/s41467-023-37011-z

**Published:** 2023-03-11

**Authors:** Jinfa Chang, Guanzhi Wang, Xiaoxia Chang, Zhenzhong Yang, Han Wang, Boyang Li, Wei Zhang, Libor Kovarik, Yingge Du, Nina Orlovskaya, Bingjun Xu, Guofeng Wang, Yang Yang

**Affiliations:** 1 NanoScience Technology Center, University of Central Florida, Orlando, FL 32826 USA; 2 Department of Materials Science and Engineering, University of Central Florida, Orlando, FL 32826 USA; 3 Catalysis Center for Energy Innovation, Department of Chemical and Biomolecular Engineering, University of Delaware, Newark, Delaware, 19716 USA; 4 Physical and Computational Sciences Directorate, Pacific Northwest National Laboratory, Richland, WA 99352 USA; 5 Department of Mechanical Engineering and Materials Science, University of Pittsburgh, Pittsburgh, PA 15261 USA; 6 Department of Mechanical and Aerospace Engineering, University of Central Florida, Orlando, FL 32816 USA; 7 Renewable Energy and Chemical Transformation Cluster, University of Central Florida, Orlando, FL 32816 USA; 8 Department of Chemistry, University of Central Florida, Orlando, FL 32816 USA; 9 The Stephen W. Hawking Center for Microgravity Research and Education, University of Central Florida, Orlando, FL 32826 USA

**Keywords:** Fuel cells, Fuel cells, Fuel cells

## Abstract

Direct ethanol fuel cells have been widely investigated as nontoxic and low-corrosive energy conversion devices with high energy and power densities. It is still challenging to develop high-activity and durable catalysts for a complete ethanol oxidation reaction on the anode and accelerated oxygen reduction reaction on the cathode. The materials’ physics and chemistry at the catalytic interface play a vital role in determining the overall performance of the catalysts. Herein, we propose a Pd/Co@N-C catalyst that can be used as a model system to study the synergism and engineering at the solid-solid interface. Particularly, the transformation of amorphous carbon to highly graphitic carbon promoted by cobalt nanoparticles helps achieve the spatial confinement effect, which prevents structural degradation of the catalysts. The strong catalyst-support and electronic effects at the interface between palladium and Co@N-C endow the electron-deficient state of palladium, which enhances the electron transfer and improved activity/durability. The Pd/Co@N-C delivers a maximum power density of 438 mW cm^−2^ in direct ethanol fuel cells and can be operated stably for more than 1000 hours. This work presents a strategy for the ingenious catalyst structural design that will promote the development of fuel cells and other sustainable energy-related technologies.

## Abstract (web summary)

It is challenging to develop high-activity and durable catalysts for both ethanol oxidation reaction on the anode and oxygen reduction reaction on the cathode. Here in this work, authors proposed Pd/Co@N-C catalyst as a model to synergistically maximize the usage of catalyst nanoparticles and active interfaces for direct ethanol fuel cells.

## Introduction

Fuel cells (FCs) are promising energy conversion devices for defense and civil applications due to their high energy/power densities^1,2^. Among all FCs, direct ethanol (EtOH) fuel cells (DEFCs) have attracted much attention because of their low weight, high energy density, mild operation conditions, and simple system design^3^. Platinum (Pt) group metals (PGMs)-based catalysts have been widely developed for DEFCs thanks to their optimal electronic structures towards catalytic reactions^4–6^. However, the widespread commercialization of DEFCs is severely challenged by the high cost of PGMs, sluggish reaction kinetics, incomplete EtOH oxidation reaction (EOR), and catalyst poisoning^7^. Although recent work suggests that the catalysts with low-PGMs loading and even PGMs-free catalysts can be used^8–10^ to reduce the reliance on PGMs, a substantially increased thickness of membrane electrode assembly (MEA) with high catalyst loading is required to achieve the comparable performance to the PGMs-based catalysts, which in turn causes mass transport issues in practical fuel cells^11^. Thus, both fundamental and applied research on catalysts with low cost, high activity, and long stability for DEFCs are in critical demand.

Palladium (Pd)-based catalysts exhibit a comparable electrocatalytic EOR activity to Pt in alkaline media, making alkaline DEFCs promising for scalable commercialization^12–14^. However, the catalytic activity and stability of Pd are still far from an ideal catalyst for alkaline DEFCs^15^. The reaction kinetics and pathways in the alkaline EOR are exceptionally complex because of the multi-electron transfer processes. The most invoked reaction pathways for EOR are C1-12e and C2-4e pathways (see Supplementary Note 1)^13^. Ideally, a complete C1-12e pathway is the most desirable process for the complete oxidation of EtOH to CO_2_, thus, delivering the optimal power density. However, in a strongly alkaline solution, EtOH is electrochemically converted to acetate through an incomplete C2-4e pathway using Pd-based catalysts with a fairly low CO_2_ selectivity of 0.5–7.5%, which severely jeopardizes the DEFCs performance^13,16^. Much effort has been directed toward improving the activity and stability of Pd-based catalysts based on the structure and electronic advantages of hybrid materials and structures, such as spatial confinement effect in the nanoscale composites^17^, catalyst-support interactions^18,19^, metal-metal interactions in the alloyed materials^20^, and electronic effect^21^.

The catalytic interface has to be properly engineered in order to ensure the successful delivery of those beneficial effects, shift the EOR pathway from C2-4e to C1-12e^22^, and thereby improve the activity, stability, and selectivity of the catalysts. However, the traditional approaches to physically mixing metal catalysts with carbon supports fail to engineer the catalytic interface homogeneously and controllably^23^. Thus, an ingenious way to engineer the catalytic interface of the materials for the EOR C1-12e pathway and the accelerated ORR kinetics is urgently needed but not yet achieved to push the development of DEFCs technology. The carbon supports play a vital role in catalytic interface engineering. The carbonaceous materials derived from the pyrolysis of zeolite imidazole frameworks (ZIFs, such as Zn-based and Co-based) have been used for catalysis due to their high specific surface area, abundant porous structure, high electrical conductivity, and uniformly distributed active sites^24–26^. Moreover, the ZIFs-derived materials are widely used as a functional matrix to support active PGMs for electrocatalysis reactions. For example, Co-based ZIF (ZIF-67) was widely used to produce Co and N co-doped carbon materials to support Pd nanoparticles, which were developed for hydrogen generation^27^, hydrogenation^28^, ORR^29^, and EOR^30^. However, the Co NPs existed on the surface of the ZIF-67-derived carbon materials are easy to form an alloy with Pd, which is unfavorable for the engineered catalytic interface. In contrast, the nitrogen-doped carbon from thermolysis of Zn-based ZIF (ZIF-8) is usually amorphous with abundant defects which are not stable during electrocatalysis reaction^31^. It has been confirmed that the metal sites supported on amorphous carbon with abundant defects face the problem of demetallation during the ORR due to the easy oxidation of such defect-rich carbon and thus show poor stability^32^. And the low electric conductivity of amorphous carbon support impedes electron transport, leading to poor electrocatalytic performance. Graphitized carbon structures with better electric conductivity have high corrosion/oxidation resistance to stabilize the metal active sites^33^. However, a high graphitization degree means insufficient defects to host and anchor metal active sites. Therefore, it is still a challenge to regulate the local structure, coordination environment, and graphitization degree of carbon materials to deal with the above trade-off problems^34^. We recently found that the local coordination environment of Pd in Pd/N-C can be modified by the introduction of F atoms, forming an N-rich Pd surface, which is favorable for inhibiting Pd migration and decreasing carbon corrosion when used for DEFC^12^. Inspired by this work, we would like to develop an easier approach to designing catalysts for DEFC by considering the interface effect and engineering between catalyst and support.

Herein, we propose an interface regulation strategy to engineer the synergistic interface between Pd catalyst and cobalt nanoparticles (NPs) coated by nitrogen-doped highly graphitic carbon (Co@N-C) for boosting EOR and ORR activities in alkaline DEFCs. The Co NPs in Co@N-C promote the transformation of amorphous carbon to highly graphitic carbon on Co NPs surfaces, thus establishing the spatial confinement on Co@N-C due to the Co NPs coated by highly graphitic N-C. Such spatial confinement effect not only suppresses the agglomeration of Co NPs and mitigates the collapse of the microporous structures, but also inhibits the Co leaching into the electrolyte when it is subjected to long-time operation or accelerated stability tests (AST). The trace amount of Pd catalyst was in-situ embedded in the Co@N-C, ensuring the strong synergistic catalyst (Pd)-support (Co@N-C) interaction and electronic effect between Pd and N. The electron transfer was prominently improved across the proposed synergistic interface within the spatially confined Pd/Co@N-C hybrid catalyst, promoting the cleavage of C-C bonds during EOR. The Pd/Co@N-C exhibits much higher mass/specific activity for EOR through a direct C1-12e pathway. In contrast, the inefficient C2-4e pathway was observed on Pd/C, Pd/N-C, and physically mixed Pd+Co@N-C catalysts without the synergistic interface. Furthermore, this Pd/Co@N-C with a synergistic interface also shows improved ORR activity than the control samples. When tested in DEFCs using Pd/Co@N-C as both anode and cathode catalyst, a maximum power density of 438 mW cm^−2^ is achieved with more than 1000 hours of operation, which is several-fold higher than that of Pd/C, Pd/N-C, and Pd+Co@N-C at the same Pd loading (1 mg cm^−2^). This work presents an ingenious design and comprehensive study of the synergistic interface using Pd/Co@N-C as a model catalyst for alkaline DEFCs, which opens an avenue for the rational design of efficient catalysts for a renewable and sustainable energy future.

## Results

### Spatial confinement effect and role of Co

**Fig. 1:**
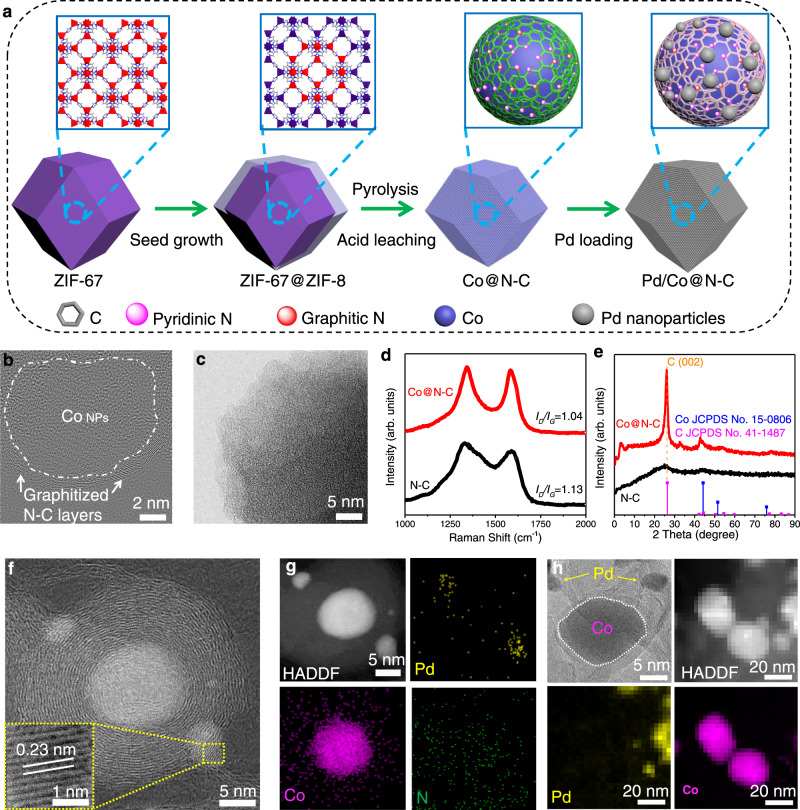
Schematic illustration of the synthesis process of Pd/Co@N-C. **a** The core-shell structured ZIF-67@ZIF-8 was synthesized, after pyrolysis and acid washing, the Co@N-C with spatial confinement effect was obtained. The Pd was supported on Co@N-C by an in-situ microwave reduction method to form a strong interface effect between Co@N-C and Pd. TEM images of (**b**) Co@N-C and (**c**) N-C. **d** Raman spectra and **e** XRD patterns of Co@N-C and N-C. **f** High-magnification bright field (BF)-STEM image. The inset of (**f**) shows the enlarged STEM image of Pd. **g** HAADF-STEM and the corresponding EDS elemental mappings of Pd, Co, and N. **h** HR-TEM, HAADF-STEM, and the corresponding EDS elemental mappings of Pd and Co of Pd+Co@N-C.

The synthesis process of Pd/Co@N-C is schematically illustrated in Fig. 1a and the experimental details are described in Methods. To obtain the spatial confinement effect of Co@N-C, a highly porous ZIF-67 was synthesized and then coated with a layer of ZIF-8 on the surface^24^. Thus, a dodecahedral core-shell structure of ZIF-67@ZIF-8 was obtained (Supplementary Figs. 1–6). After the pyrolysis of ZIF-67@ZIF-8 at 950 °C, the Co NPs covered by a few layers of nitrogen-doped highly graphitic carbon on the surface were formed (Co@N-C, Fig. 1b and Supplementary Fig. 7). In contrast, amorphous carbon was found on N-C (Fig. 1c) from the direct pyrolysis of ZIF-8. Raman spectra (Fig. 1d) show that the *I*_*D*_*/I*_*G*_ value (*I*_*D*_ and *I*_*G*_ denote the intensities of Raman peaks corresponding to the *D*-band and *G*-band, respectively) of Co@N-C (1.04) is smaller than that of N-C (1.13), indicating the reduced defects and increased degree of graphitization of carbon after Co introduction. The XRD patterns further verified this point, in which a sharp peak located at ~ 26° that belongs to graphite C (002) was found on Co@N-C (Fig. 1e), whereas amorphous carbon was found on N-C. Thus, the presence of cobalt is beneficial for increasing the graphitization degree of carbon^25^. These nitrogen-doped highly graphitic carbon layers with encapsulated Co NPs can be used as ideal support due to the much higher electric conductivity than amorphous N-C (Supplementary Fig. 8).

Nevertheless, it has been proved that if the graphene layers are too thin, the materials may encounter the gradual oxidation and corrosion of the graphitic structure, leading to the performance decay of fuel cells operated under harsh electrochemical environments^32,35^. Thus, in order to maintain the structural stability of carbon that provides enough thickness for the proposed study on engineering the synergistic interface^36^, the optimized thickness of graphitic layers was obtained by tuning the surface ZIF-8 content in ZIF-67@ZIF-8 (see Methods). In contrast, without coating ZIF-8 on ZIF-67 under pyrolysis, the obtained Co-N-C is mostly configured as Co NPs supported on N-C rather than the formation of N-C shell on the surface of Co NPs (Supplementary Fig. 9a), which matches well with previous reports^28–30^. However, Co NPs were leached out from the Co-N-C after acid washing (Supplementary Fig. 9b). Besides, when using the Co-N-C (without acid washing) as a matrix to support 5 wt.% Pd, the galvanic replacement reaction between Co and Pd^2+^ will happen, irreversibly causing the formation of PdCo alloy (denoted as Pd/Co-N-C, Supplementary Fig. 10). The lack of spatial confinement effect in Pd/Co-N-C results in a serious performance decay (Supplementary Fig. 11a) and quick Co leaching into the electrolyte during ORR (Supplementary Fig. 11b) due to the nude Co NPs without N-C protection. This spatial confinement effect can suppress the agglomeration of Co NPs, thus providing ideal support with intimately exposed interfaces with high specific surface areas and avoiding the Co leaching issue during the electrocatalytic reaction. Both the ZIF-67@ZIF-8 and Co@N-C showed negligible activity towards EOR (Supplementary Fig. 12) and ORR (Supplementary Fig. 13), indicating that the single support is non-active towards catalytic reactions (especially for the EOR) and needs to be further decorated by PGMs.

### The catalyst-support effect between Pd and Co@N-C

A strong catalyst-support effect is desired, which can enhance the catalytic activity, stability, and selectivity^37^. While the weak bonding between Pd NPs and pure carbon support interface has been proved^38,39^. To strengthen the interaction between catalyst and support for better serving electrocatalytic reactions, the Pd catalyst was then embedded in the surface of Co@N-C by an in-situ microwave reduction method (See Methods), with a Pd and Co loading of 4.98 wt.% (weight percentages) and 10.38 wt.% from inductively coupled plasma mass spectrometer (ICP-MS), while N (2.6 wt.%) and C (82.04 wt.%) from elementary analysis, respectively, in the Pd/Co@N-C (Supplementary Tables 1 and 2). Aberration-corrected scanning transmission electron microscopy (STEM) image (Fig. 1f), high-angle annular dark-field (HAADF)-STEM, and the corresponding elemental mapping images (Fig. 1g) show that the Pd NPs with an average size of 3 nm (Supplementary Fig. 14) are semi-embedded in the carbon layers to form Pd/Co@N-C, configuring intimate Pd and Co@N-C interfaces/boundaries, which ensures not only sufficiently exposed surface area/active sites but seamless contact/interaction of Pd with the Co@N-C support for the catalytic reactions^38,40^. Moreover, a lattice spacing of 0.23 nm (inset in Fig. 1f) revealed by the enlarged STEM image of Pd/Co@N-C corresponds to the Pd (111) facet^41^. Note that a trace amount of Zn impurity (~0.01 at%, Supplementary Fig. 15 and Supplementary Table 2) remained in the sample after forming porous graphitic structure^42,43^, which shows no activity towards EOR (Supplementary Fig. 12) and ORR (Supplementary Fig. 13).

To reveal the strong catalyst-support effect, a control sample Pd+Co@N-C (physically mixed Pd NPs with Co@N-C support) with 5 wt.% Pd loading was further prepared and studied (Supplementary Table 2 and Methods). Figure 1h shows the HR-TEM and elemental mapping images of Pd+Co@N-C. Unlike the Pd/Co@N-C with a close Pd contact with Co@N-C (Fig. 1f, g), the Pd NPs in the Pd+Co@N-C sample prefer to adhere to the N-C surface (Fig. 1h) due to the electrostatic attraction between Pd NPs and defective carbon^44,45^. The physically mixed Pd+Co@N-C sample cannot provide a strong catalyst-support effect because of the poorly formed interface. Besides Pd+Co@N-C, commercial Pd/C (10 wt.%) and Pd/N-C (5 wt.%) were used to further verify the catalyst-support effect (Supplementary Fig. 16, Supplementary Table 2, and Methods). X-ray diffraction (XRD) patterns (Supplementary Fig. 17) show that Pd in all samples has a face-centered cubic structure, consistent with the STEM results (Fig. 1f–h). The strong peaks located at 26° correspond to the highly graphitic carbon from the Co@N-C support in both Pd/Co@N-C and Pd+Co@N-C samples (Fig. 1e). In addition, the Raman spectra (Supplementary Fig. 18) indicate more defects of carbon presented in N-C due to higher *I*_*D*_*/I*_*G*_ value of Pd/N-C (1.13), while a reduced *I*_*D*_*/I*_*G*_ ratio of 1.04 was found on Pd/Co@N-C, which matches well with Fig. 1d. On the other hand, the physically mixed Pd+Co@N-C shows a similar *I*_*D*_*/I*_*G*_ value to that of Pd/Co@N-C due to the N-doping effect in the same Co@N-C support. The Pd/C shows the lowest *I*_*D*_*/I*_*G*_ ratio (0.92) due to the least carbon defects, however, the absence of N in Pd/C results in poor performance^46^. As demonstrated by previous works^25,34,46^, an increased graphitic-N content can boost the activity and stability of the catalyst.

### Electronic effect between Pd and Co@N-C support

**Fig. 2:**
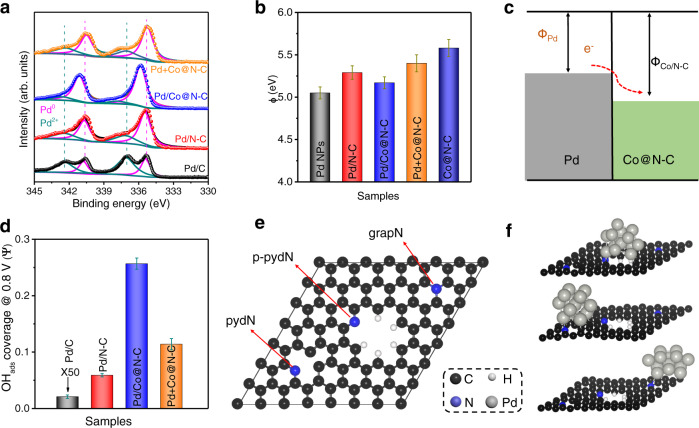
Strong electronic effect between Pd and Co@N-C. **a** Pd 3*d* XPS spectra. **b** The WF (ɸ) of Pd/C, Pd/N-C, Pd/Co@N-C, physically mixed Pd+Co@N-C, and Co@N-C support, respectively. **c** Schematic illustrations of electronic interfacial interactions occurring between Pd and Co@N-C. **d** The oxygenophilic properties at 0.8 V_RHE_ for different samples. The error bars in (**b**) and (**d**) represent the s.d. of at least three independent measurements, and the data were presented as mean values ± s.d. **e** Atomistic structure of a nitrogen-doped graphene layer containing grapN, pydN, and p-pydN sites. **f** Optimized atomic structures of a thirteen-atom Pd_13_ cluster adsorbed on different N sites of a nitrogen-doped graphene layer.

**Fig. 3:**
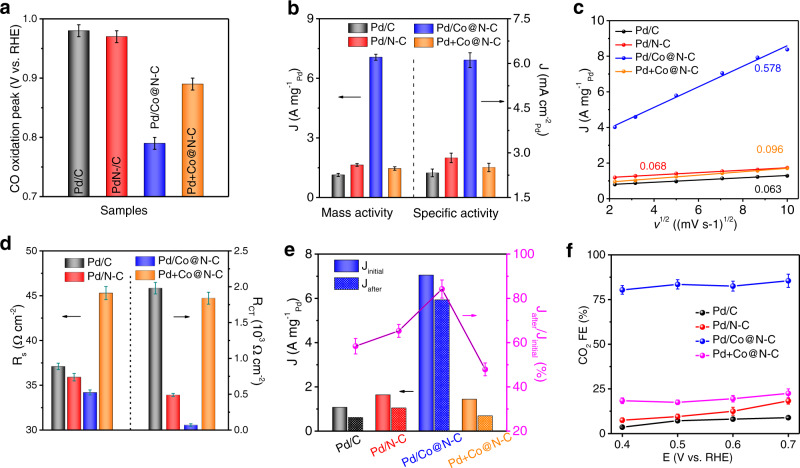
Synergistic interface effect of Pd/Co@N-C for electrocatalytic EOR performance. **a** The CO oxidation peak on different samples with a scan rate of 20 mV dec^−1^. **b** Peak current density *(J)* normalized to Pd loading (left) and ECSA (right) of Pd/C, Pd/N-C, Pd/Co@N-C, and Pd+Co@N-C. **c** *J* as a function of the square root of the scan rate (v^1/2^). **d** The system resistance (R_s,_ left) and charge transfer resistance (R_CT_, right) at 0.6 V_RHE_ for different samples. **e** Left axis: EOR mass activity before (solid bars) and after (dashed bars) AST for 500 cycles. Right axis: Percentage ratio of J_after_ (mass activity after AST) to J_initial_ (initial mass activity before AST). **f** Faradic efficiency (FE) of CO_2_ for EOR at different potentials. The error bars in (**a**, **b**, **d**, **e**, **f**) represent the s.d. of at least three independent measurements, and the data were presented as mean values ±s.d.

**Fig. 4:**
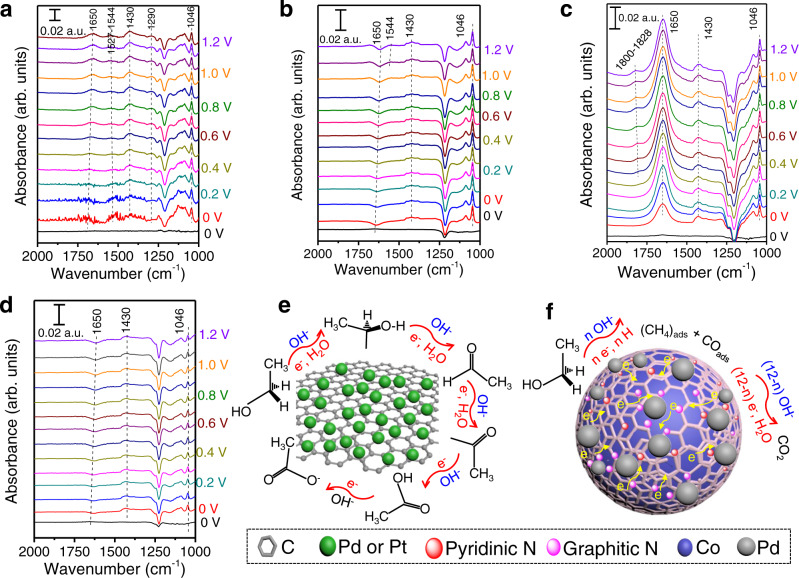
Mechanistic study of reaction pathway for EOR based on in-situ ATR-SEIRAS measurements. In-situ ATR-SEIRA spectra measured at different potentials for **a** Pd/C, **b** Pd/N-C, **c** Pd/Co@N-C, and **d** Pd+Co@N-C in 0.1 M KOH + 1.0 M EtOH aqueous solution. The reference spectra (black lines) in (a-d) were obtained at 0 V_RHE_ in 0.1 M KOH aqueous solution. The proposed EOR mechanisms for **e** Pd/C (or Pt/C, Pd/N-C, Pd+Co@N-C) and **f** Pd/Co@N-C in alkaline solution. The Pd loading on Au modified Si electrode was 0.02 mg cm^−2^. Note that three-layer graphitic carbon was shown in **f** to conceptually illustrate the proposed mechanism. An actual catalyst may have more graphitic layers.

X-ray photoelectron spectroscopy (XPS) was used to study the chemical and electronic state of the materials. The XPS Pd 3*d* peaks of Pd/Co@N-C shift to the higher binding energy of about 0.5 eV compared to Pd/C, Pd/N-C, Pd/Co@C, and Pd+Co@N-C (Fig. 2a), although the binding energy of Pd in Pd/Co@N-C is positively shifted compared to the reference samples, no oxide (PdO_x_) was formed in Pd/Co@N-C, and the predominant presence of the Pd is still in metallic phase (Pd°) (Supplementary Table 3)^9^, indicating that the Pd is electron-deficient in Pd/Co@N-C. Correspondingly, the XPS N 1 *s* peaks of Pd/Co@N-C shift to the lower binding energy of ca. 0.4 eV compared to Pd/N-C, Pd+Co@N-C, and Co@N-C support (Supplementary Fig. 19a). In addition, compared to Pd/N-C sample, much higher graphitic-N content was found on Pd/Co@N-C, Pd+Co@N-C, and Co@N-C, indicating that the presence of cobalt is beneficial for increasing the graphitic-N content, which is consistent with XRD, Raman, and TEM results. However, the XPS Co 2*p* peaks of Pd/Co@N-C have no obvious shift (Supplementary Fig. 19b), indicating the strong electronic effect between Pd and N in the Pd/Co@N-C^47^. As suggested by theoretical prediction^48^, N-doping is a prominent way to strongly anchor Pd NPs^49^, and tune the adsorption of reaction intermediates for improving the reaction activity. With expectation, the Pd/Co@C samples without N-doping show a much inferior performance and further verify the significance of the electronic effect (Supplementary Fig. 20). In contrast, Pd in the commercial Pd/C is mainly in the oxidized state due to the different preparation method^9^. The Pd/N-C, Pd/Co@C, and Pd+Co@N-C have a similar Pd^0^ content but inferior electrochemical performance compared to Pd/Co@N-C (Fig. 3, Fig. 4, and Supplementary Fig. 20g-i), confirming that the absence of Co, or N, or the traditional physical-mixing methods fail to form the synergistic interface/boundary in the catalysts even with similar Pd^0^ contents. To evaluate the impact of the oxidation state on EOR activity, the Pd/Co@N-C with a similar oxidation degree to commercial Pd/C was prepared through annealing Pd/Co@N-C in the atmosphere (see Methods). The annealed sample was labeled as O-Pd/Co@N-C (Supplementary Fig. 21a and Supplementary Table 3) with a high Pd^2+^ content in the Pd/Co@N-C, which shows an improved anti-CO poisoning property, but a decreased EOR activity (Supplementary Fig. 21b-d) compared to the pristine Pd/Co@N-C. However, the O-Pd/Co@N-C still demonstrates superior performance than Pd/C, Pd/N-C, and Pd+Co@N-C, indicating that the metallic Pd is the main active phase for EOR, while the oxidized and the electrophilic Pd is beneficial for CO oxidation.

Furthermore, ultraviolet photoelectron spectroscopy (UPS) was employed to estimate the work function (ɸ) of the catalysts (Supplementary Fig. 22). The Pd NP has the smallest ɸ (5.08 eV, Fig. 2b), while Co@N-C has the higher ɸ (5.58 eV). After Pd embedment in the graphitic layer of Co@N-C, the electron will transfer from Pd to Co@N-C (Fig. 2c), which results in a ɸ of 5.17 eV in Pd/Co@N-C. These results match well with the above XPS data that the binding energy of Pd in Pd/Co@N-C is positively shifted compared to the reference samples. In contrast, the ɸ of physically mixed Pd+Co@N-C, Pd/N-C, and Pd/Co@C without N-doping are as high as 5.40, 5.29, and 5.38 eV, respectively (Fig. 2b, Supplementary Fig. 22, and Supplementary Table 3). The much lower ɸ of Pd/Co@N-C than other control samples indicates the promoted electron transfer in the Pd/Co@N-C^50^. In addition, the electron-deficient state of Pd in Pd/Co@N-C results in a much stronger oxophilic ability than the other control samples (Supplementary Fig. 23). Specifically, the OH_ads_ coverage at 0.8 V_RHE_ (where RHE represents reversible hydrogen electrode) is 0.26 (Fig. 2d), much higher than Pd/C (0.00042), Pd/N-C (0.06), and Pd+Co@N-C (0.11). The electron-deficient state of Pd is beneficial for the oxidation/removal of CO (poisoning intermediate species) and thus will boost the EOR performance, which we will discuss in detail in the following section.

To gain insight into the interface synergy between Pd NPs and carbon support (i.e., strong catalyst-support effect), we performed the first-principles density  functional theory (DFT) calculations. As shown in Supplementary Fig. 16, the Pd/Co@N-C catalyst shows a structure with Pd NPs semi-embedded in the nitrogen-doped carbon layer, whereas the Pd NPs are physically attached to the nitrogen-doped carbon layer in the Pd+Co@N-C catalyst. Consequently, we constructed a graphene layer containing three types of nitrogen sites, i.e., graphitic nitrogen (grapN), pyridinic nitrogen (pydN), and pyridinic nitrogen hosted by a large pore (p-pydN) as shown in Fig. 2e. A thirteen-atom cuboctahedral Pd_13_ cluster embedded in the p-pydN site was used to model the structure of Pd/Co@N-C, whereas the Pd_13_ cluster laid on the grapN or pydN site was used to model the structure of Pd+Co@N-C (Fig. 2f). Supplementary Table 4 reports the calculated adsorption energies of the Pd_13_ cluster on the different N sites. We found that the Pd_13_ cluster was energetically favorable to be adsorbed on the p-pydN site with adsorption energy (Ea) of −2.70 eV, as compared to on the pydN site (Ea = −2.13 eV) and grapN sites (Ea = −1.49 eV), suggesting that Pd clusters anchored on the p-pydN site are the most stable against aggregation. This result explains why Pd/Co@N-C exhibits higher durability than the Pd+Co@N-C catalyst in the long-term stability test. In addition, we conducted the Bader charge analysis^51^ to investigate the electron transfer between the Pd cluster and carbon substrate. The number of electrons transferred from Pd to carbon substrate was predicted to be 1.61, 1.06, and 1.03, for the Pd_13_ cluster on the p-pydN, pydN, and grapN sites, respectively (Supplementary Table 4). This result agrees well with our XPS result that Pd in Pd/Co@N-C shows an electron-deficient state as compared to other (Pd+Co@N-C) catalysts.

### Boosting EOR on Pd/Co@N-C with synergistic interface effect

The catalyst-support effect resulting from the strong contact/interaction between Pd and Co@N-C is beneficial for the electron transfer, thus boosting the electrocatalytic performance^22,23,52^. To testify the significance of the catalyst-support effect at the Pd/Co@N-C interface/boundary, electrochemical analyses were performed. CO stripping (Supplementary Fig. 24) was used to calculate the electrochemically active surface area (ECSA, Supplementary Fig. 25). The ECSA of Pd/Co@N-C (117.9 m^2^ g^−^^1^_Pd_) is much higher than those of Pd/C, Pd/N-C, Pd+Co@N-C, and other benchmarking catalysts (Supplementary Table 5), suggesting well-exposed active surfaces in Pd/Co@N-C for catalytic reactions. Moreover, the Randles-Sevcik equation was used to evaluate the electronic effect as well as the contribution of different N-C supports on the ECSA, and the same trend was found for all samples (Supplementary Fig. 26 and Supplementary Note 2). In addition, Pd/Co@N-C exhibits the most negative peak for CO oxidation (0.79 V_RHE_) than other samples (Fig. 3a and Supplementary Fig. 27) due to its stronger O/OH adsorption ability (Fig. 2d), indicating a better resistance to CO poisoning.

The EOR performance of Pd/Co@N-C was then assessed and compared with Pd/C, Pd/N-C, and Pd+Co@N-C by testing CV curves in 1 M KOH + 1 M EtOH solution (Supplementary Fig. 28). The peak mass and specific activity of Pd/Co@N-C (7.05 A mg^−1^_Pd_ and 6.11 mA cm^−^^2^) are 3.2–5.3 times higher than those of Pd/C, Pd/N-C, and Pd+Co@N-C (Fig. 3b), outperforming most of the benchmarking catalysts (Supplementary Table 5). This verifies that the Pd/Co@N-C with an intimate contact interface/boundary between Pd and Co@N-C is much more active than other control catalysts. It should be noted that the commercial Pd/C shows the worst performance among the catalysts, which might be due to different metal loading forms and the bigger mean nanoparticle size of commercial Pd/C (8 nm compared to 3 nm in Pd/Co@N-C). The EOR current density *(J)* increases drastically while the anodic peaks in the forward scan gradually shift to high potential with scan rate (Supplementary Fig. 29). The linear relationship between *J* and the square root of the scan rate (v^1/2^) indicates the diffusion-controlled electrochemical process on the surface of the catalysts (Fig. 3c). The much higher slope of Pd/Co@N-C (0.578) than Pd/C (0.063), Pd/N-C (0.068), Pd/Co@C (0.132), and Pd+Co@N-C (0.096) proves the facilitated electron transfer for EOR due to the as-proposed synergistic interface in the Pd/Co@N-C. To further investigate the effect of the different supports, the electrochemical impedance spectrum was further analyzed (Supplementary Fig. 30). The system resistance (R_S_) and charge transfer resistance (R_CT_) of Pd/Co@N-C are much lower than Pd/C, Pd/N-C, and Pd+Co@N-C (Fig. 3d), suggesting the greatly promoted electron transfer in Pd/Co@N-C. In contrast, Pd+Co@N-C shows the largest R_S_ and R_CT_ due to the poor catalyst-support interface formation by physical mixing, further validating the critical role of the catalyst-support effect in improving the electrocatalytic performance^37^.

The strong synergistic interface in Pd/Co@N-C is not only beneficial for electron transfer but also results in promoted C-C bond cleavage during EOR as well as leading to superior durability. The Pd/C, Pd/N-C, Pd/Co@N-C, and Pd+Co@N-C were subjected to continuous cycling for 500 cycles within 0.3–1.2 V_RHE_ (Supplementary Fig. 31) to evaluate the EOR stability. After 500 cycles, the Pd/Co@N-C (Fig. 3e) shows a much higher mass activity retention of ~84.2% than Pd/C (58.4%), Pd/N-C (65.3%), and Pd+Co@N-C (47.9%). Besides, the current density of Pd/C, Pd/N-C, and Pd+Co@N-C degraded almost to zero after 3 h of chronoamperometry test due to the accumulation of CO poisoning, whereas the Pd/Co@N-C preserved a high current density of 0.92 A mg^−^^1^_Pd_ due to better anti-CO poisoning properties (Supplementary Fig. 32). The steady-state oxidation current of Pd/Co@N-C (Supplementary Fig. 33) shows no obvious decay during five consecutive reactivation cycles, further proving the good stability and anti-poisoning performance due to the presence of the synergistic interface in the Pd/Co@N-C.

Furthermore, the potential-dependent EOR selectivity of Pd/Co@N-C was investigated to explore the electron transfer pathway based on the electronic effect during electrocatalytic reactions. Since the high potentials that exceed 0.8 V_RHE_ in three-electrode half-cell were useless in real DEFC device (Supplementary Fig. 34 and Supplementary Note 3), the Faradic efficiency (FE) for EOR was tested at low potentials from 0.4 to 0.7 V_RHE_. Note that the produced CO_2_ from EOR will react with KOH, the FE of CO_2_ was therefore determined by titration method^12^ (Supplementary Fig. 34c). While the liquid products (if any) of EOR were analyzed by ^1^H nuclear magnetic resonance (^1^H-NMR, Supplementary Fig. 34d). The peaks with the chemical shift at δ = 1.9 ppm in the ^1^H-NMR spectra were ascribed to acetate (CH_3_COO^−^). Since there are no other peaks in the spectra, the main gaseous and liquid products from EOR are CO_2_ and CH_3_COO^−^, respectively. The FE of CO_2_ and CH_3_COO^−^ were calculated, as shown in Fig. 3f and Supplementary Fig. 35. In particular, the FE of CO_2_ is 80.4%, 83.5%, 82.5%, and 85.5%, at 0.4, 0.5, 0.6, and 0.7 V_RHE_ over the Pd/Co@N-C catalyst, which is much higher than the Pd/C, Pd/N-C, and physically mixed Pd+Co@N-C (all <20%). On the other hand, the FE of CH_3_COO^−^ is less than 20% over the Pd/Co@N-C catalyst at the studied potentials (Supplementary Fig. 35), much lower than Pd/C, Pd/N-C, and Pd+Co@N-C (all >80%). These results prove that the EOR on Pd/Co@N-C with the strong synergistic interface is the direct C1-12e pathway, whereas all other control samples without the synergistic interface and structural effects (Pd/C, Pd/N-C, and Pd+Co@N-C) are the C2-4e pathway. Moreover, our theoretical study (Supplementary Note 4 and Supplementary Fig. 36) predicts that electron deficient state of Pd NPs in Pd/Co@N-C could accelerate the rate-determining step of ethanol oxidation to CO_2_, explaining the enhanced EOR activity of Pd/Co@N-C catalyst (Fig. 3).

To further verify the merit of the proposed interface effects (spatial confinement, catalyst-support, and electronic effects) and interface engineering are widely applicable to other PGMs-based catalysts, the Pt/Co@N-C was prepared (see Methods). the EOR peak current density of Pt/Co@N-C is as high as 8.84 A mg^−1^_Pt_ (Supplementary Fig. 37), ca. 5.1-times higher than the physically mixed Pt+Co@N-C (1.44 A mg^−1^_Pt_). Moreover, Pt/Co@N-C shows a much higher EOR performance than Pd/Co@N-C (Supplementary Fig. 38) due to the Pt being a more oxophilic metal than Pd^53^. Hence, this confirms the efficacy of our proposed interface synergism strategy with alternative materials for EOR.

### EOR mechanism on the synergistic interface of Pd/Co@N-C

To investigate the EOR mechanism and reaction pathway at the molecular level^54,55^, in situ attenuated total reflection surface-enhanced infrared radiation absorption spectroscopy (ATR-SEIRAS, Supplementary Fig. 39) was employed to identify the intermediate species on Pd/C, Pd/N-C, Pd/Co@N-C, and Pd+Co@N-C (Fig. 4a–d) with a Pd loading of 0.02 mg cm^−2^ on Au modified Si electrode (See Methods). The Au film without Pd/Co@N-C shows one order of magnitude lower EOR current density and much higher oxidation potential (ca. 1.2 V_RHE_) than Pd/Co@N-C (Supplementary Fig. 40a-b), and the spectrum on Au/Si prism at different potentials (from 0 to 1.0 V_RHE_) in 0.1 M KOH with 1 M EtOH show no obvious changes compared to that in 0.1 M KOH (Supplementary Fig. 40c-d), indicating that there is almost no EOR activity on the Au/Si prism electrode before 1.0 V_RHE_. Thus the peaks discussed in the following are associated with the surface adsorbates information from Pd/Co@N-C catalyst, rather than from Au or Si (see detailed analysis in Supplementary Note 5). The characteristic peak for the C-OH stretching vibration of CH_3_CH_2_OH at 1046 cm^−1^ was observed for all catalysts (Supplementary Fig. 41). The peak at 1290 cm^−1^ belonging to the CO stretch in –COOH/-COO^−^^56,57^ as the incomplete products was identified both on Pd/C (Fig. 4a), Pd/N-C (Fig. 4b), and Pd+Co@N-C (Fig. 4d) due to the incomplete EOR, which was absent in the Pd/Co@N-C (Fig. 4c). The peak at 1430 cm^−1^ is found on all samples and can be attributed to the Au/Si signals (Supplementary Fig. 40d). The peaks located at 1527-1544 cm^−1^ on Pd/C (Fig. 4a), Pd/N-C (Fig. 4b), and Pd+Co@N-C (Fig. 4d) correspond to the acetate (OCO stretch) from the incomplete C2-4e reaction pathway^56^. In contrast, this characteristic peak was also absent on Pd/Co@N-C (Fig. 4c) due to the complete C1-12e EOR pathway on Pd/Co@N-C. The peak at 1650 cm^−1^ is found on all samples and can be ascribed to the exclusion of interfacial H_2_O by incoming CH_3_CH_2_OH^58^. The peaks at 1800-1828 cm^−1^ belonging to bridge-bonded CO_ads_^55,58^ were found on Pd/Co@N-C in Fig. 4c when the applied potential was higher than 0.5 V, arising from the C-C bond cleavage, which can’t be found in other control samples.

When further increasing the Pd loading from 0.02 to 0.5 mg cm^−2^ on Au modified Si electrode, the C-C bond cleavage happened at a much lower potential of 0.2 V_RHE_ (Supplementary Fig. 42a), and this characteristic CO peak was found at a wide potential of 0.2-0.9 V_RHE_ due to the continuous C-C bonds cleavage. The CO signal disappeared at above 0.9 V_RHE_ (Supplementary Fig. 42a) while the CO_2_ characteristic peak can be seen and become stronger as the potential increased from 1.0 to 1.4 V_RHE_ (Supplementary Fig. 42b), indicating the C-C bond was easily broken at the low potential on Pd/Co@N-C and finally be oxidized to CO_2_ through a direct C1-12e pathway at a high potential. Meanwhile, the acetate was not found from the Pd/Co@N-C by H^1^-NMR (Supplementary Fig. 34d). Nevertheless, acetate was detected as the liquid product after incomplete EOR using the Pd/C, Pd/N-C, and Pd+Co@N-C by ^1^H-NMR spectroscopy. Furthermore, the isotopically labeled measurement for EOR on Pd/Co@N-C (Supplementary Fig. 43 and Supplementary Note 6) further confirmed the above in-situ ATR-SEIRAS results. The ^13^C-NMR results strongly indicated that the ^13^CO_3_^2-^ is the main EOR product on Pd/Co@N-C rather than CH_3_^13^COO^−^ (Supplementary Fig. 43c). Besides, the signals of ^13^CO_3_^2-^ at 171 ppm and CH_3_^13^COO^−^ at 181 ppm are potential-dependent, indicating that these products are from EtOH oxidation, rather than contaminate of atmosphere or support corrosion. Thus, the Pd/Co@N-C with the synergistic interface is beneficial for complete EOR with a C1-12e pathway. In contrast, the incomplete C2-4e reaction pathway was found in the control samples, which matches well with the above electrochemical performance tests.

Based on the advanced characterizations discussed above, the EOR mechanisms and electron transfer pathways for the Pd/Co@N-C catalyst are proposed (Supplementary Note 1). On the surfaces of Pd/C, Pd/N-C, and Pd+Co@N-C (Fig. 4e) or even on the Pt/C surface (Supplementary Fig. 44), the EOR follows the 4e pathway and the RDS is the removal of the adsorbed acyl by the adsorbed hydroxyl with acetate as the final products^13,59^. On the other hand, the Pd/Co@N-C (Fig. 4f) follows the C1-12e pathway for EOR^54^, and the C-C bond is cleaved on the Pd/Co@N-C when the applied potential is above 0.2 V_RHE_ with high Pd loading (Supplementary Fig. 42a), much lower than those of the reported Pt/Pd-based materials at above 0.9 V_RHE_^16^. The (CH_x_)_ads_ and CO_ads_^54^ are further oxidized to CO_2_ with an increase in the applied potential of ca. 0.7 V_RHE_ (Supplementary Fig. 42b). The oxidation of CO_ads_ becomes the RDS in which the OH_ads_ are necessary for the oxidation of CO_ads_. Proved by XPS results, the strong electronic effect between Pd and Co@N-C support, as well as the Pd with high oxophilic properties in the Pd/Co@N-C helps the strong adsorption of O_ads_/OH_ads_ (Fig. 2d), thus leading to the easy removal of CO_ads_ (Fig. 3a). In addition, both the oxidation of (CH_x_)_ads_ and CO_ads_ in the presence of water are thermodynamically and kinetically favorable than the C-C bond cleavage^16,60^. In contrast, Pd/C, Pd/N-C, and physically mixed Pd+Co@N-C show a 4e pathway for EOR, further confirming the indispensable synergistic interface for complete EOR via a C1-12e pathway.

### Boosting ORR on Pd/Co@N-C with synergistic interface effect

**Fig. 5:**
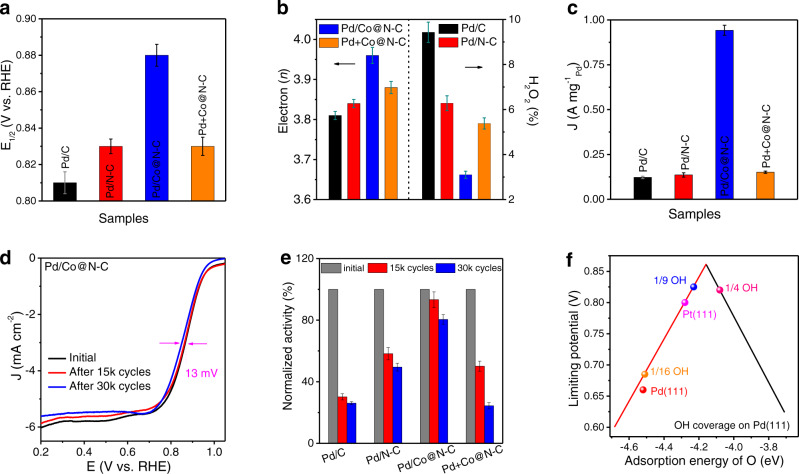
Synergistic interface effect of Pd/Co@N-C for electrocatalytic ORR performance. **a** ORR half-wave potential (E_1/2_) of different samples. RHE, reversible hydrogen electrode. V, the voltage without current (i)-resistance (R) compensation. **b** Electron transfer number (*n*, left) and H_2_O_2_ production (right) by RRDE test. **c** ORR mass activity at 0.9 V_RHE_. **d** ORR polarization curves of Pd/Co@N-C before and after AST for 15k and 30k cycles. **e** Normalized ORR initial mass activity, as well as after 15k and 30k cycles AST. **f** Volcano plot for ORR on Pd(111) with different OH coverages. The data to fit the volcano plot are adapted with permission from Ref. ^62^. Copyright 2018, American Chemical Society.

The interface synergism and structural effects, including the spatial confinement, strong catalyst-support, and electronic effects of Pd/Co@N-C will not only improve the EOR performance but also boost the ORR performance. The ORR activity of Pd/C, Pd/N-C, Pd/Co@N-C, and Pd+Co@N-C (Supplementary Fig. 45a) show increased ORR activity than Co@N-C (Supplementary Fig. 13b), confirming the active phase of Pd in delivering ORR. The Pd/Co@N-C shows a half-wave potential (E_1/2_) of 0.88 V_RHE_ (Fig. 5a), which is 50-90 mV more positive than those of Pd/C (0.79 V_RHE_), Pd/N-C (0.82 V_RHE_), and Pd+Co@N-C @Co (0.83 V_RHE_). Rotating ring-disk electrode (RRDE, Supplementary Fig. 45b) results prove an almost complete 4e ORR pathway with a minimal H_2_O_2_ yield of 3.5% over the Pd/Co@N-C catalyst (Fig. 5b). In contrast, the Pd/C, Pd/N-C, and Pd+Co@N-C all show much lower electron transfer numbers and much higher peroxide production (Fig. 5b), confirming the crucial role of the synergistic interface in promoting ORR. A mass activity of 0.94 A mg^−1^_Pd_ was achieved on the Pd/Co@N-C at 0.9 V_RHE_ (Fig. 5c), far surpassing the Pd/C (0.12 A mg^−1^_Pd_), Pd/N-C (0.14 A mg^−1^_Pd_), Pd+Co@N-C (0.15 A mg^−1^_Pd_), and most of the state-of-the-art ORR catalysts (Supplementary Table 6).

Moreover, the catalysts were further subjected to the AST within 0.6-1.0 V_RHE_ for 30k cycles at 100 mV s^−1^ in the O_2_-saturated 0.1 M KOH (Fig. 5d and Supplementary Fig. 46a-c). The Pd/Co@N-C retains 80.5% of the initial activity with only 13 mV of E_1/2_ loss after the test (Fig. 5d-e), which is much better than those of Pd/C (26.1%, 68 mV E_1/2_ loss), Pd/N-C (49.5%, 19 mV E_1/2_ loss) and Pd+Co@N-C (24.4%, 32 mV E_1/2_ loss). Furthermore, the long-term stability was also evaluated using a chronoamperometric response at 0.6 V_RHE_ in the O_2_-saturated 0.1 M KOH aqueous solution (Supplementary Fig. 46d). The Pd/Co@N-C retained more than 92.0% of the initial current density after 24 h, while quick performance decays were found on Pd/C, Pd/N-C, and Pd+Co@N-C, with current density retentions of ca. 77.9%, 85.4%, and 63.5%, respectively. The activity decay for Pd/C is mainly due to Pd deactivation caused by the loss of ECSA, particle aggregation, and Ostwald ripening^9,61^. Meanwhile, the structural integrity was well maintained due to the spatial confinement effect enabled by the synergistic interface in the Pd/Co@N-C (Supplementary Figs. 47-48). The central role of the synergistic interface in stabilizing the catalyst activity was proved by confirming the quick performance decay of the physically mixed Pd+Co@N-C, which does not show the spatial confinement effect (inhibition of Co leaching) as presented in Pd/Co@N-C.

Owing to the electron-deficient state of Pd NPs in the Pd/Co@N-C catalyst, we recognized that the Pd NPs showed a strong oxophilicity to adsorb electron-rich groups like hydroxyl (OH) on the surface (Fig. 2d). Consequently, we computationally examined how the OH coverage would affect the ORR activity of Pd(111) surface. In this study, we investigated three OH coverages on Pd(111), namely 1/4 (i.e., a quarter of Pd sites are covered by OH), 1/9, and 1/16. Figure 5f shows a volcano plot that depicts a relationship between the adsorption energy of O and the limiting potential of ORR. Here, the limiting potential of ORR is defined as the highest potential at which each elementary reaction of ORR becomes exothermic. Thus, a higher value of the limiting potential indicates a higher ORR activity^62^. We predicted the limiting potential of ORR to be 0.82, 0.82, 0.68, and 0.66 V, on the Pd(111) surface with OH coverage of 1/4, 1/9, 1/16, and without OH adsorbate, respectively. This result reveals that the introduction of OH adsorbate on the Pd(111) surface, due to the electron-deficient state of Pd NPs, could enhance the ORR activity, and an OH coverage between 1/4 and 1/9 would lead to the desired ORR activity (even higher than that of Pt) on Pd(111). Hence, our DFT results explain the enhanced ORR performance of the Pd/Co@N-C catalyst, due to its proper OH coverage than that of other catalysts (Fig. 2d).

### Direct ethanol fuel cell performance

**Fig. 6:**
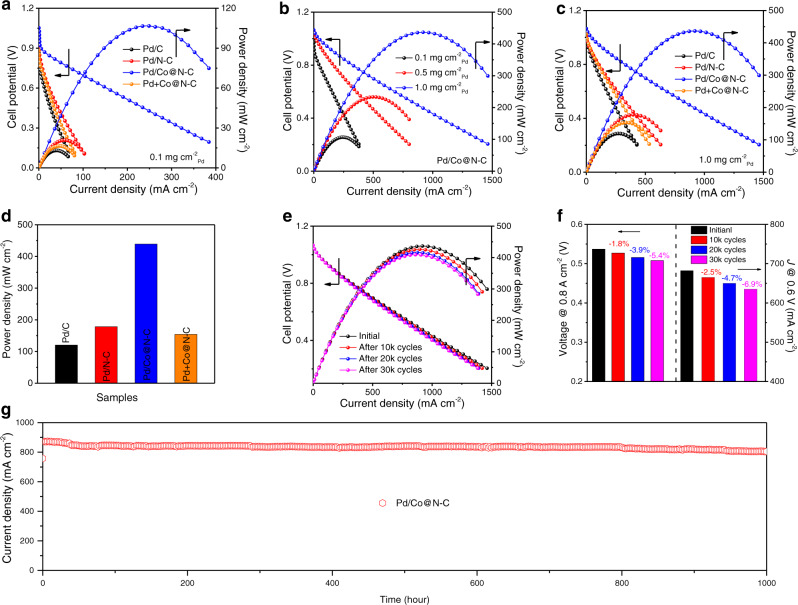
Synergistic interface effect of Pd/Co@N-C for DEFCs performance. **a** Steady-state DEFCs polarization and power density curves using different catalysts with a Pd loading of 0.1 mg cm^−2^ on MEA. **b** Steady-state DEFCs polarization and power density curves using Pd/Co@N-C with different Pd loading on MEA. **c** Steady-state DEFCs polarization and power density curves using different catalysts with a Pd loading of 1.0 mg cm^−2^ on MEA. **d** The maximum power density of DEFCs using different catalysts with a Pd loading of 1.0 mg cm^−2^ on MEA. **e** Steady-state DEFCs polarization and power density curves of Pd/Co@N-C after voltage cycling within 0.6-0.9 V for 10k, 20k, and 30k cycles AST. **f** The voltage loss at 0.8 A cm^−2^ (left) and current density loss (right) at 0.6 V after 10k, 20k, and 30k cycles AST. **g** The 1000 hours stability of DEFCs using Pd/Co@N-C as both anode and cathode catalysts (1 mg cm^−2^) at 0.5 V. The anode was fed with 1 M KOH + 2 M EtOH aqueous solution at a flow rate of 5 mL min^−1^; the cathode was fed with O_2_ at a flow rate of 100 mL min^−1^. The test temperature was 60 °C without backpressure.

To assess the practical application of Pd/Co@N-C with the proposed synergistic interface, a single fuel cell reactor (Supplementary Fig. 49) was assembled using Pd/Co@N-C as both anode and cathode to study the bifunctional EOR/ORR activities in DEFCs. Even with a low Pd loading of 0.1 mg_Pd_ cm^−2^ on membrane electrode assembly (MEA), an open-circuit voltage (OCV) of 1.05 V was obtained for Pd/Co@N-C (Fig. 6a), very close to the theoretical value of 1.14 V for the alkaline DEFCs^63^ and much superior to Pd/C (0.86 V), Pd/N-C (0.92 V) and Pd+Co@N-C (0.88 V). The Pd/Co@N-C delivers a maximum power density of 107 mW cm^−2^, which is 4.0-7.5 times higher than Pd/C (13 mW cm^−2^), Pd/N-C (21 mW cm^−2^), and Pd+Co@N-C (17 mW cm^−2^). The durability test of Pd/Co@N-C in DEFCs was evaluated by holding the cell voltage at 0.4 V for 100 hours (Supplementary Fig. 50). About 83.2% of the current density was retained, which is much more stable than other works (Supplementary Table 7). The FE of EOR products was estimated by analyzing the exhaust collected from the DEFCs anode at 0.4 V (Supplementary Fig. 51). The FE of CO_2_ and CH_3_COO^−^ were 87.6% and 9.2%, respectively, proving the main C1-12e pathway on the Pd/Co@N-C in DEFCs. While the main product was CH_3_COO^−^ on the Pd/C, Pd/N-C, and Pd+Co@N-C with a low FE of CO_2_ less than 25% (Supplementary Fig. 51e), agreeing well with the three-electrode test and previous reports^16,64^.

To decrease the local mass-transfer resistance during fuel cell operation, we further increase the Pd loading of Pd/Co@N-C from 0.1 mg cm^−2^ to 0.5 and 1.0 mg cm^−2^ on MEA. The thickness of both the anode and cathode catalytic layer for the freshly prepared Pd/Co@N-C MEA (1.0 mg cm^−2^) determined by cross-sectional SEM (Supplementary Fig. 52) was 74 ± 2 μm. While the total thickness of MEA is 465 ± 4μm, no delamination phenomenon was found, indicating good contact between the catalyst and membrane. The catalytic layer and MEA are much thinner than previously reported works^65^, which can thus ensure a promoted mass transfer. As shown in Fig. 6b, the maximum power density of Pd/Co@N-C was further increased, indicating the PGMs loading dependence of fuel cell performance. While the inferior fuel cell performance was observed in the control samples even with a Pd loading of 1 mg cm^−2^ (Fig. 6c). Especially, the maximum power density of 439 mW cm^−2^ is obtained on Pd/Co@N-C (Fig. 6d), which is several-fold higher than Pd/C (120.3 mW cm^−2^), Pd/N-C (178.5 mW cm^−2^), Pd+Co@N-C (154.3 mW cm^−2^) and much higher than the majority of the previously reported works (Supplementary Table 8), further verifying the significant performance improvement due to the synergistic interface effects in Pd/Co@N-C.

The long-term stability of DEFC was tested by the AST by cycling voltage within 0.6–0.9 V (Fig. 6e). The voltage drop was only 5.4% (29 mV) after 30k cycles at a current density of 0.8 A cm^−2^ (Fig. 6f, left), which satisfies the US Department of Energy (DOE) 2025 target^66^ (≤30 mV loss at 0.8 A cm^−2^). While the current densities at 0.6 V only have a decay of 2.5, 4.7, and 6.9% after 10k, 20k, and 30k cycles (Fig. 6f, right), further confirming the superior stability under DEFCs operation. In addition, the 1000 hours long-term stability of DEFC at 0.5 V was further performed, as shown in Fig. 6g. The current density retention was 92.1% after 1000 h stability test, demonstrating robust stability due to the interface synergism of Pd/Co@N-C. While the physical characteristics of the reacted anion-exchange membrane and the electrocatalytic activity of the reacted Pd/Co@N-C after 1000 h were well reserved (Supplementary Figs. 53 and 54), demonstrating that carbonates produced from EOR have a negligible poison effect on the membrane and catalyst (Supplementary Notes 7 and 8). The well-retained current density in low voltage implied that the MEA did not suffer severe mass transport loss or water flooding issues^67^, due to the unique structural effects and the synergistic interface between Pd and Co@N-C.

## Discussion

In summary, an ingenious structure design was proposed to engineer the synergistic interface of Pd/Co@N-C that can promote a direct C1-12e pathway for EOR in high-performance and long-lifetime DEFCs. Co promotes the transformation of amorphous carbon to highly graphitized carbon on Co NPs surfaces, which helps build up a unique structural effect between semi-embedded Pd and Co@N-C support. In addition, Pd donates electrons to N and results in an electron-deficient state of Pd in Pd/Co@N-C, which is important to boost EOR and ORR simultaneously. The spatial confinement effect at the interface of Co@N-C helps in the deagglomeration of the materials, preventing structural degradation of the catalysts during the operation of DEFCs. The strong catalyst-support effect of Pd/Co@N-C strengthens the electronic effect that enhances both the integrity/stability and activity/selectivity of the catalysts. The electronic effect at the interface of Pd/Co@N-C also enhances the electron transfer towards the direct C1-12e EOR pathway for DEFCs. The proposed Pd/Co@N-C with interface synergism and overall structural effects eventually delivers a maximum power density of 439 mW cm^−2^ in DEFCs and more than 1000 h of operation, which far surpasses the state-of-the-art catalysts. This work opens an alternative for the rational design of high-efficiency catalysts for DEFCs based on the concept of the synergistic interface, which will accelerate the development of a global economy powered by clean and sustainable catalysis and energy-related technology.

## Methods

### Materials

Unless mentioned otherwise, Fisher Scientific was the source of all chemicals acquired with analytical-grade purity. The solutions were made using ultrapure water with a conductivity of 18.2 MΩ cm^−1^. The baseline catalyst employed was commercial Pd/C, consisting of 10% (by weight) of 8-nm Pd nanoparticles on activated carbon, sourced from Sigma-Aldrich. It should be noted that due to the metal loadings and particle sizes of Pd/C being different from different vendors, the discrepancy is inevitable. To obtain repeatable data and to minimize the error ranges, the reported activities of all catalysts were tested at least three times. Fuel Cell Earth provided the Nafion solution (5.0 wt%), while the carbon paper (TGP-H-060) and anion-exchange membrane (Fumasep FAS-30) were procured from Fuel Cell Store.

### Synthesis of ZIF-67 and ZIF-8

The ZIF-67 was synthesized as follows: Co(NO_3_)_2_·6H_2_O (20 mmol) was dissolved in methanol (MeOH, 100 mL) to form clear solutions. 100 mL of MeOH containing 40 mmol of 2-methylimidazole (MeIm) was added to the solution of Co(NO_3_)_2_·6H_2_O and stirred for 24 h at room temperature. The purple precipitates were then centrifuged (8000 *g*, 5 min), washed with plenty of MeOH, and vacuum dried at 60 °C to obtain ZIF-67. The ZIF-8 was prepared by the same method using Zn(NO_3_)_2_·6H_2_O to replace Co(NO_3_)_2_·6H_2_O.

### Synthesis of ZIF-67@ZIF-8 core-shell structure

The as-synthesized ZIF-67 (1.0 g) was used as seeds and dispersed in 80 mL of MeOH solution. A total of 20 mmol Zn(NO_3_)_2_·6H_2_O solution in 100 mL MeOH was quickly added to the ZIF-67 solution, afterwards, the previous mixture was subjected to rigorous stirring at room temperature for 24 h while adding 80 mmol of 2-MeIm dissolved in 100 mL of MeOH. The light purple precipitates were centrifuged (8000 *g*, 5 min), washed with MeOH, and dried at 60 °C in a vacuum oven to obtain ZIF-67@ZIF-8. In addition, different amount of Zn(NO_3_)_2_·6H_2_O was used to obtain the optimized shell thickness.

### Synthesis of Co NPs coated with nitrogen-doped highly graphitic carbon (Co@N-C)

After synthesizing, the ZIF-67@ZIF-8 underwent pyrolysis at a heating rate of 5 °C min^−1^ under an Ar atmosphere for 2 hours at 950 °C. Once cooled to room temperature, the samples were washed with 0.5 M HCl solution under vigorous stirring overnight to remove the residual Zn and naked Co NPs. The next steps involved rinsing with ultrapure water and ethanol, and subsequently vacuum drying at 60 °C for the entire night, the Co@N-C was obtained. The N-C was prepared using the same method by the pyrolysis of ZIF-8. While the Co-N-C that the Co NPs without graphitic carbon layer coating was obtained by the pyrolysis of ZIF-67.

### Synthesis of Co NPs coated with carbon shell (Co@C)

Co(NO_3_)_2_·6H_2_O and urea with a molar ratio of 1:100 were dissolved in H_2_O and ultrasonicated for 1 h. Then the solution was stirred and evaporated at 80 °C to obtain a mixed powder. The powder was annealed at 950 °C for 1 h under N_2_. Then, the Co@C can be obtained.

### Embedding ultrafine Pd NPs on Co@N-C (Pd/Co@N-C)

Pd/Co@N-C was synthesized using an in-situ microwave reduction method, with a Pd and Co loading of 5 wt% and 10 wt%, respectively. (See ICP results in Supplementary Table 2). In brief, To form a consistent mixture, 95 mg of Co@N-C was dispersed in 100 mL of EG with the use of ultrasound. Next, a 5 mL H_2_PdCl_4_ solution (1 mg_Pd_ mL^−1^, PdCl_2_ in 0.1 M HCl solution) was added to the mixture under careful stirring. The pH was adjusted to approximately 11.0 using 0.1 M NaOH solution. The mixture was then placed in a microwave oven at 700 W for 90 seconds and stirred for another 2 hours. Finally, the mixture was thoroughly washed with water and ethanol and vacuum-dried at 60 °C overnight to produce Pd/Co@N-C. The Pd/N-C, Pd/Co@C, and Pd/Co-N-C were obtained by the same method using the N-C, Co@C, and Co-N-C as support. The loading of Pd was ca. 5 wt% for all control samples, while the Co loading was ca. 10 wt% for all Co-containing control samples, which was determined by ICP-MS.

### Preparing the Pd/Co@N-C with a high degree of Pd oxidized species (O-Pd/Co@N-C)

The O-Pd/Co@N-C was obtained by annealing 50 mg of Pd/Co@N-C in a tube furnace under an atmosphere at 200 °C for 12 h.

Synthesis of physically mixed Pd+Co@N-C. Pd NPs (ca. 4.2 nm) were synthesized using an oleylamine-mediated method, briefly, 15 mg of Pd(acac)_2_ and 30 mg of ascorbic acid were mixed in 5 mL of oleylamine under Argon in a glass vial. The glass vial was sealed and heated in an oil bath at 150 °C for 6 h. After being washed with plenty of ethanol and cyclohexane, the Pd NPs were obtained and stored in ethanol with a 1 mg_Pd_ mL^−1^. 2 mL of the above solutions were then mixed with 38 mg of Co@N-C, after being sonicated for 1 hour and stirred for 12 h, the products were washed with ethanol and dried at 80 °C overnight under vacuum, upon undergoing an hour of sonication and 12 h of stirring, the resulting compounds were cleansed with ethanol and subjected to vacuum drying at 80 °C overnight. the Pd+Co@N-C with the loading of 5 wt% Pd (detected by ICP-MS) was obtained. Besides, the Pt+Co@N-C and Pt/Co@N-C were also synthesized with a Pt loading of ~5 wt.% through the same method to prepare Pd+Co@N-C and Pd/Co@N-C, except H_2_PtCl_6_ was used to replace PdCl_2_.

### Electrochemical characterizations

Using a glassy carbon rotating ring-disk electrode tip (0.2475 cm^2^ disk area and 0.1866 cm^2^ Pt ring area) and an electrode rotator (Pine Research), electrochemical measurements were conducted at a temperature of approximately 23 °C on a CHI 760E electrochemical workstation. The counter electrode used was a graphite rod, and the reference electrodes were a Hg/HgO (1 M KOH) electrode. To maintain consistency, all potentials were measured in reference to a reversible hydrogen electrode (RHE). Before conducting the electrochemical measurement, a Pt electrode was used to generate a polarization curve for both the hydrogen evolution reaction and hydrogen oxidation reaction. This was done to calibrate the Hg/HgO reference electrode. All potentials reported in three-electrode half-cell in this work are versus RHE without iR-compensation. All three-electrode electrochemical tests were performed under environmental conditions except special notes such as the electrolytes used for NMR and titration experiments.

To prepare a homogeneous catalyst ink, 5 mg of the as-synthesized Pd/Co@N-C catalyst was dispersed in a plastic vial (with a volume of 2 mL) containing a solution of Nafion/ethanol/water (40 µL/480 µL/480 µL) and subjected to sonication for 1 h. The catalyst ink was then dropped onto the surface of the polished RRDE and allowed to dry naturally in the air. The catalyst loading was 8 µg_Pd_ cm^−2^, which was kept constant for the counterparts, including commercial Pd/C, Pd/N-C, and Pd+Co@N-C. For the electrochemical EOR experiments, a scan rate of 50 mV s^−1^ was used under static conditions without rotation in an N_2_-saturated 1.0 M KOH solution containing 1.0 M EtOH. The electrochemical impedance spectra (EIS) were recorded at a frequency range of 0.01 Hz to 100 kHz with an amplitude of 5 mV. For the electrochemical ORR experiments, LSV tests were performed at a sweep rate of 5 mV s^−1^ with 1600 rpm without iR correction in an O_2_-saturated 0.1 M KOH solution. The kinetic current density was calculated using the following equation:

1 $${J}_{K}=\frac{J \,\ast \,{J}_{L}}{{J}_{L}-J}$$

The measured current density, *J*, is composed of both kinetic current density, *J*_*K*_, and diffusion-limiting current density, *J*_*L*_. The electron transfer number (*n*) and hydrogen peroxide (H_2_O_2_) yield are calculated based on the disk current (I_Disk_) and ring current (I_Ring_) using the following equation:

2 $${{n}}=4\,{I}_{{disk}}/({I}_{{disk}}+{I}_{{disk}}/{{{{{\rm{N}}}}}})$$

The current collection efficiency of the Pt ring is *N* = 0.37.

Before the EOR and ORR recording, at least 20 cycles of cyclic voltammograms (CVs) were performed at 50 mV s^−1^ in N_2_-saturated 0.1 M KOH or 1 M KOH solution, The catalyst surface was cleaned and stabilized under CVs until a steady-state current was achieved. The CO stripping experiments were conducted in 0.1 M KOH solution, and CO gas (N_2_-balanced 10% CO, Airgas Co.) was bubbled into the 0.1 M KOH solution and kept the working electrode potential at 0.1 V_RHE_ for 15 min. To ensure monolayer adsorption of CO on the electrode surface, ultra-high purity N_2_ was purged into the electrolyte for 30 minutes. This step was necessary to remove any residual CO in the KOH solution. CVs were then recorded at a scan rate of 20 mV s^−1^. The ECSA was determined by integrating the charge of CO stripping while taking into account a charge density of 420 μC cm^−2^ and subtracting the background charge. To study the electronic effect and the contributions of different N-C support, the Randles-Sevcik equation was used to calculate the ECSA (details can be found in Supplementary Note 2). The experiments were performed in an N_2_-saturated 5 mM K_3_[Fe(CN_6_)]/ K_4_[Fe(CN_6_)] solution containing 0.1 M KNO_3_ at different scan rates.

For all catalysts, the mass activity was obtained by normalizing the Pd loading from ICP-MS results. While the specific activity normalized the peak current (for EOR) or kinetic current (for ORR) to the corresponding ECSA calculated from CO stripping. To test the durability, EOR underwent accelerated tests involving 500 cycles of cycling between 0.2 V_RHE_ and 1.2 V_RHE_ at a rate of 100 mV s^−1^, and from 0.6 to 1.0 V_RHE_ at 100 mV s^−1^ for 15k and 30k cycles, respectively for ORR. The electrochemical performance was tested at least three times for each sample, and the means and standard deviations were obtained and subsequently reported.

### MEA fabrication and DEFCs test

Fumasep FAS-30, an anion-exchange membrane (AEM) with specific hydroxide conductivity of 3.0~7.0 mS cm^−1^, a thickness of 25~35 μm, and ion-exchange capacity of 1.6~2.0 mmol g^−1^ (Fuel Cell Store) was prepared for use in the membrane assembly electrode (MEA). It was immersed in 0.5 M NaCl and 1 M KOH for three and four days, respectively, to convert it to OH^-^. After rinsing, it was carefully stored in ultrapure water (18.2 MΩ cm) for later use. To create catalyst inks, a combination of Pd/Co@N-C catalysts, 5% Nafion solution, ethanol, and ultrapure water was utilized in a ratio of 20 mg to 100 μL, 4 mL, and 1 mL, respectively. After homogeneously mixing and ultrasound treatment for 1 hour, the inks were applied by spraying onto Toray-60 carbon paper that had been treated with a waterproof coating containing 0.4 mg cm^−2^ carbon powder and 40 wt% PTFE. The Pd loading for each sample was 0.1, 0.5, and 1.0 mg cm^−2^, respectively. The anode catalyst layer (ACL), AEM, and cathode catalyst layer (CCL) were compressed together at a pressure of 400 N cm^−2^ for 3 minutes at a temperature of 80 °C. The resulting MEA was placed between two stainless steel bi-polar end plates, which were embedded with graphite plates featuring 2 mm parallel channel flow fields. The anode was then supplied with a 1 M KOH + 2 M EtOH solution at a flow rate of 5 mL min^−1^, while the cathode was fed with high purity O_2_ (99.99%) with 100% relative humidity at a flow rate of 100 mL min^−1^ without any backpressure. The polarization plots and stability of the fuel cell were documented at a temperature of 60 °C utilizing conventional fuel cell testing stations (Arbin Instrument Corp.). The test conditions involved potentiostatic measures with voltages ranging from the open circuit potential to 0.2 V, and a sweep rate of 0.5 mV s^−1^. Control MEAs with Pd/C, Pd/N-C, and Pd+Co@N-C as both anode and cathode catalysts with the same Pd loading were also prepared and studied. After 1000 hours of stability testing, the Pd/Co@N-C MEA was disassembled. The reacted Pd/Co@N-C catalyst was used to test ECSA and EOR activity. While for the reacted AEM, the SEM, specific area resistance and conductivity were tested after washing the catalyst layer multiple times with ultrapure water.

### In-situ ATR-SEIRAS tests

The Pd/Co@N-C catalyst layer was deposited on a chemically deposited Au film on a Si ATR prism, and the ATR-SEIRAS measurements were performed using an Agilent Technologies Cary 660 FTIR spectrometer with an MCT detector. The spectral resolution was set to 4 cm^−1^. Alumina slurry with 0.05 µm was utilized to polish the Si prism, followed by rinsing with ultrapure water and acetone consecutively to eliminate alumina residue. Then the Au film on Si was prepared using an electroless chemical plating method. The plating solution containing 15 mM of HAuCl_4_, 50 mM of Na_2_S_2_O_3_·5H_2_O, 150 mM of Na_2_SO_3_, 50 mM of NH_4_Cl, and a 2% HF solution with a volume ratio of 2:1, the chemical plating was performed under 60 °C for 60 seconds. The Si prism and Au/Si prism have negligible activity and impact on the reported in-situ ATR-SEIRAS results, as attested in Supplementary Figs. 40, 41 and Supplementary Note 5. The Pd/Co@N-C ink was deposited onto the chemically deposited Au film via a pipette, and the loading of 0.02 mg_Pd_ cm^−2^ and/or 0.5 mg_Pd_ cm^−2^ was used to obtain the best spectroscopic signal results. A Solartron 1260/1287 system was utilized to regulate the potential in spectroscopic measurements. The reference electrode was a saturated Ag/AgCl, and the counter electrode was a graphite rod. Spectra were collected potentiostatically in the potential range of 0-1.4 V_RHE_ with 0.1 V intervals in an electrolyte containing 1 M EtOH and 0.1 M KOH. The spectrum collected at 0 V_RHE_ in 0.1 M KOH without ethanol was used as the reference spectrum.

### Materials characterizations

A transmission electron microscope (TEM) with energy-dispersive X-ray spectroscopy (EDS) mapping (Cs-corrected JEM ARM200F STEM and FEI Titan) was used to characterize the morphology and composition of the catalysts. The structural information was tested using X-ray diffraction (XRD) with Cu Kα radiation, performed by PANalytical B.V. The surface electron structure was investigated using X-ray photoelectron spectroscopy (XPS, ESCALAB Xi + ). Compositions of the materials were studied using a Raman microscope (Renishaw). An inductively coupled plasma mass spectrometer (ICP-MS, iCAP RQ, Thermo Scientific USA) was utilized to detect the metal contents. The electrical conductivity of N-C and Co@N-C was evaluated by Keithley 2400 four-probe conductivity meter. ^1^H-NMR and ^13^C-NMR were performed on Varian VNMRS-500 MHz NMR. The electrolytes were collected after the electrochemical tests at 0.4, 0.5, 0.6, and 0.7 V_RHE_ in the three-electrode system and 0.4 V in the fuel cell device for the NMR measurements. To determine the liquid products through ^1^H-NMR, 0.5 mL of the post-reaction solution was combined with 0.1 mL each of D_2_O and DMSO. The ^1^H-NMR spectrum was measured using a pre-saturation method to suppress water. The isotopically labeled measurement was further performed. The 1-^13^C labeled ethanol (CH_3_^13^CH_2_OH, >98%, purchased from Cambridge Isotope Laboratories, Inc.) was used to prepare the 1 M KOH + 1 M CH_3_^13^CH_2_OH. An H-type cell was used to evaluate the CV curves in a 1 M KOH + 1 M CH_3_^13^CH_2_OH solutions (12 mL) that was saturated with N_2_. The scan rate was 50 mV s^-1^, and the cell was sealed and free of air. Then EOR chronoamperometric test at 0.5, 0.7, 0.9, and 1.1 V_RHE_ was performed, respectively. After 12 hours of the *i-t* test, the post-reaction solutions (0.5 mL) at each potential were collected, and mixed with 0.2 mL D_2_O to identify the ^13^CO_3_^2-^ and CH_3_^13^COO^−^ products by the ^13^C-NMR immediately.

### Calculation of faradic efficiency (FE)

The calculation of carbon balance, from ethanol to CO_2_ or acetate, was performed using two pathways that produce either CO_2_ or acetate in an alkaline solution, respectively.

3 $$12{{{{{\rm{e}}}}}}\,{{{{{\rm{pathway}}}}}}:\,{{{{{{\rm{CH}}}}}}}_{3}{{{{{{\rm{CH}}}}}}}_{2}{{{{{\rm{OH}}}}}}+16{{{{{{\rm{OH}}}}}}}^{-}\to 2{{{{{{\rm{CO}}}}}}}_{3}^{{2}_{-}}+11{{{{{{\rm{H}}}}}}}_{2}{{{{{\rm{O}}}}}}+12{e}^{-}$$

4 $$4{{{{{\rm{e}}}}}}\,{{{{{\rm{pathway}}}}}}:\,{{{{{{\rm{CH}}}}}}}_{3}{{{{{{\rm{CH}}}}}}}_{2}{{{{{\rm{OH}}}}}}+5{{{{{{\rm{OH}}}}}}}^{-}\to {{{{{{\rm{CH}}}}}}}_{3}{{{{{{\rm{COO}}}}}}}^{-}+4{{{{{{\rm{H}}}}}}}_{2}{{{{{\rm{O}}}}}}+4{e}^{-}$$

Following the i-*t* test conducted at 0.4, 0.5, 0.6, and 0.7 V_RHE_ for 3 hours, 30 mL of the resulting electrolyte was utilized for titration. The titration involved the addition of an excessive amount of barium hydroxide (Ba(OH)_2_·8H_2_O, 1 M, Acros, 98%). A sealed and air-free H-type cell was utilized for titration to quantify CO_3_^2-^, with a continuous flow of N_2_ gas to avoid CO_2_ contamination from the air. The BaCO_3_ precipitation generated during the titration was collected, washed, and dried to determine the amount of CO_3_^2-^.

A single fuel cell was evaluated for the production of CO_3_^2-^ and acetate resulting from the oxidation of ethanol, using the same method. The fuel cell is operated in a glove box to avoid contaminating CO_2_ in the atmosphere.

The calculation of the EOR’s FE towards CO_2_ or acetate was carried out in the following manner:

5 $${FE}\left[\%\right]=\frac{{zNF}}{q} \,*\, 100$$

Where z is the theoretical number of electrons needed to produce the desired product, while N indicates the number of moles generated. F is the Faradaic constant (96485 C mol^−1^), and q is the overall charge administered during the process, which is also taken into account.

### Computational methods

Using the Vienna ab initio simulation package (VASP)^68,69^ software, we conducted first principles DFT^70,71^ calculations with a plane-wave basis set. The Perdew, Burke and Ernzernhof (PBE) functionals of generalized gradient approximation (GGA)^72^ were used to describe the electronic exchange and correlation energy, while a cutoff energy of 500 eV was set for the plane wave expansion. To account for core electrons, a projector augmented wave (PAW)^73,74^ pseudopotential was utilized. The atomic positions were relaxed until the force on each ion fell below 0.01 eV Å^−1^, and the electronic energy was converged until the energy difference between iterations was lower than 10^-6 ^eV. The adsorption energy calculations for the Pd_13_ cluster involved modeling the carbon support with nitrogen doping. This was achieved by replacing seven carbon atoms with three nitrogen atoms in the p(7 × 7) graphene layer. To study the impact of OH coverage on ORR performance, we created a Pd(111) substrate comprising four Pd layers, with the uppermost two undergoing relaxation during structural optimization. The p(2 × 2), p(3 × 3), and p(4 × 4) Pd(111) surfaces covered by OH were utilized to model the Pd(111) surface with an OH coverage of 1/4, 1/9, and 1/16, respectively. To reduce the interaction between periodic images, a vacuum layer with a thickness of 20 Å was applied perpendicularly to the surface. For Pd_13_ cluster adsorption calculations, a gamma-centered^75^ scheme with 2 × 2 × 1 k-point mesh was used to sample the Brillouin Zone. For p(4 × 4), p(3 × 3), and p(2 × 2) Pd(111) surfaces, a 3 × 3 × 1, 5 × 5 × 1, and 6 × 6 × 1 k-point mesh were used, respectively. The calculation of all adsorbate systems incorporated the Zero-point energy (ZPE) corrections, expressed by ${{{{{\rm{ZPE}}}}}}=\mathop{\sum}\limits_{i}\frac{1}{2}h{\nu }_{i}$. Here, *h* represents Planck’s constant and *v*_*i*_ indicates the frequency of the ith vibrational mode of the binding molecules.

## Supplementary information

Supplementary Information

## Data Availability

The data that support the findings of this work are available within the article and its Supplementary Information file. The source data are available from the corresponding author upon reasonable request.
