# Supplementary material for: Interface synergism and engineering of Pd/Co@N-C for direct ethanol fuel cells
Source: Nat Commun. 2023 Mar 11;14:1346. doi: 10.1038/s41467-023-37011-z (PMC10008627; doi:10.1038/s41467-023-37011-z)
Supplement: Supplementary file 1 — Supplementary Information [file 41467_2023_37011_MOESM1_ESM.pdf]

Supplementary information for

Interface Synergism and Engineering of Pd/Co@N-C for Direct Ethanol Fuel Cells

Jinfa Chang[1,10], Guanzhi Wang[1,2,10], Xiaoxia Chang[3], Zhenzhong Yang[4], Han Wang[4], Boyang Li[5], Wei Zhang[1,2], Libor Kovarik[4], Yingge Du[4], Nina Orlovskaya[6,7], Bingjun Xu[3], Guofeng Wang[5], Yang Yang[1,2,7,8,9*]

1. NanoScience Technology Center, University of Central Florida, Orlando, FL 32826, USA
2. Department of Materials Science and Engineering, University of Central Florida, Orlando, FL 32826, USA
3. Catalysis Center for Energy Innovation, Department of Chemical and Biomolecular Engineering, University of Delaware, Newark, Delaware 19716, USA
4. Physical and Computational Sciences Directorate, Pacific Northwest National Laboratory, Richland, WA 99352, USA
5. Department of Mechanical Engineering and Materials Science, University of Pittsburgh, Pittsburgh, PA 15261, USA.
6. Department of Mechanical and Aerospace Engineering, University of Central Florida, Orlando, FL 32816, USA
7. Renewable Energy and Chemical Transformation Cluster, University of Central Florida, Orlando, FL 32816, USA
8. Department of Chemistry, University of Central Florida, Orlando, FL 32816, USA
9. The Stephen W. Hawking Center for Microgravity Research and Education, University of Central Florida, Orlando, FL 32826, USA
10. These authors contributed equally.

*Correspondence and requests for materials should be addressed to Y.Y. (Email: Yang.Yang@ucf.edu)

Contents:

Supplementary Figures 1-54

Supplementary Tables 1-8

Supplementary Notes 1-8

Supplementary Figures

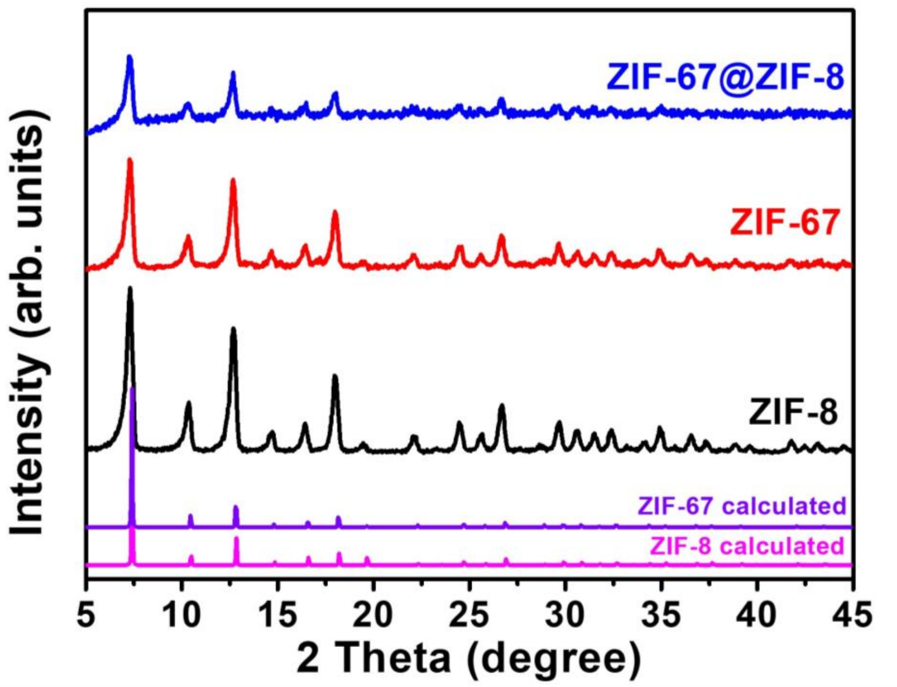

Supplementary Fig. 1. XRD patterns of ZIF-67@ZIF-8, ZIF-67, and ZIF-8.

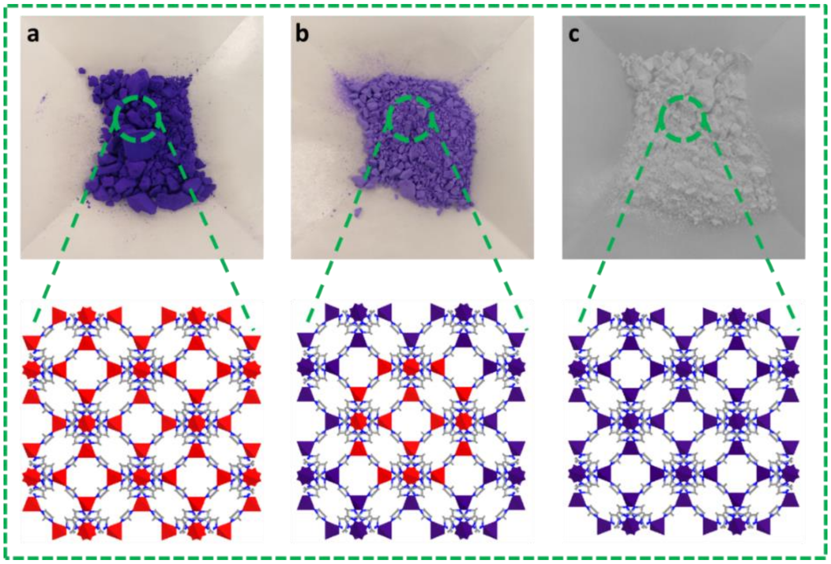

Supplementary Fig. 2. Optical photographs of samples. (a) ZIF-67, **(b)** ZIF-67@ZIF-8, and **(c)** ZIF-8. The color changes of ZIF-67@ZIF-8 from ZIF-67 and ZIF-8 indicate the successful synthesis of ZIF-67@ZIF-8.

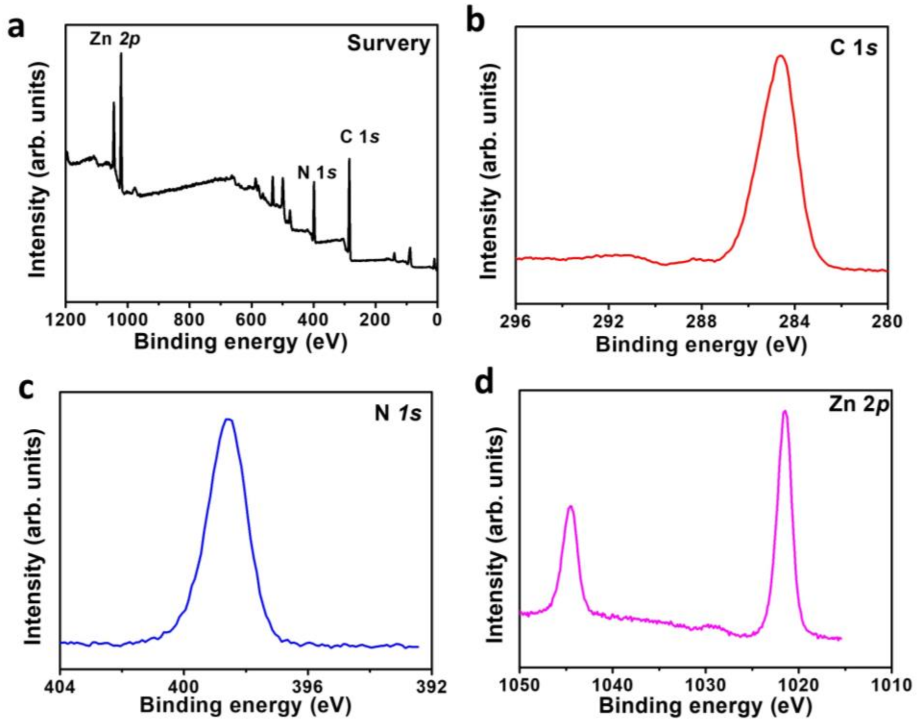

Supplementary Fig. 3. XPS spectra of ZIF-8. (a) survey, **(b)** C 1*s*, **(c)** N 1*s*, and **(d)** Zn 2*p*.

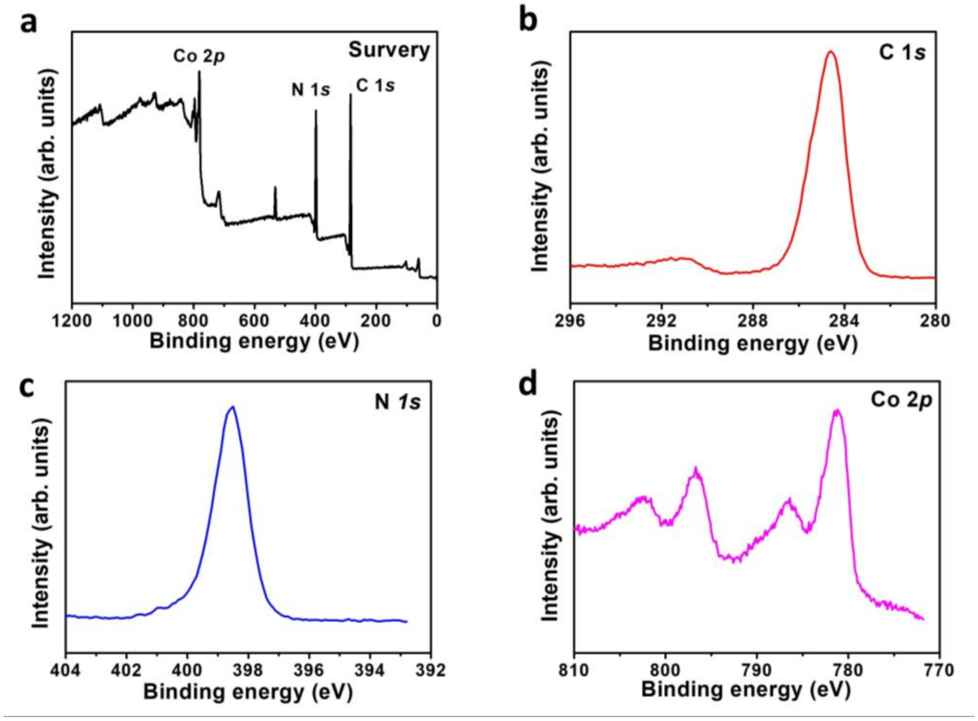

Supplementary Fig. 4. XPS spectra of ZIF-67. (a) survey, **(b)** C 1*s*, **(c)** N 1*s*, and **(d)** Co 2*p*.

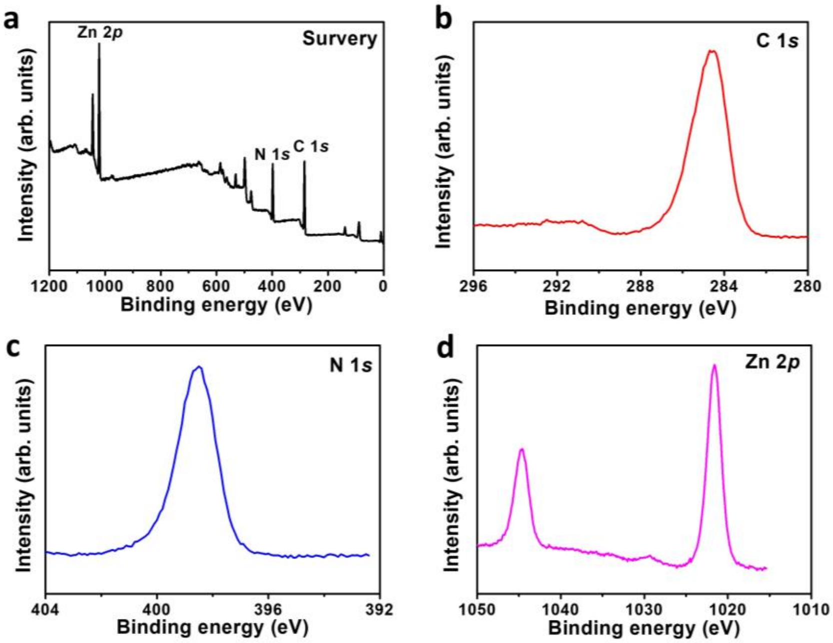

Supplementary Fig. 5. XPS spectra of ZIF-67@ZIF-8. (a) survey, **(b)** C 1*s*, **(c)** N 1*s*, and **(d)** Zn 2*p*. The disappearance of the Co signal indicates the surface coating of ZIF-67 by ZIF-8.

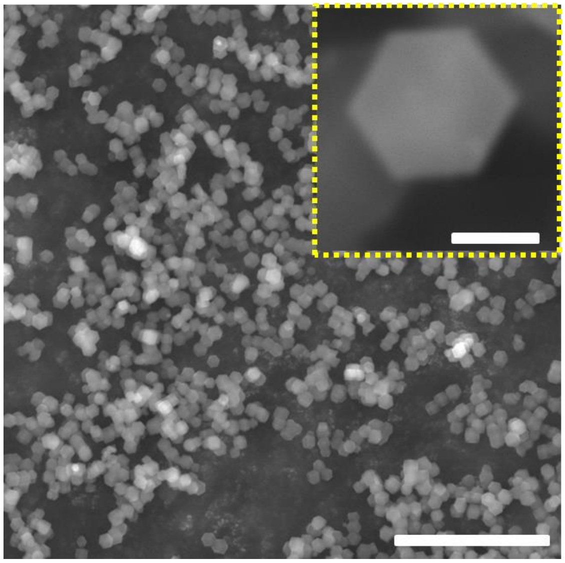

Supplementary Fig. 6. SEM image of ZIF-67@ZIF-8. Scale bar: 10 μm. The inset shows a high-magnification SEM image (scale bar: 500 nm).

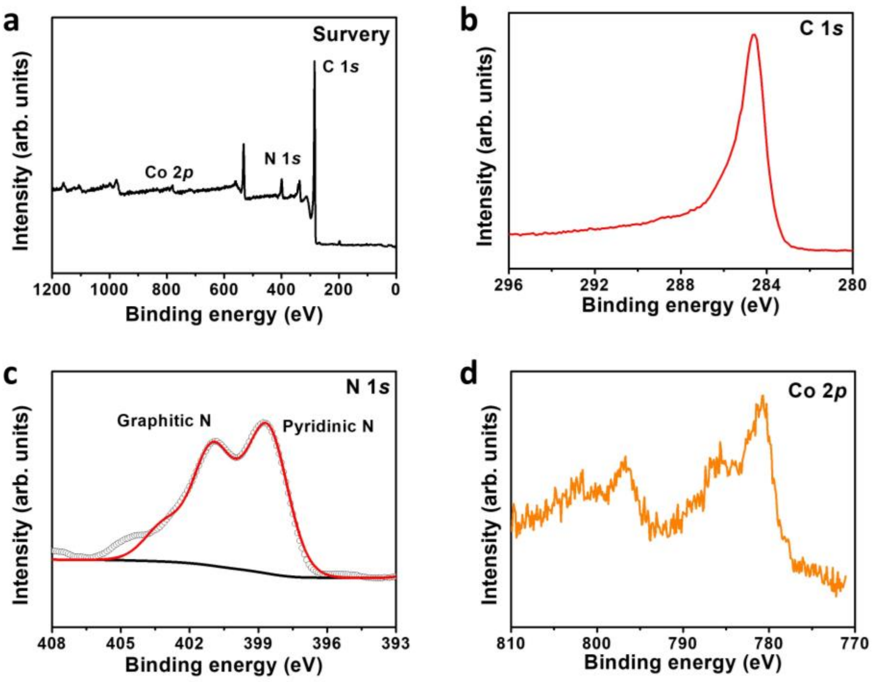

Supplementary Fig. 7. XPS spectra of Co@N-C. (a) survey, **(b)** C 1*s*, **(c)** N 1*s*, and **(d)** Co 2*p*.

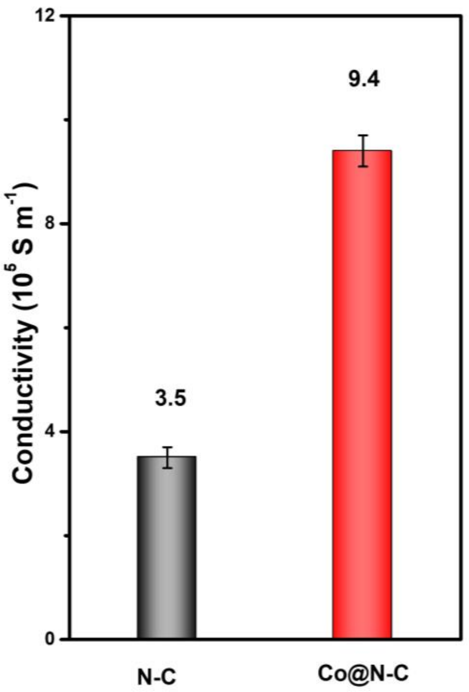

Supplementary Fig. 8. Electrical conductivity of N-C and Co@N-C. The error bars represent the standard deviations (s.d.) of at least three independent measurements, and the data were presented as mean values ± s.d.

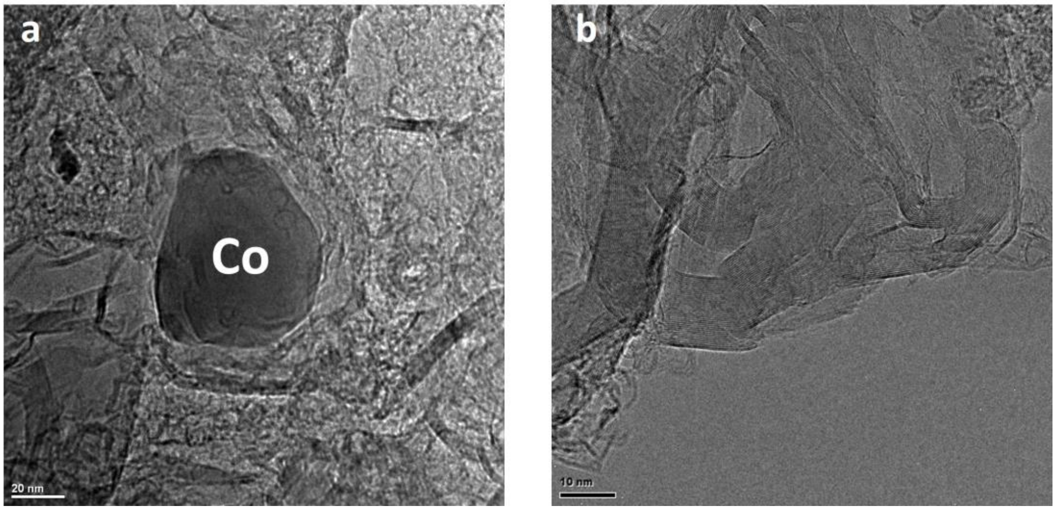

Supplementary Fig. 9. TEM images of Co-N-C. (a) before and (**b)** after acid washing. The Co-N-C was obtained from direct pyrolysis of ZIF-67 at 950 ^{o}C for 3 hours. It can be seen from **(a)** that the Co NPs were supported on N-C without graphitic layers coating. After being washed with 1 M HCl solution, the Co NPs were leached out of N-C. The scale bar in **(a)** and **(b)** is 20 and 10 nm, respectively.

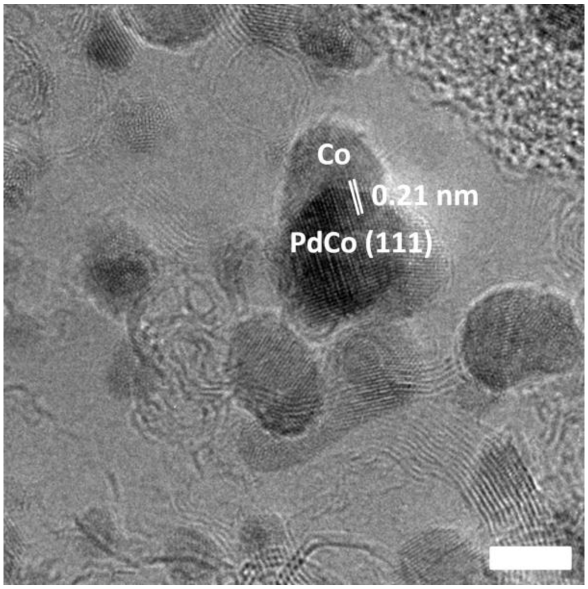

Supplementary Fig. 10. TEM image of Pd supported on Co-N-C (Pd/Co-N-C). Without the protection of N-C on the surface of Co NPs, the galvanic replacement reaction ($Co + Pd^{2+} \rightarrow Co^{2+} + Pd$) happened when the Co-N-C (without HCl washing) was used to support Pd. And PdCo alloy was formed due to the lack of spatial confinement effect. The scale bar is 5 nm.

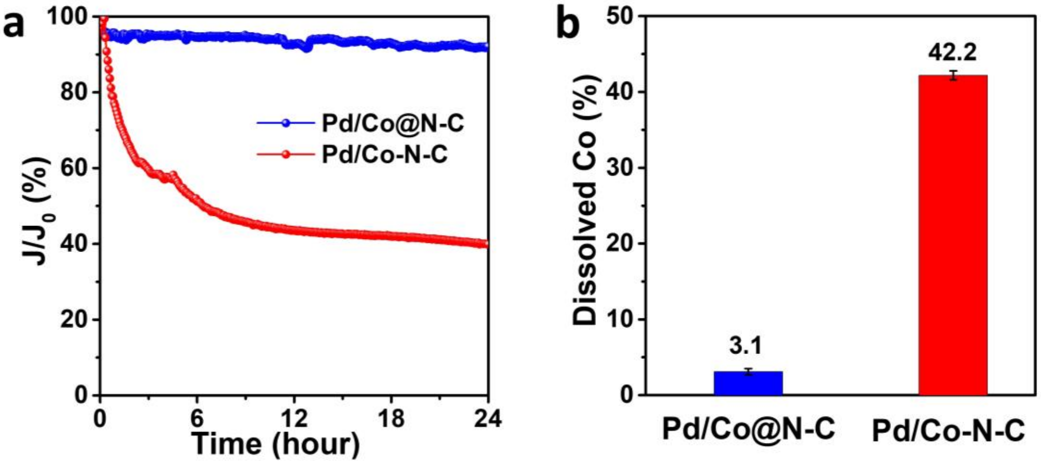

Supplementary Fig. 11. The stability of catalyst with/without spatial confinement effect. (a) ORR stability of Pd/Co@N-C and Pd/Co-N-C with/without spatial confinement effect at 0.6 V_{RHE}. ORR rather than EOR was selected because the CO poisoning species will be accumulated on the catalyst surface during the EOR stability test. **(b)** The concentration of Co in electrolyte after 24 hours of ORR stability test.

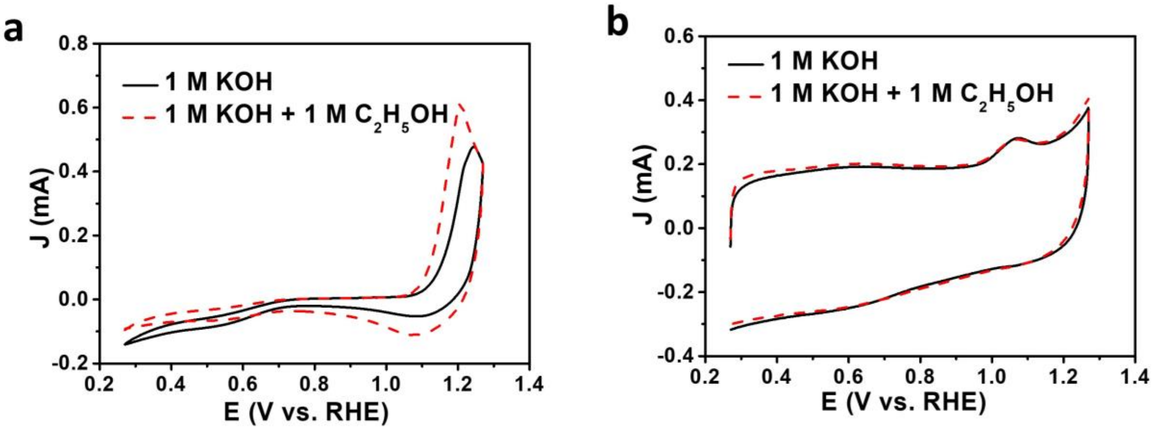

Supplementary Fig. 12. EOR performance of ZIF-67@ZIF-8 and Co@N-C. CVs curves of **(a)** ZIF-67@ZIF-8 and **(b)** Co@N-C in Ar-saturated 1.0 M KOH with and without 1.0 M EtOH aqueous solution at a scan rate of 50 mV s^{-1}. No EOR activity was found in both samples, indicating that the EOR activity of Pd/Co@N-C is from Pd, while the contribution from Zn is merely negligible for EOR.

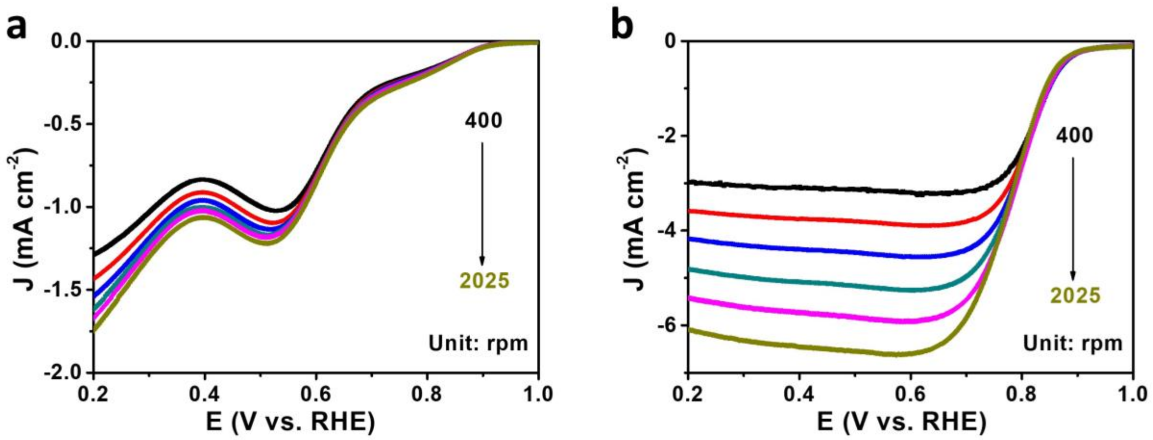

Supplementary Fig. 13. ORR performance of ZIF-67@ZIF-8 and Co@N-C. LSV curves of **(a)** ZIF-67@ZIF-8 and **(b)** Co@N-C in O_2-saturated 0.1 M KOH aqueous solution with 5 mV s^{-1} at various rotating speeds (400, 625, 900, 1225, 1600, and 2025 rpm). A poor ORR activity was found on the ZIF-67@ZIF-8 and Co@N-C, indicating that the ORR activity of Pd/Co@N-C comes from Pd, while the contribution from Zn is merely negligible for ORR.

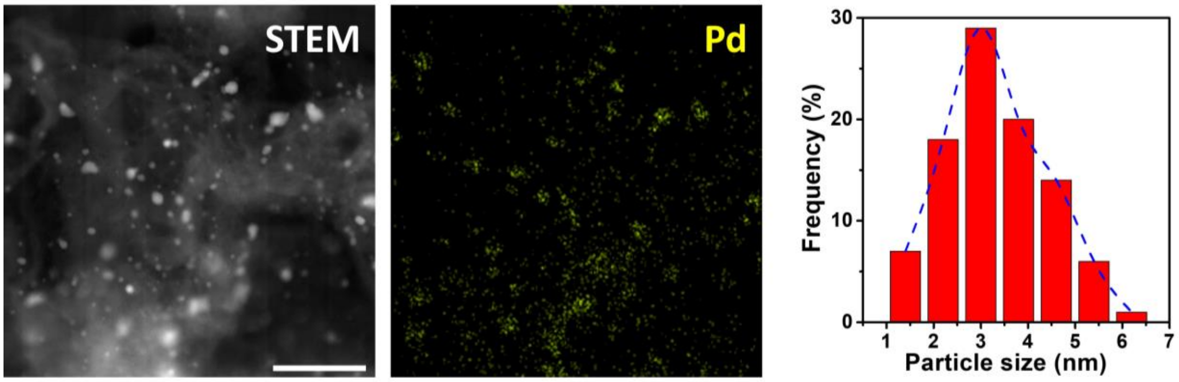

Supplementary Fig. 14. STEM and statistic estimation of the size of Pd in Pd/Co@N-C by checking 100 particles in the sample. Scale bar: 50 nm.

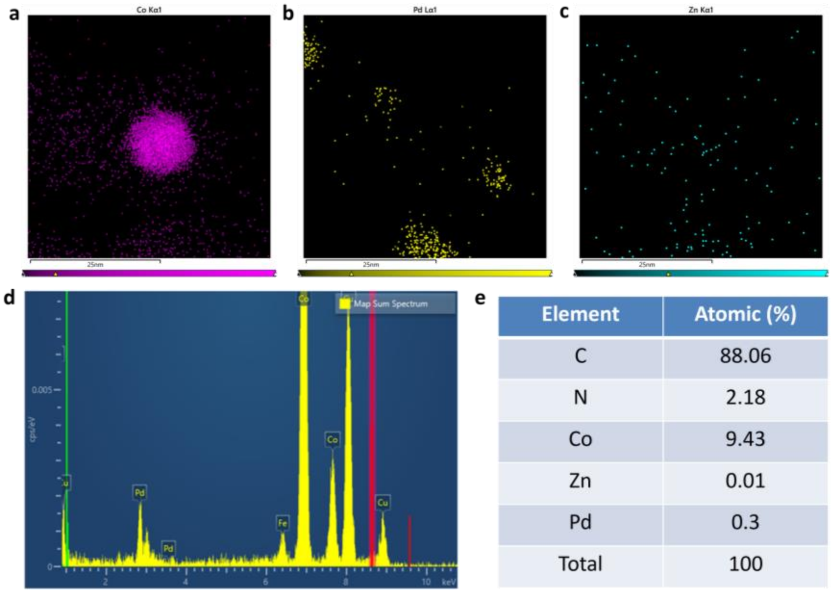

Element	Atomic (%)
C	88.06
N	2.18
Co	9.43
Zn	0.01
Pd	0.3
Total	100

Supplementary Fig. 15. EDS elemental mappings of Pd/Co@N-C. (a) Co, **(b)** Pd, and **(c)** Zn. **(d)** EDX spectrum and **(e)** corresponding elemental contents of Pd/Co@N-C. The red line in **(d)** marks the peak position of Zn, showing that the Zn content is below the detection limit. The Cu signal is from the Cu substrate used for TEM tests. The Fe signal is from the TEM instrument.

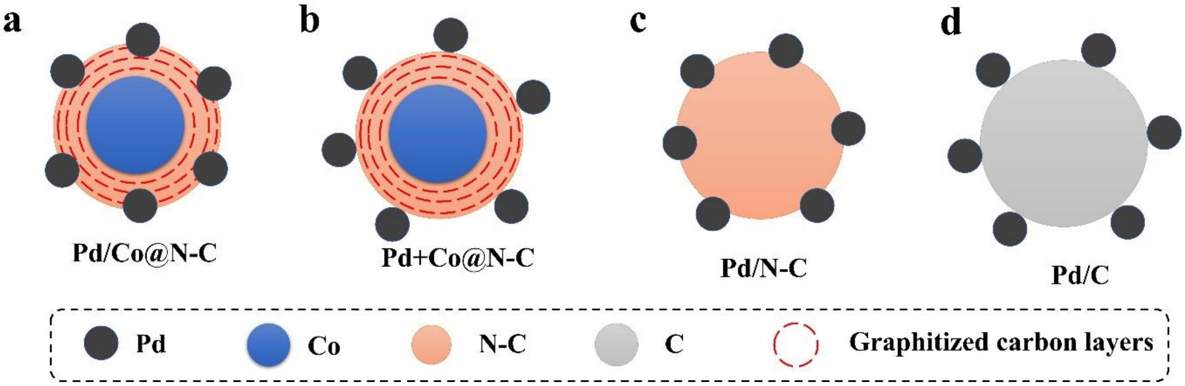

Supplementary Fig. 16. Interface synergism of Pd/Co@N-C. The proposed interface configuration of **(a)** Pd/Co@N-C, **(b)** Pd+Co@N-C, **(c)** Pd/N-C, and **(d)** Pd/C catalysts.

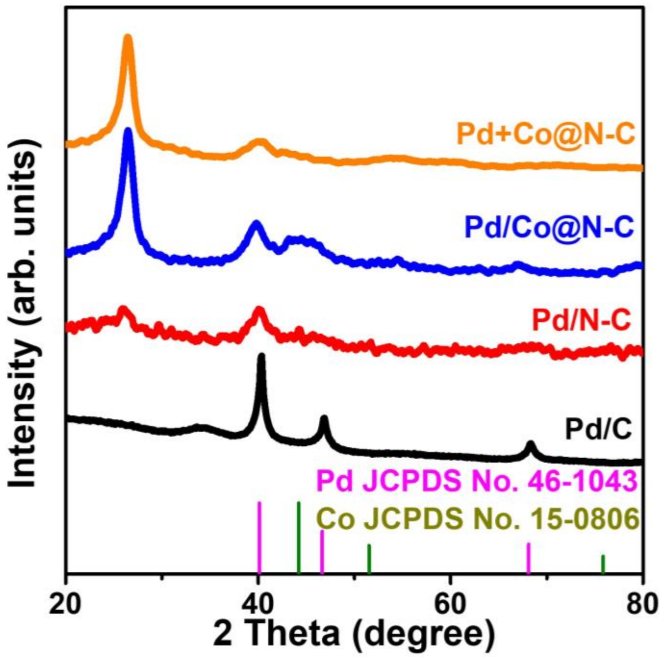

Supplementary Fig. 17. XRD patterns of different samples.

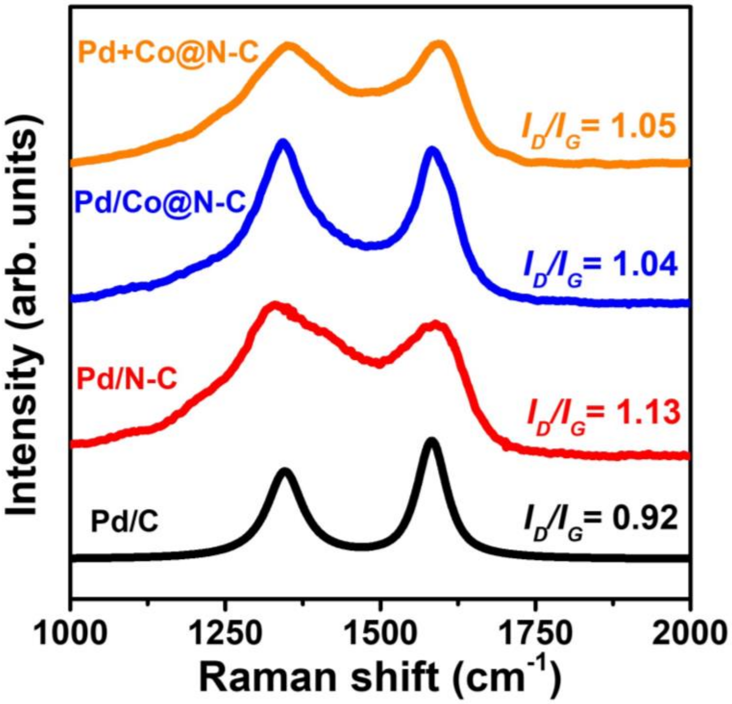

Supplementary Fig. 18. Raman patterns of different samples.

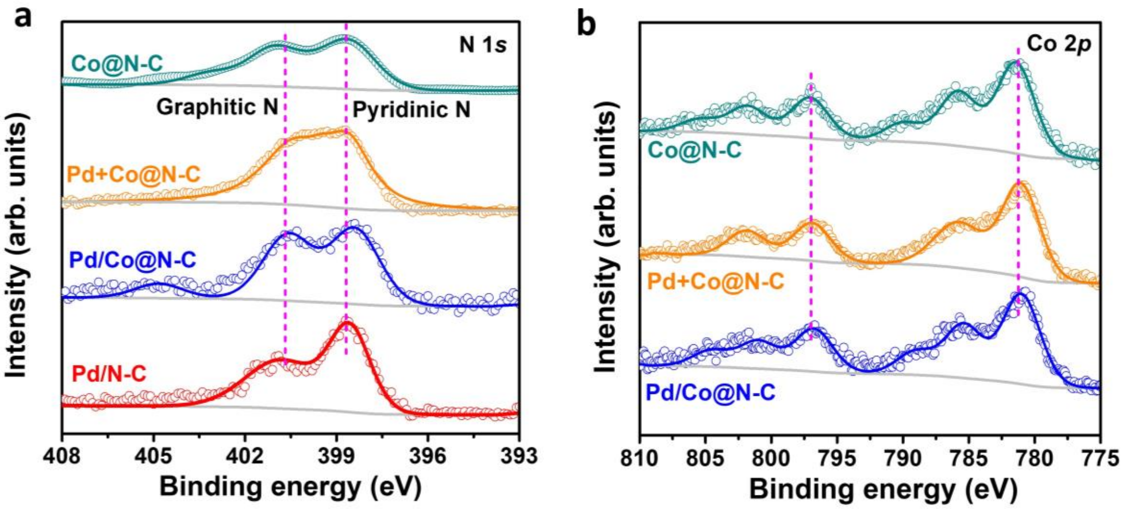

Supplementary Fig. 19. XPS spectra. (a) N 1*s* peaks of Pd/N-C, Pd/Co@N-C, Pd+Co@N-C, and Co@N-C. **(b)** Co 2*p* peaks of Pd/Co@N-C, Pd+Co@N-C, and Co@N-C. The XPS N 1*s* peaks of Pd/Co@N-C shift to lower binding energy (~0.4 eV) compared to Pd/N-C, Pd+Co@N-C, and Co@N-C support, indicating the strong electronic effect between Pd and N. While no obvious shift can be found on the Co 2*p* peaks. The graphitic-N content in Co@N-C, Pd+Co@N-C, Pd/Co@N-C, and Pd/N-C samples is 48.5%, 47.2%, 48.1%, and 36.9%, respectively.

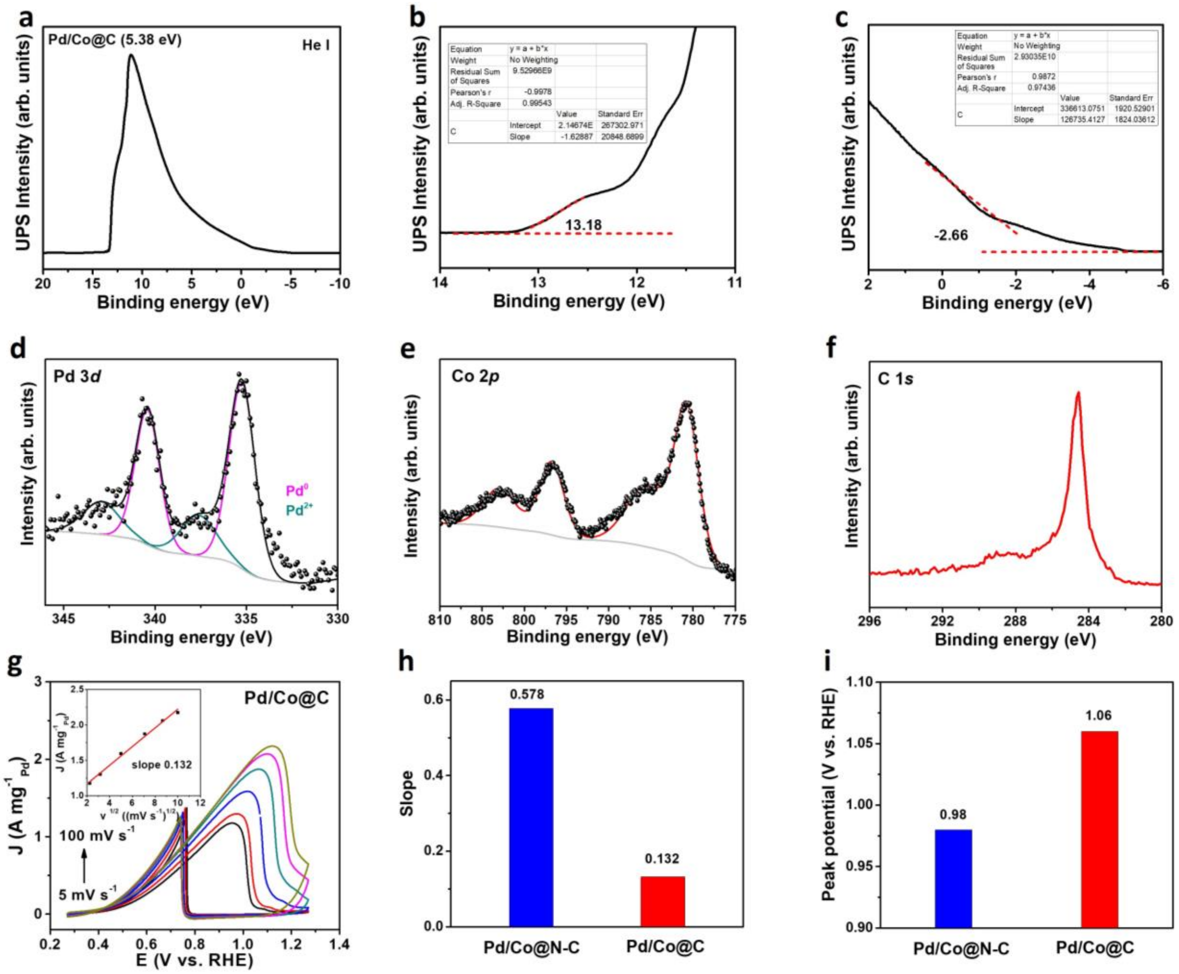

Supplementary Fig. 20. XPS, UPS, and EOR performance of Pd/Co@C. (a) the tested UPS spectra, (**b-c**) the corresponding enlarged areas to determine the values of $E_{cuf\text{-}off}$ and VBM. XPS **(d)** Pd 3*d*, **(e)** Co 2*p*, and **(f)** C 1*s* spectra of Pd/Co@C. **(g)** CVs curves of EOR on Pd/Co@C catalyst in 1.0 M KOH + 1.0 M EtOH aqueous solution at different scan rates (5, 10, 25, 50, 75, and 100 mV s^{-1}). The inset is the *J* as a function of the square root of the scan rate ($v^{1/2}$). Comparison of **(h)** slope and **(i)** EOR peak potential on Pd/Co@N-C and Pd/Co@C obtained from (g).

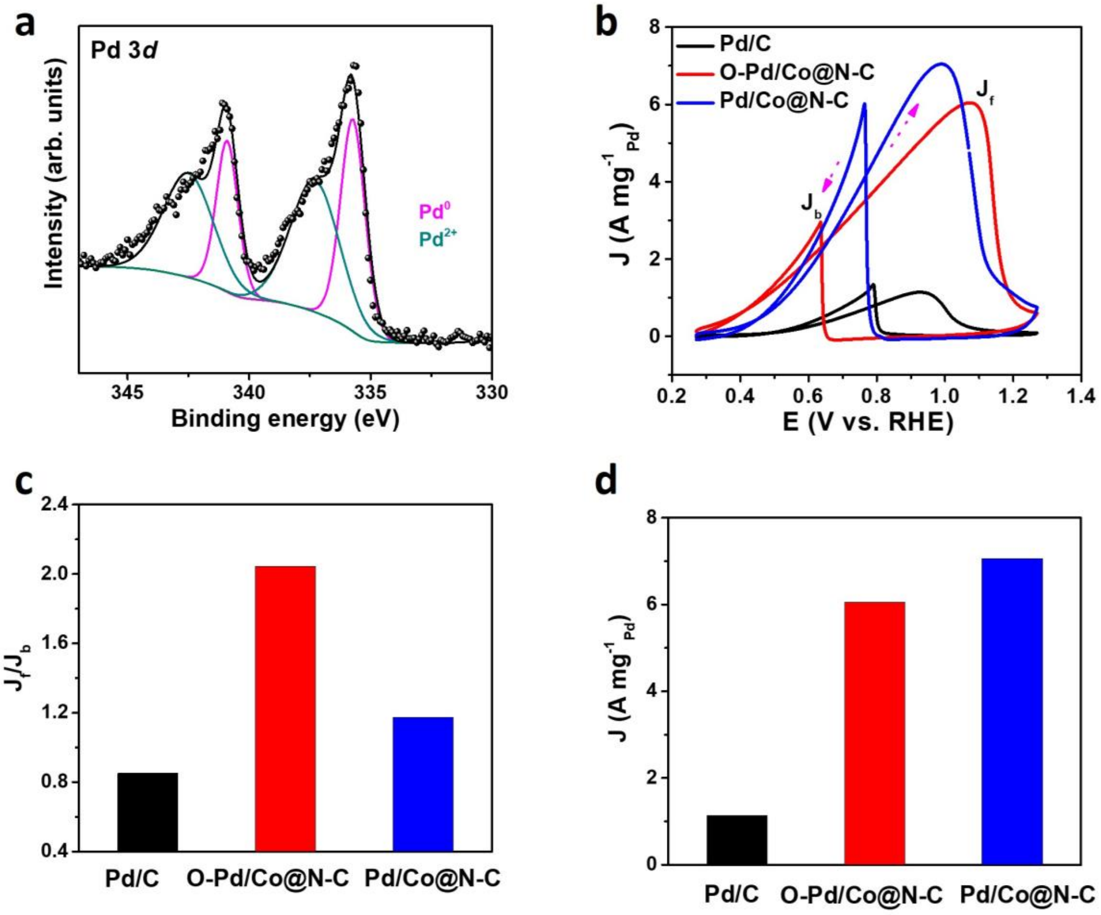

Supplementary Fig. 21. EOR performance of O-Pd/Co@N-C with a high oxidation degree. **(a)** XPS Pd 3*d* spectra of O-Pd/Co@N-C. The contents of Pd^0 and Pd^{2+} are 42.2% and 57.8% in O-Pd/Co@N-C. **(b)** CV curves of different catalysts in the N_2-saturated 1 M KOH + 1 M EtOH at a scan rate of 50 mV s^{-1}. The dashed arrows indicate the scan direction. **(c)** The J_f/J_b value and **(d)** the peak current density of Pd/C, O-Pd/Co@N-C, and Pd/Co@N-C.

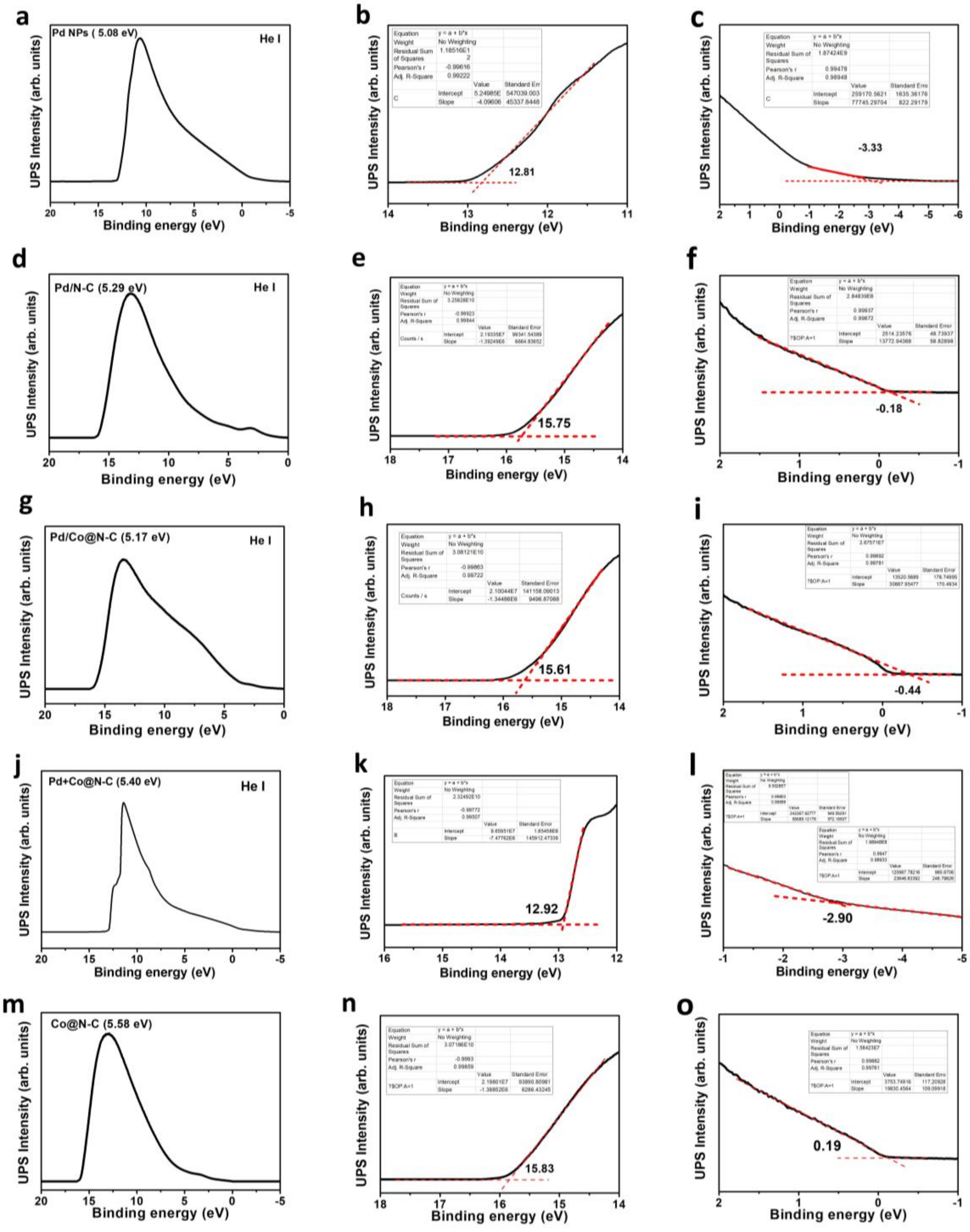

Supplementary Fig. 22. UPS spectra of **(a-c)** Pd NPs, **(d-f)** Pd/N-C, **(g-i)** Pd/Co@N-C, **(j-l)** Pd+Co@N-C, and **(m-o)** Co@N-C. The (**a**, **d**, **g**, **j, m**) were the tested UPS spectra. The (**b**, **e**, **h**, **k, n**) and (**c**, **f**, **i**, **l, o**) were the corresponding enlarged areas to determine the values of $E_{cuf\text{-}off}$ and valence band maximum (VBM). The photo energy $E_{He\text{-}I}$ = 21.22 eV. The work function (WF, ϕ) is calculated as $\phi = h\nu - (E_{cut\text{-}off} - VBM)$.

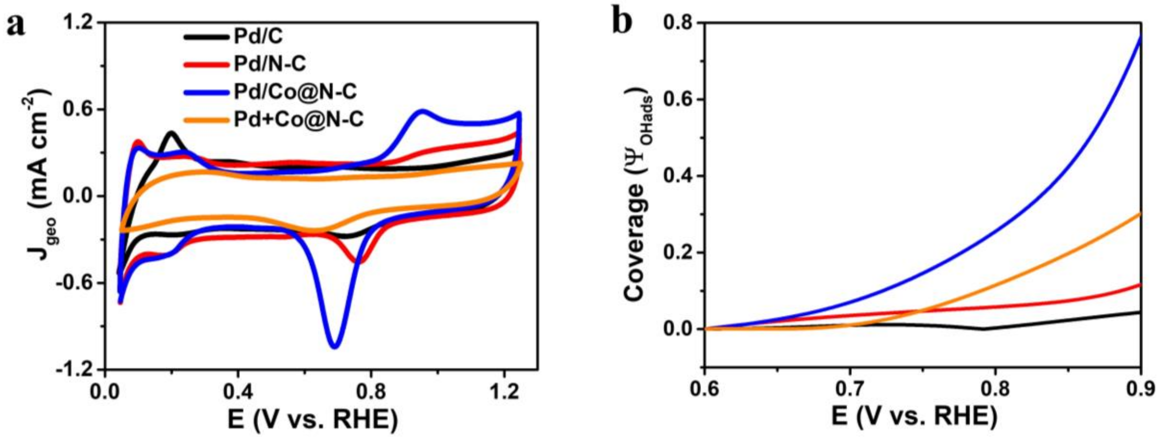

Supplementary Fig. 23. Comparison of the O/OH$_{ads}$ adsorption ability as a function of potential for Pd/C, Pd/N-C, Pd/Co@N-C, and Pd+Co@N-C. (a) Cyclic voltammograms (CVs) of freshly prepared samples in N_2-saturated 0.1 M KOH solutions at a scan rate of 50 mV s^{-1}. **(b)** Comparison of O/OH$_{ads}$ coverage as a function of potential for different samples.

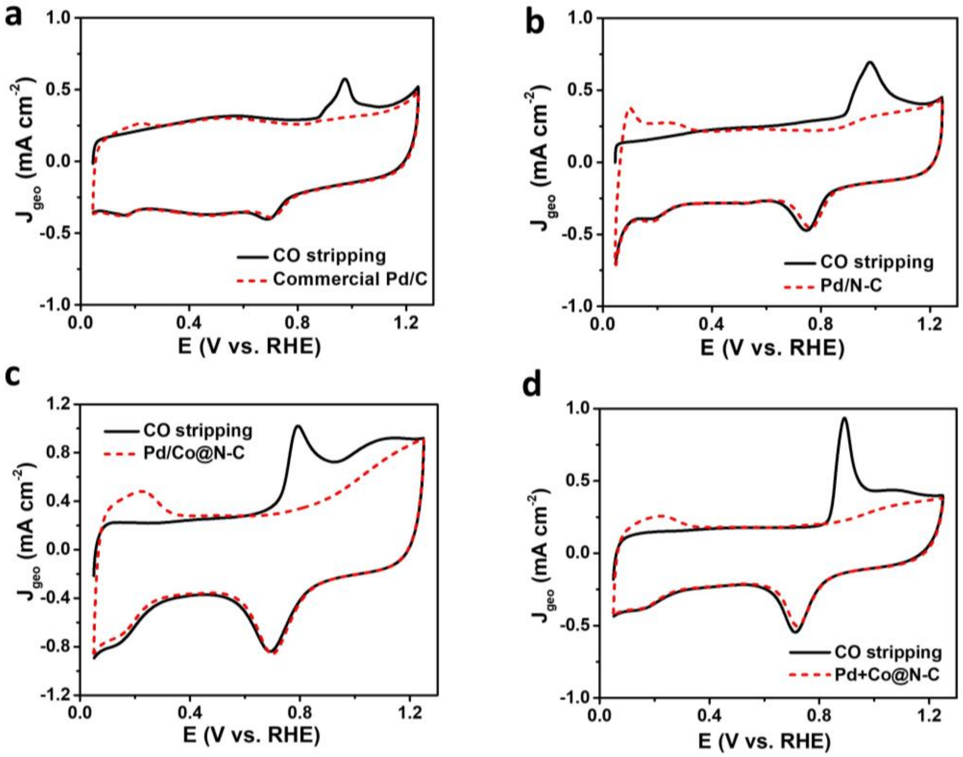

Supplementary Fig. 24. CO stripping CV curves. (a) Pd/C, **(b)** Pd/N-C, **(c)** Pd/Co@N-C, and **(d)** Pd+Co@N-C catalysts. The CV curves were conducted in 0.1 M KOH at a scan rate of 20 mV s^{-1}. The ECSA of each catalyst was calculated from the charge integration of CO stripping.

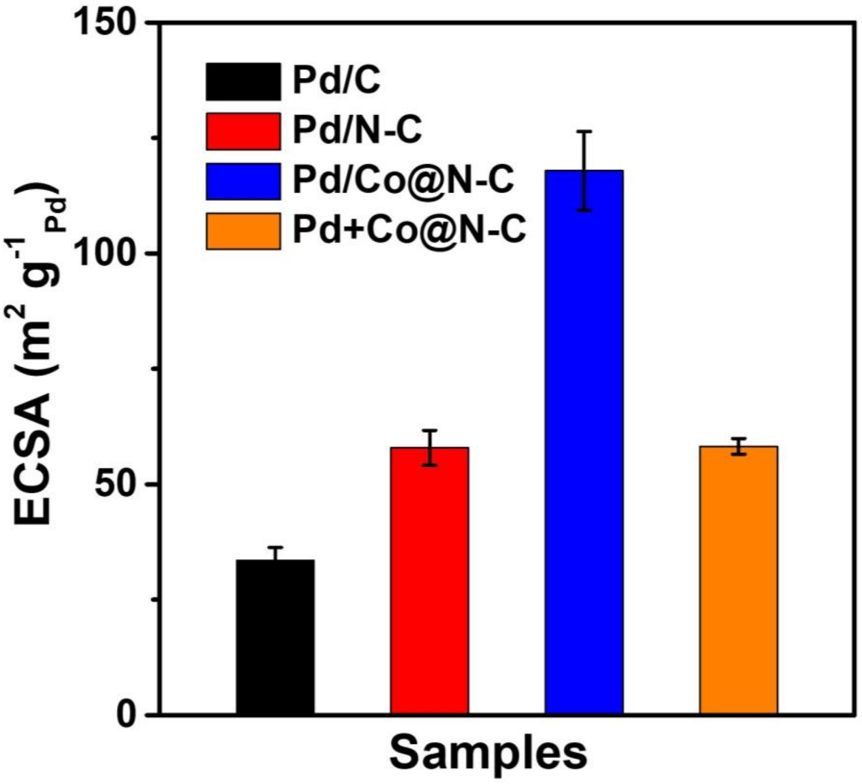

Supplementary Fig. 25. ECSA of different samples calculated from CO stripping. The Pd/Co@N-C shows a much higher ECSA than other control samples, indicating that more active sites on Pd/Co@N-C can be used for electrocatalytic reactions. The error bars represent the s.d. of at least three independent measurements, and the data are presented as mean values ± s.d.

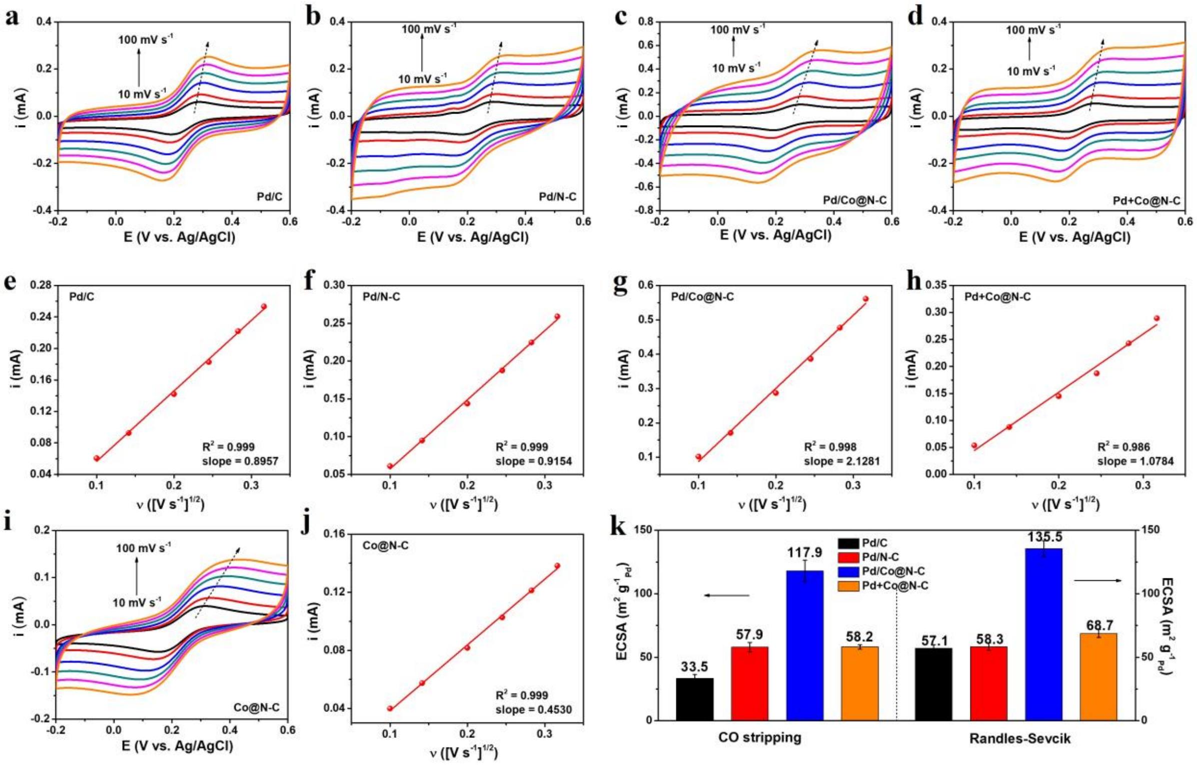

Supplementary Fig. 26. Determination of the ECSA of different samples by Randles-Sevcik equation. (a-d, i) CV curves of different samples in 5 mM $K_3[Fe(CN_6)]$/ $K_4[Fe(CN_6)]$ solution containing 0.1 M KNO_3 at different scan rates (10, 20, 40, 60, 80, and 100 mV s^{-1}), and **(e-h, j)** the corresponding relationships of peak current (I_p) vs. scan rate ($v^{1/2}$). **(k)** The ECSA (m^2 g^{-1}) calculated from CO stripping method (left) and the Randles-Sevcik method (right). The error bars in **(k)** represent the s.d. of at least three independent tests, and the data are presented as mean values ± s.d.

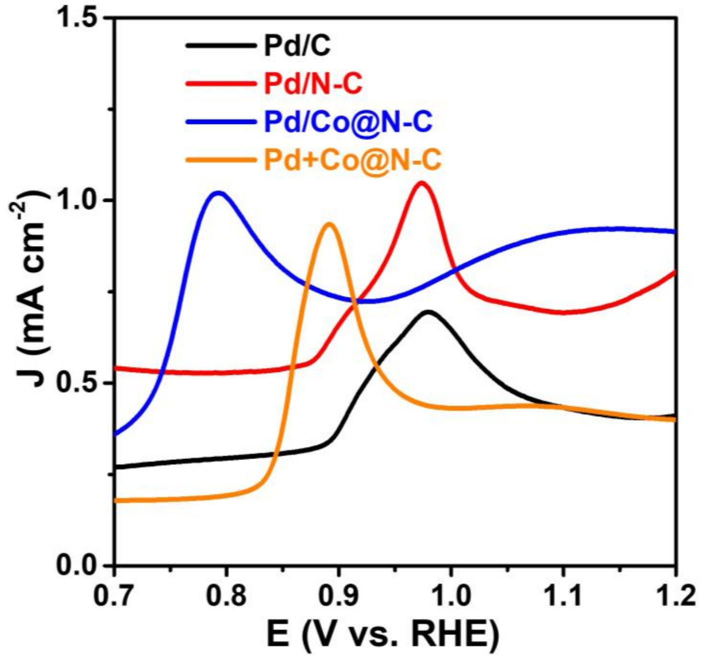

Supplementary Fig. 27. The CO oxidation peak on different samples with a scan rate of 20 mV s^{-1}. The figure is extracted from **Supplementary Fig. 24.**

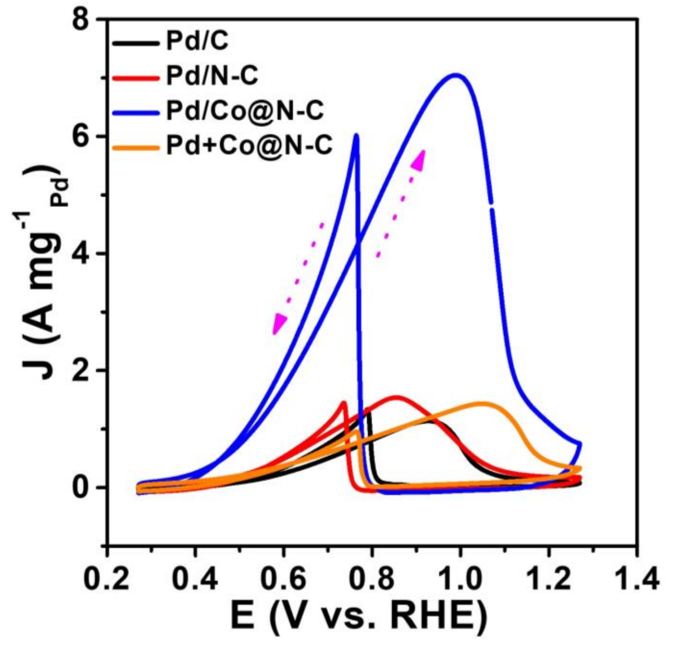

Supplementary Fig. 28. CV curves of different samples in the N_2-saturated 1 M KOH + 1 M EtOH at a scan rate of 50 mV s^{-1}.

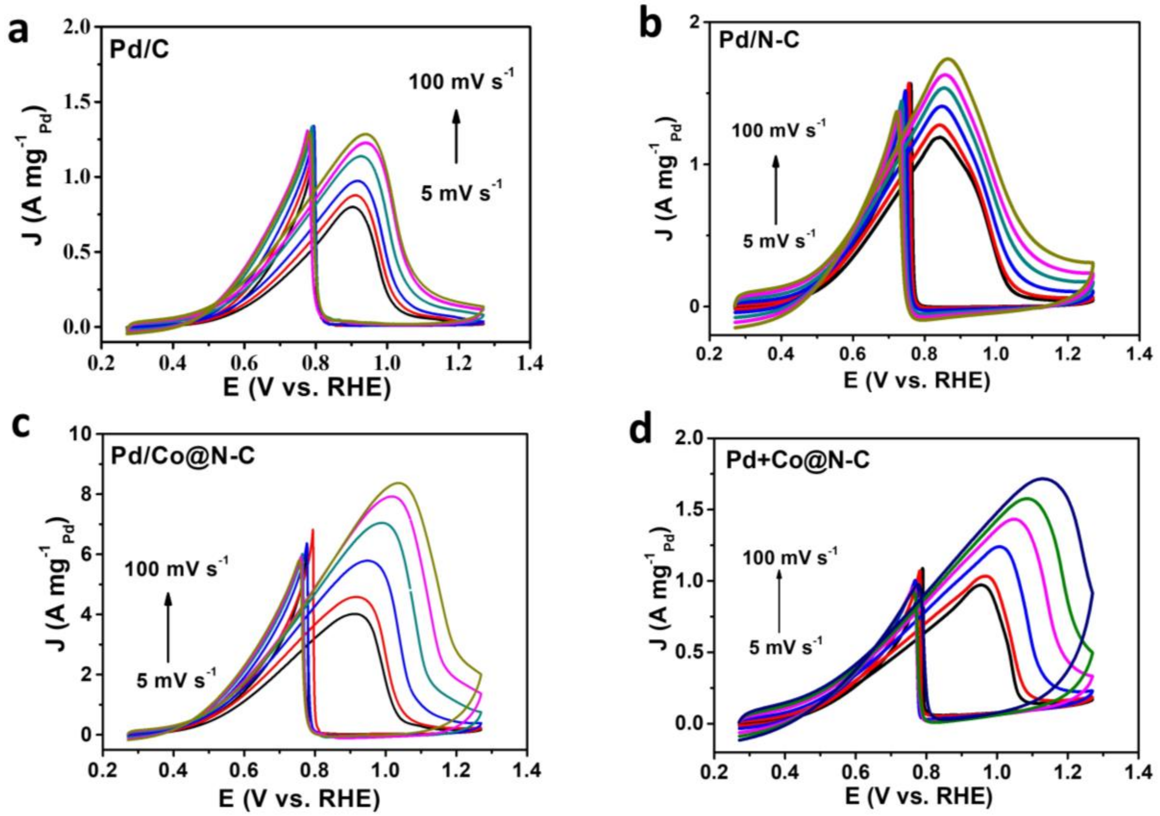

Supplementary Fig. 29. CV curves of EOR on **(a)** Pd/C, **(b)** Pd/N-C, **(c)** Pd/Co@N-C, and **(d)** Pd+Co@N-C catalysts in 1.0 M KOH + 1.0 M EtOH aqueous solution at different scan rates (5, 10, 25, 50, 75, and 100 mV s^{-1}). The current densities (*J*) for EOR significantly increase with the increased scan rate, indicating that the diffusion of reactants and intermediates was the rate-determining step. The slope for Pd/Co@N-C (**Fig. 3c**) is much higher than that of other samples, indicating that the Pd/Co@N-C has much faster kinetics than other samples.

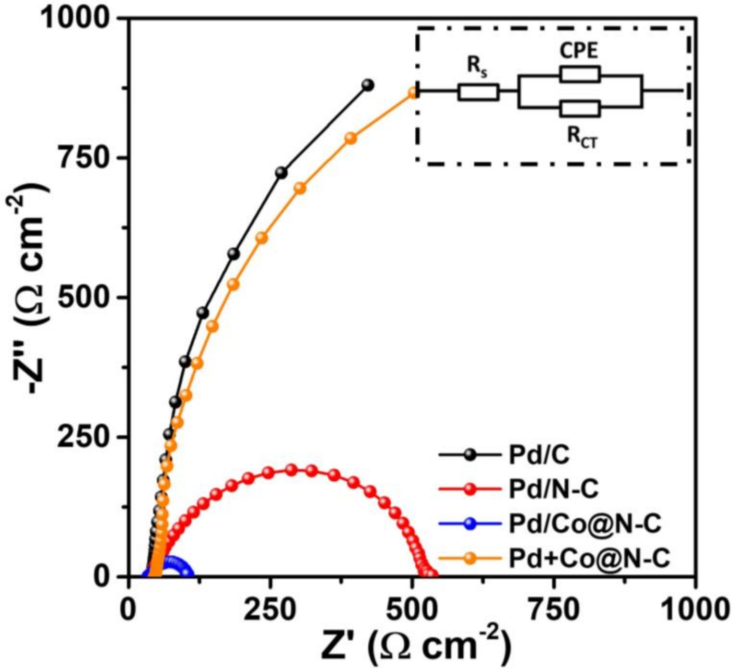

Supplementary Fig. 30. Nyquist plots of different samples. The EIS was tested in 1 KOH + 1 EtOH recorded at 0.6 V_{RHE}. Inset is the equivalent electric circuit and the corresponding charge transfer resistance (R_{CT}), constant phase element (CPE), and system resistance (R_S). The R_{CT} of Pd/Co@N-C was much smaller than those of other samples, suggesting that the Pd/Co@N-C interface can significantly promote electron transfer for EOR.

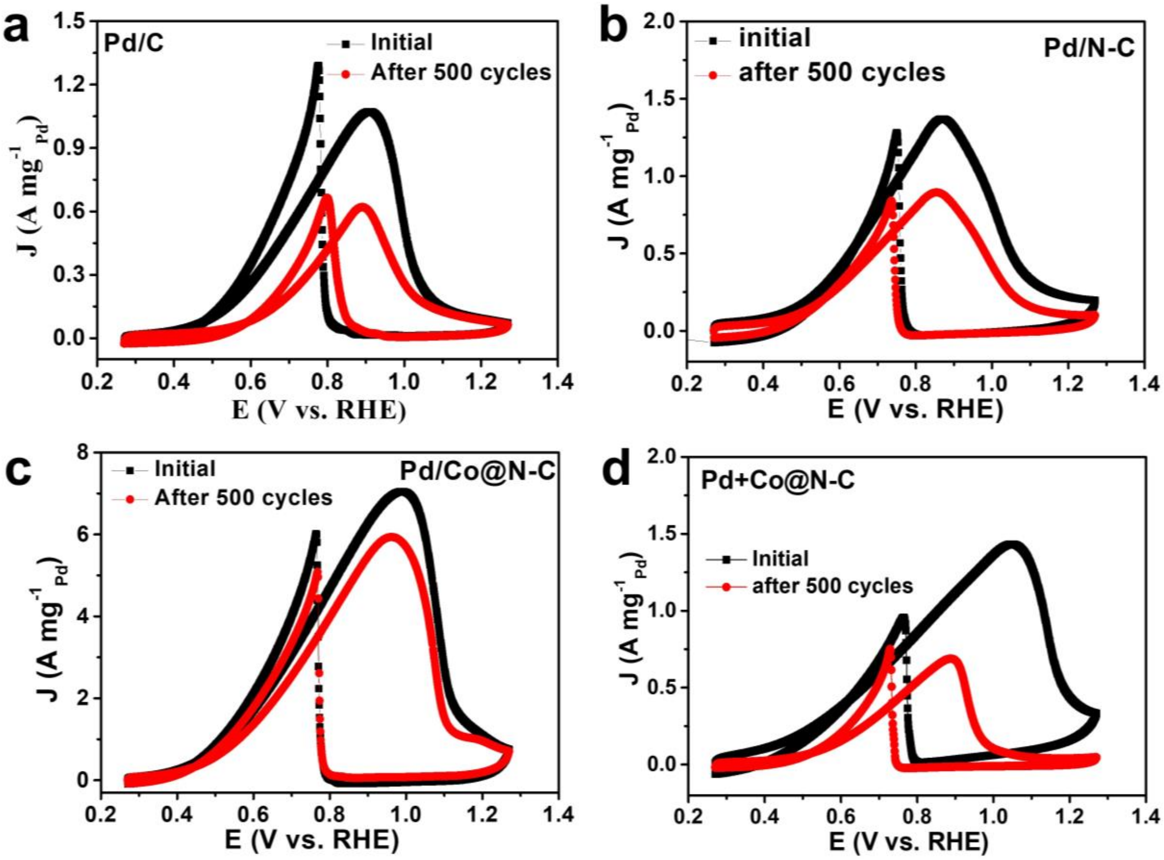

Supplementary Fig. 31. Stability of different samples. CVs curves before and after 500 cycles of stability tests for **(a)** Pd/C **(b)** Pd/N-C, **(c)** Pd/Co@N-C, and **(d)** Pd+Co@N-C catalysts in 1.0 M KOH + 1.0 M EtOH aqueous solution at a scan rate of 50 mV s^{-1}. The Pd/Co@N-C shows a much higher mass activity retention than other samples, indicating the good stability of Pd/Co@N-C.

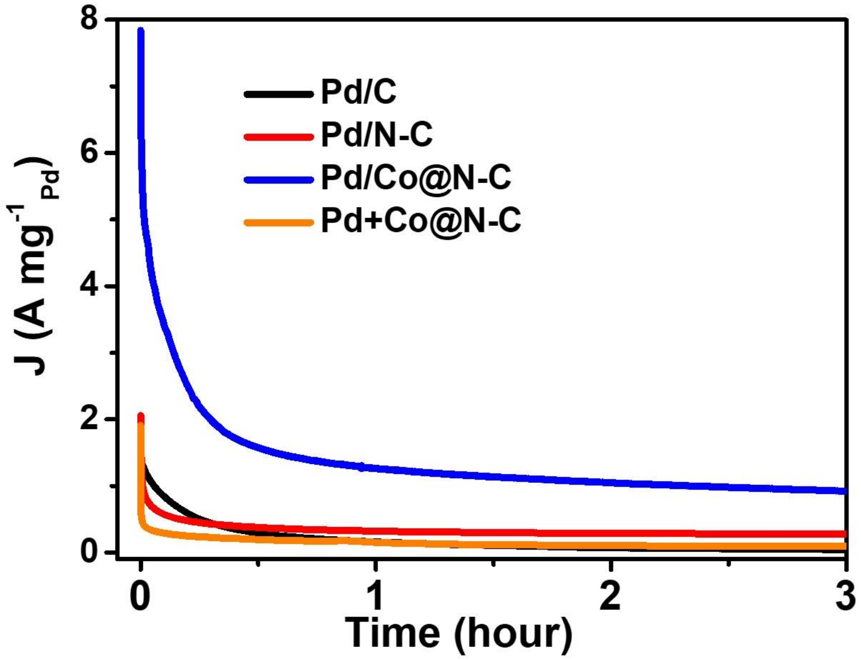

Supplementary Fig. 32. Stability tests of different catalysts using the chronoamperometric curves. The tests were performed in 1.0 M KOH + 1.0 M EtOH aqueous solution at the potential of 0.9 V vs. RHE. The Pd/Co@N-C shows a much higher mass activity retention than other samples, indicating the good stability of Pd/Co@N-C.

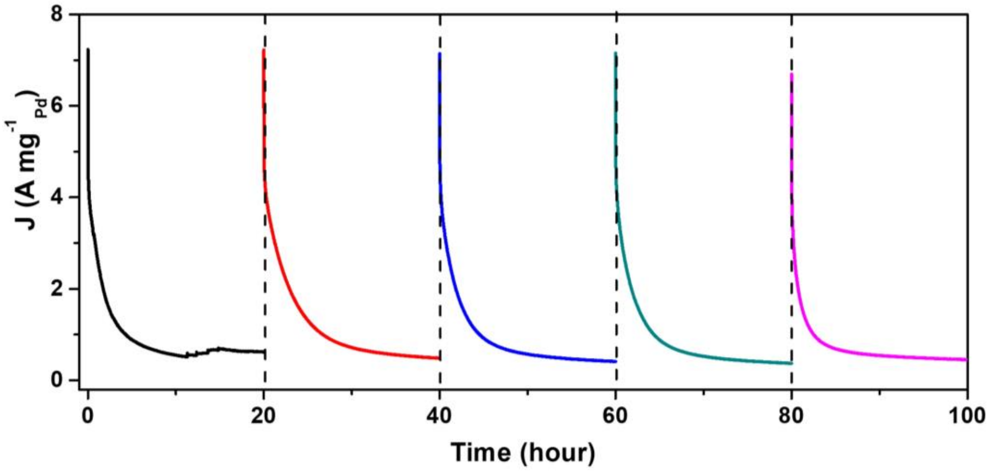

Supplementary Fig. 33. Long-term durability measurements of Pd/Co@N-C by consecutive reactivation in 1.0 M KOH + 1.0 M EtOH at 0.9 V (vs. RHE). No obvious decay can be found during five consecutive reactivation cycles, verifying the outstanding stability and anti-poisoning performance due to the presence of the synergistic interface in Pd/Co@N-C.

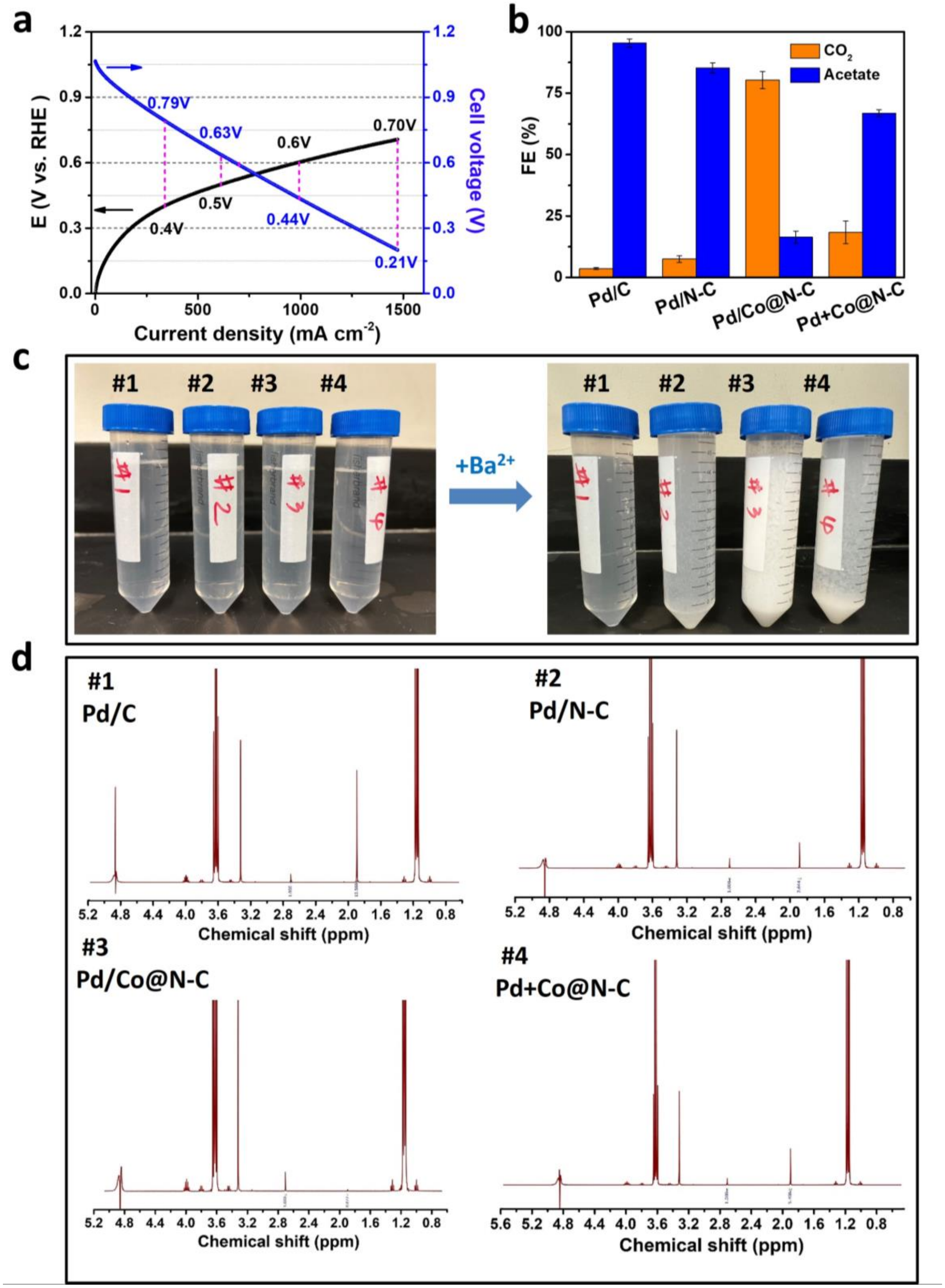

Supplementary Fig. 34. **Determination of potential ranges of EOR and corresponding FE from EtOH to carbonate and acetate. (a)** The anode polarization curves and overall fuel cell

polarization curves of DEFC with an external reference Hg/HgO electrode. Pd/Co@N-C as both anode and cathode catalysts, the anode electrolyte is 1M KOH + 2 M EtOH with a flow rate of 5 mL min^{-1}, and the cathode was fed with oxygen with 200 mL min^{-1} without backpressure. **(b)** Faraday efficiency (FE) of EtOH to CO_2 and acetate on Pd/C, Pd/N-C, Pd/Co@N-C, Pd+Co@N-C at 0.4 V vs. RHE. The error bars in **(b)** represent the s.d. of three independent tests, and the data are presented as mean values ±s.d. **(c)** The typical optical photograph of reacted electrolyte before and after adding excessive $Ba(OH)_2{\cdot}8H_2O$ to titrate the CO_3^{2-} for different catalysts after 3 hours i-t test at 0.4 V vs. RHE. After titration, a lot of white flocculent $BaCO_3$ precipitations were obtained for Pd/Co@N-C sample, indicating that complete 12e pathway for EOR. While tiny turbid liquid was found for Pd/N-C and Pd+Co@N-C, indicating that a small amount of CO_2 was generated on these two electrodes. In contrast, there is no change in the electrolytes with the Pd/C electrode, indicating that almost no CO_2 was generated on Pd/C. **(d)** The ^{1}H-NMR results of different samples operated at 0.4 V vs. RHE for 3 hours. #1, #2, #3, and #4 in **(c-d)** represent Pd/C, Pd/N-C, Pd/Co@N-C, and Pd+Co@N-C catalysts, respectively. The peak at ~1.9 ppm is the characteristic peak of acetate (CH_3COO^-) due to the incomplete oxidation of EtOH. On Pd/C, Pd/N-C, and Pd+Co@N-C, this peak can be seen clearly, while no peaks at ~1.9 ppm can be found on Pd/Co@N-C indicating the complete EOR on Pd/Co@N-C.

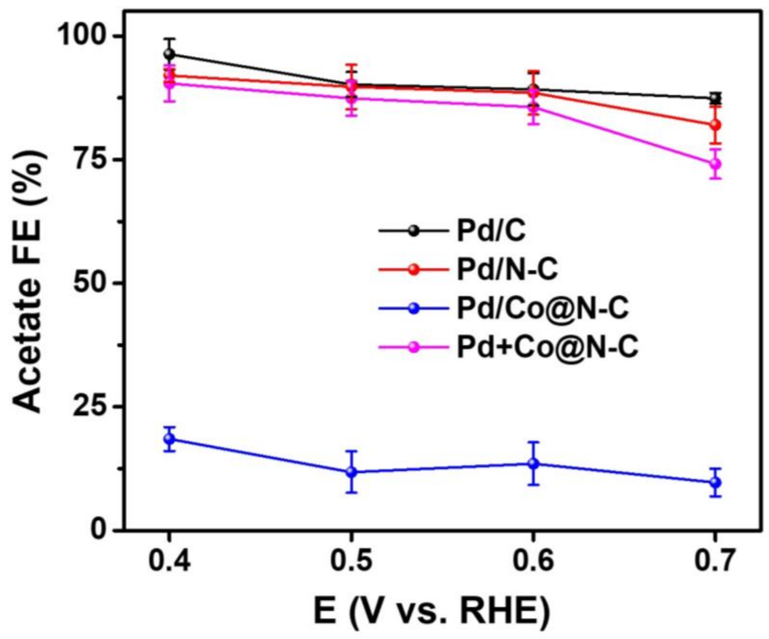

Supplementary Fig. 35. Faradaic efficiency (FE) of acetate at different potentials on different catalysts determined by H^1-NMR. The Pd/Co@N-C shows much lower FE for acetate, further indicating the complete C1-12e pathway for EOR. The error bars represent the s.d. of three independent tests, and the data are presented as mean values ±s.d.

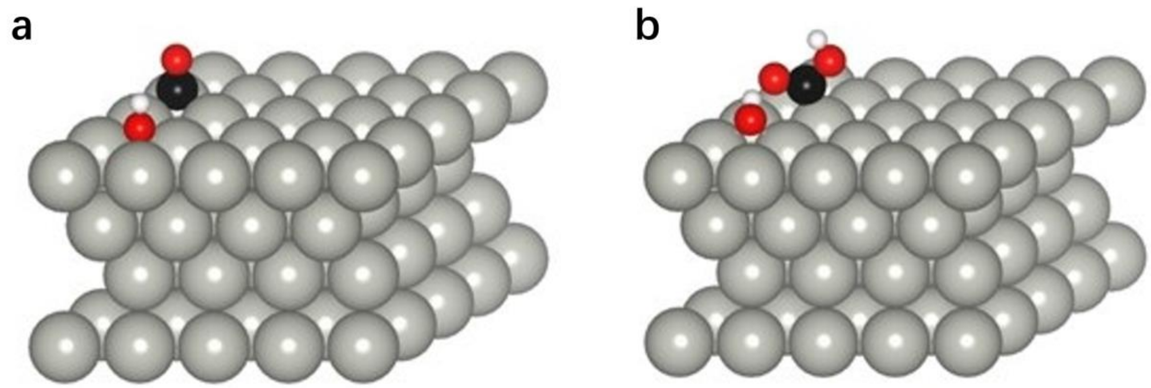

Supplementary Fig. 36. Optimized atomistic structures of (a) CO adsorption, and (b) COOH adsorption on OH-Pd(111) surface. In the figure, the gray, red, black, and white balls represent Pd, O, C, and H atoms, respectively.

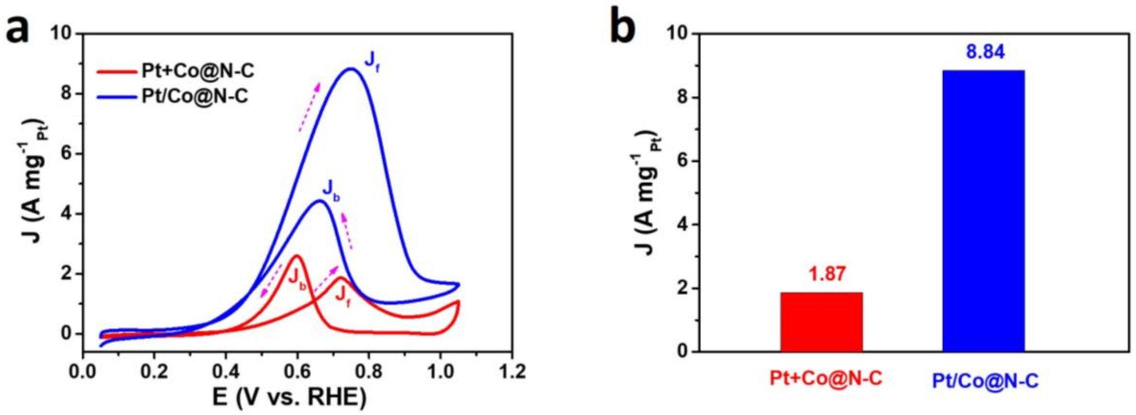

Supplementary Fig. 37. Electrocatalytic EOR performance of Pt/Co@N-C and physically mixed Pt+Co@N-C catalysts. (a) Cyclic voltammogram (CV) curves in the N_2-saturated 1 M KOH + 1 M EtOH at a scan rate of 50 mV s^{-1}. The dashed arrows indicate the scan direction. **(b)** Peak current density (*J*) normalized to Pt mass loading of physically mixed Pt+Co@N-C and Pt/Co@N-C catalysts.

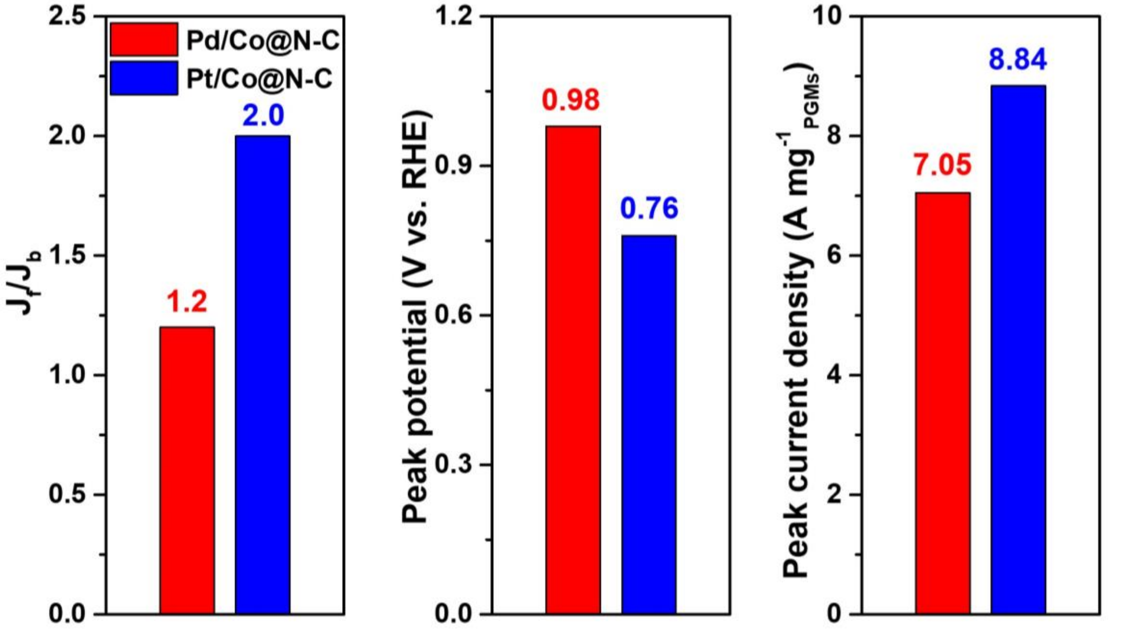

Supplementary Fig. 38. Comparison of EOR performance of Pd/Co@N-C and Pt/Co@N-C. (a) The J_f/J_b values of Pd/Co@N-C and Pt/Co@N-C. The values of J_f/J_b can directly reflect the anti-poisoning property for CO. **(b)** The potentials of Pd/Co@N-C and Pt/Co@N-C to reach the EOR peak current density. **(c)** The EOR peak current density of Pd/Co@N-C and Pt/Co@N-C.

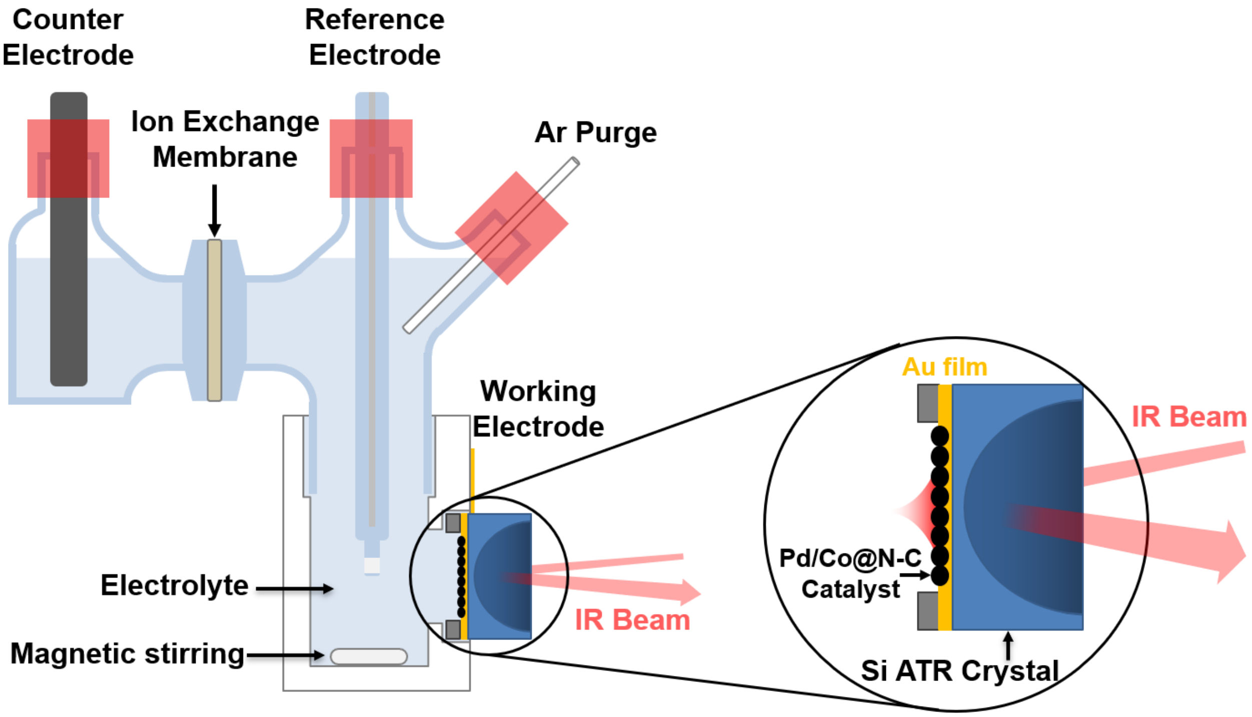

Supplementary Fig. 39. Schematic of the spectroelectrochemical cell. The stirred spectroelectrochemical cell with Pd/Co@N-C catalyst as a working electrode, graphite rod counter electrode, and an Ag/AgCl reference electrode. The Pd/Co@N-C catalyst was deposited on a SEIRAS active Au film on the Si ATR crystal with a loading of 0.01 and 0.5 mg_{Pd} cm^{-2}, respectively.

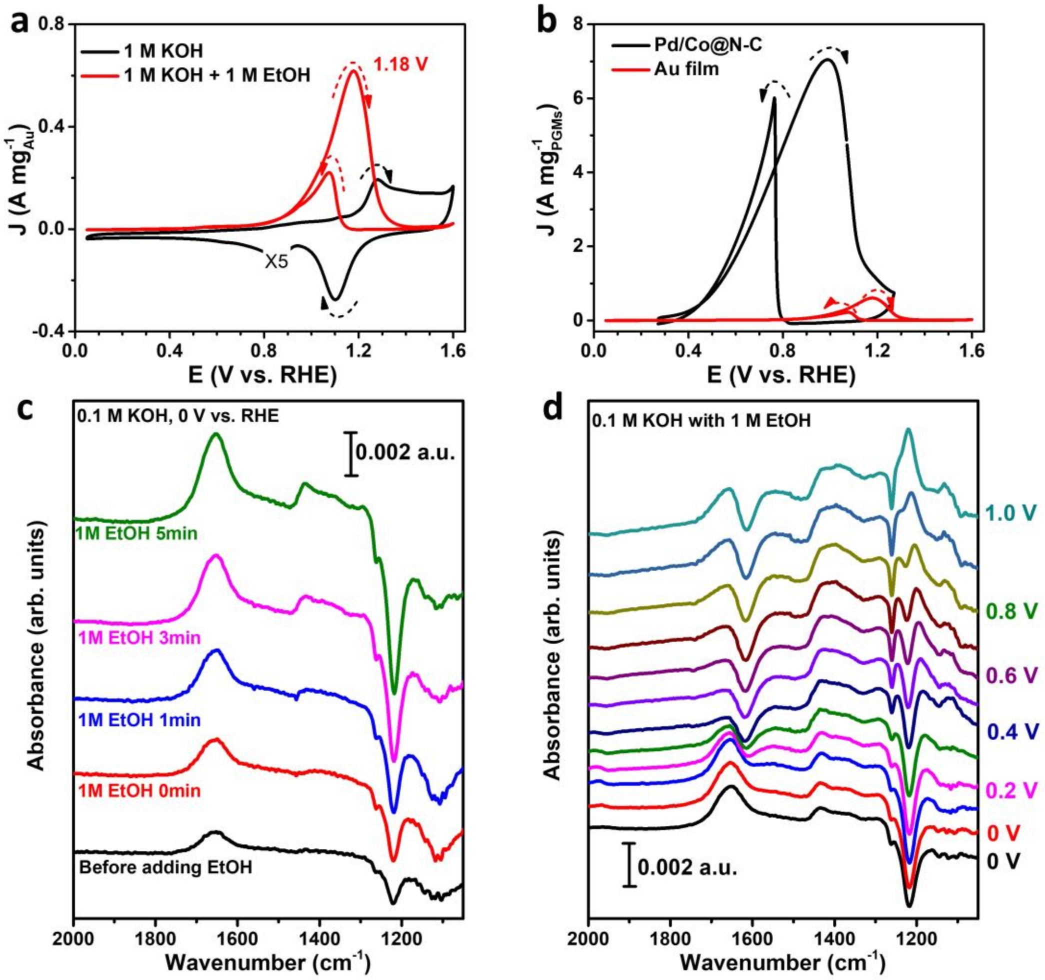

Supplementary Fig. 40. EOR performance of Au film electrode and the *in-situ* ATR-SEIRA spectra. (a) The CV curves of Au film electrode in N_2-saturated 1M KOH with and without 1 M EtOH solution, the scan rate is 50 mV s^{-1}. **(b)** Comparison of the CV curves of Pd/Co@N-C and Au film for EOR in N_2-saturated 1M KOH with 1 M EtOH solution with a scan rate of 50 mV s^{-1}. **(c)** *In-situ* ATR-SEIRA spectra measured on Au/Si prism electrode before and after adding 1 M EtOH in 0.1 M KOH aqueous solution at 0 V vs RHE with different time. **(d)** *In-situ* ATR-SEIRA spectra measured at different potentials for Au/Si prism electrode in 0.1 M KOH + 1.0 M EtOH aqueous solution. The reference spectra (black lines) in (d) were obtained at 0 V in 0.1 M KOH aqueous solution.

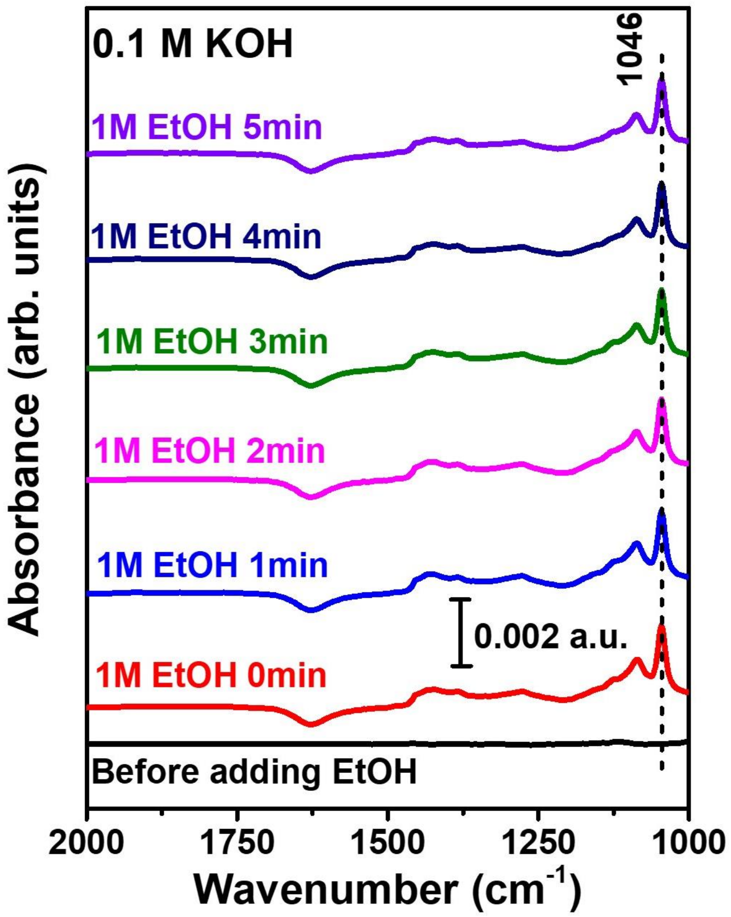

Supplementary Fig. 41. ***In-situ* ATR-SEIRA spectra of Si prism** in 0.1 M KOH with and without 1 M EtOH solutions. In 0.1 M KOH solution, no peak at ~1046 cm^{-1} can be found. In contrast, after 1 M EtOH was introduced, a sharp and strong peak at ~1046 cm^{-1} can be found clearly, which strongly confirmed that the 1046 cm^{-1} signal peak was come from EtOH, rather than from Si.

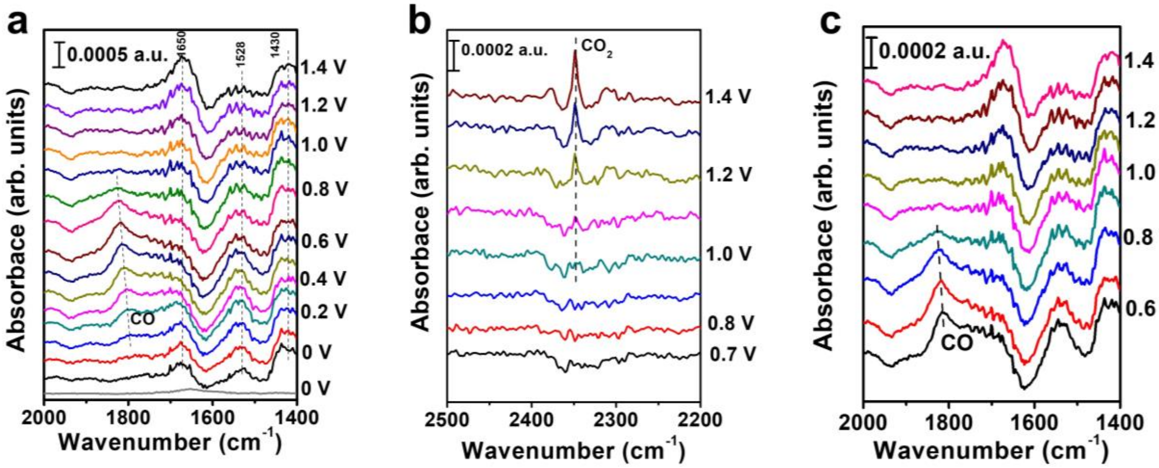

Supplementary Fig. 42. ***In-situ*** **ATR-SEIRA spectra** measured at different potentials for Pd/Co@N-C in 0.1 M KOH + 1.0 M EtOH aqueous solution in the **(a)** window from 1400 to 2000 cm^{-1}, **(b)** CO_2 spectral window from 2200 to 2500 cm^{-1}, and **(c)** CO spectral window from 1400 to 2000 cm^{-1}. The reference spectra in **(a)** (gray lines) were obtained at 0 V in 0.1 M KOH aqueous solution. The peak at 1430 cm^{-1} is attributed to the Au/Si background. To obtain the best spectroscopic results, the loading of 0.5 mg$_{Pd}$ cm^{-2} was employed. The CO signal can be found at a much lower potential of 0.2 V vs RHE (**Supplementary Fig. 42a**) in the range of 1800-1828 cm^{-1}, and this characteristic CO peak was found at a wide potential of 0.2-0.9 V due to the continuous C-C bonds cleavage. The CO signal disappears above 0.9 V (**Supplementary Figs. 42a, c**) while the CO_2 characteristic peak can be seen and became stronger as the potential increased from 1.0-1.4 V (**Supplementary Fig. 42b**), indicating that the C-C bond was easily cleaved at the low potential on Pd/Co@N-C and finally be oxidized to CO_2 through a direct C1-12e pathway at a high potential.

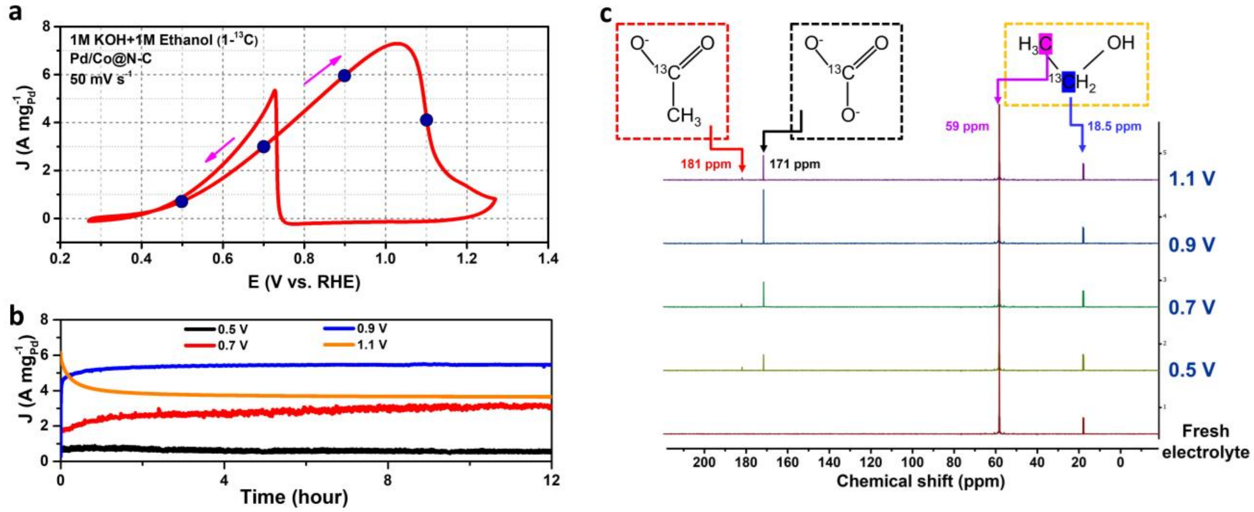

Supplementary Fig. 43. The isotopically labeled measurement for EOR. (a) The CV curves of Pd/Co@N-C electrode in N_2-saturated 1M KOH with 1 M 1-^{13}C labeled EtOH ($CH_3{}^{13}CH_2OH$) solution, the scan rate is 50 mV s^{-1}. The four bold dots were selected to do the chronoamperometric test. **(b)** The EOR chronoamperometric test at the different potentials for 12 hours in N_2-saturated 1M KOH with 1 M $CH_3{}^{13}CH_2OH$ solution. **(c)** ^{13}C-NMR spectroscopy from the electrolyte after 12 hours *i*-*t* test at different potentials on Pd/Co@N-C in 1.0 M KOH + 1.0 M $CH_3{}^{13}CH_2OH$ aqueous solution. The experiments were performed in a sealed and air-free H-type cell with continuous N_2 gas flowing into 12 mL electrolyte (1 M KOH + 1.0 M $CH_3{}^{13}CH_2OH$). After the potentiostatic *i*-*t* test for 12 h (0.5 V, 0.7 V, 0.9 V, and 1.1 V vs. RHE), 0.5 mL of electrolyte was used for the ^{13}C-NMR test immediately. The fresh electrolyte was also tested and used as a reference spectrum.

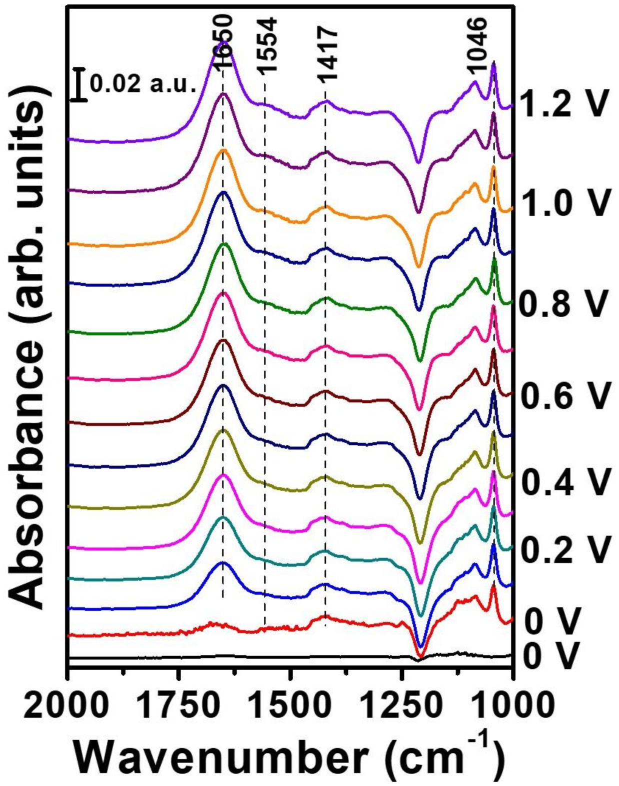

Supplementary Fig. 44. ***In-situ* ATR-SEIRA spectra measured at different potentials for Pt/C** in 0.1 M KOH + 1.0 M EtOH aqueous solution. The reference spectra (black lines) were obtained at 0 V in 0.1 M KOH aqueous solution. The characteristic peak for the CO stretching vibration of C_2H_5OH at 1046 cm^{-1} was observed. The peak at 1417 cm^{-1} is attributed to the Au/Si background. The peak at 1650 cm^{-1} is attributed to the interfacial H_2O. No CO characteristic peaks at ca. 1800-1828 cm^{-1} were found. As CO_{ads} is the direct signal for the C-C bond cleavage during EOR complete oxidation, the results prove that the Pt/C samples show a powerless property for complete EOR, which results in an inferior activity than Pd/Co@N-C.

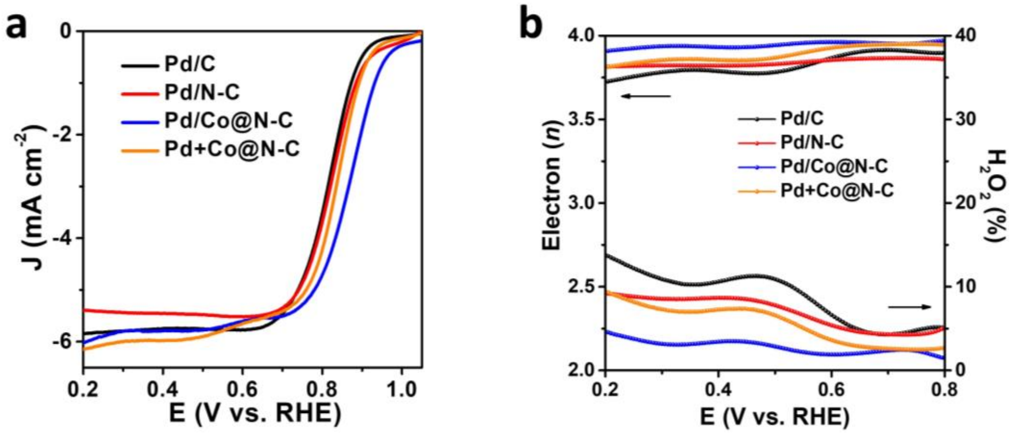

Supplementary Fig. 45. ORR performance of different samples in the O_2 saturated 0.1 M KOH solution. (a) ORR polarization curves at a scan rate of 5 mV s^{-1} at a rotating speed of 1,600 rpm and the corresponding **(b)** electron transfer number (*n*, left) and H_2O_2 production (right) by RRDE test.

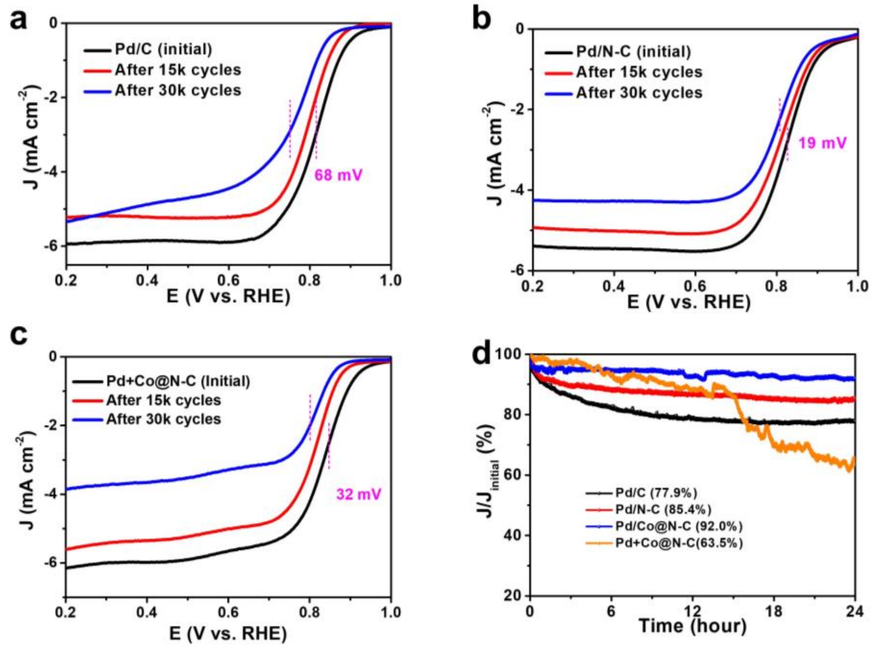

Supplementary Fig. 46. ORR stability of different samples. LSV curves before and after 15k and 30k cycling tests for **(a)** Pd/C, **(b)** Pd/N-C, and **(c)** Pd+Co@N-C in the O_2-saturated 0.1 M KOH aqueous solution at a scan rate of 5 mV s^{-1} and 1600 rpm. **(d)** The 24h *i-t* test at 0.6 V. About 92% of the initial current density was retained for Pd/Co@N-C, which is much higher than those of Pd/C (77.9%), Pd/N-C (85.4%), and Pd+Co@N-C (63.5%).

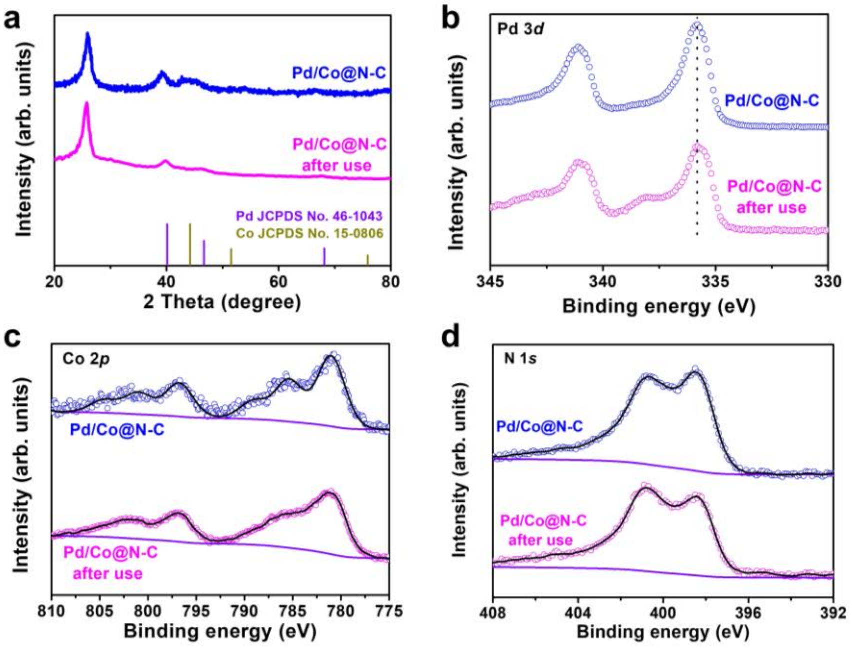

Supplementary Fig. 47. Structure and composition characterizations of Pd/Co@N-C after 30k cycles AST test. (a) XRD and XPS of **(b)** Pd 3*d*, **(c)** Co 2*p*, and **(d)** N 1*s*. The crystallinity and chemical states of Pd/Co@N-C remain unchanged compared with the pristine state, confirming the excellent long-term electrochemical stability of Pd/Co@N-C.

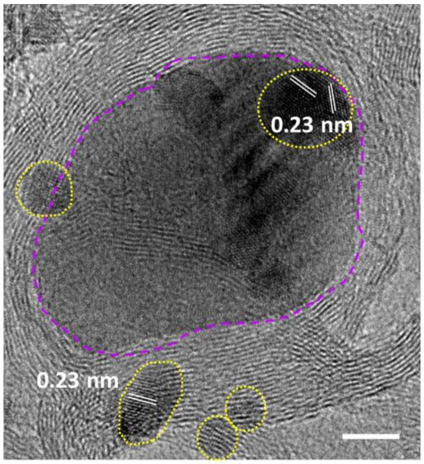

Supplementary Fig. 48. HR-TEM of Pd/Co@N-C after 30k cycles AST test. The yellow dashed areas are Pd NPs, and the purple dashed areas are Co NPs. The lattice spacings of 0.23 nm are corresponding to the Pd (111) crystal plane. The Pd/Co@N-C well preserves its original structural and chemical integrity after the AST tests. No obvious change can be found compared with the fresh samples. Scale bar: 5 nm.

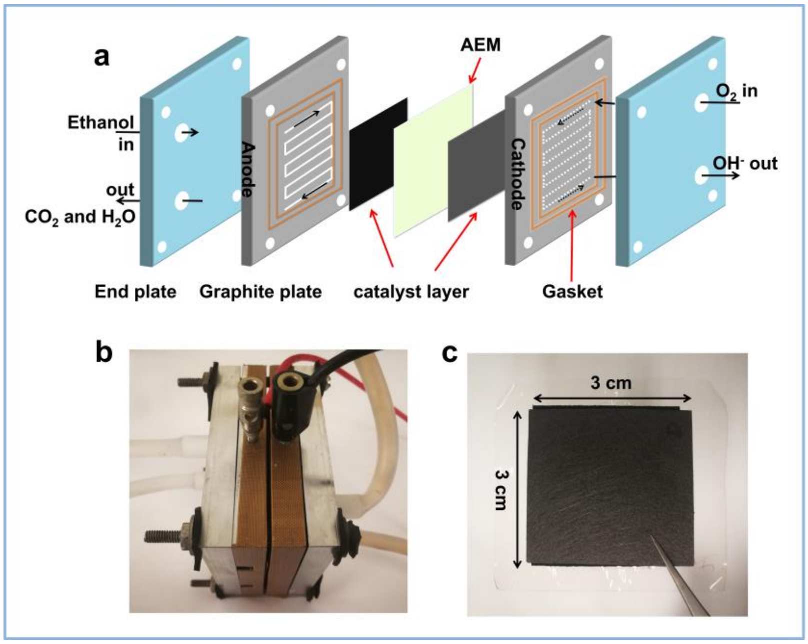

Supplementary Fig. 49. The reactor used for the DEFCs performance test. (a) Schematic illustration and **(b)** photograph of DEFCs. **(c)** Photograph of MEA composed of an anion-exchange membrane (AEM) and two catalyst layers (Pd/Co@N-C with a loading of 0.1 mg_{Pd} cm^{-2} on MEA).

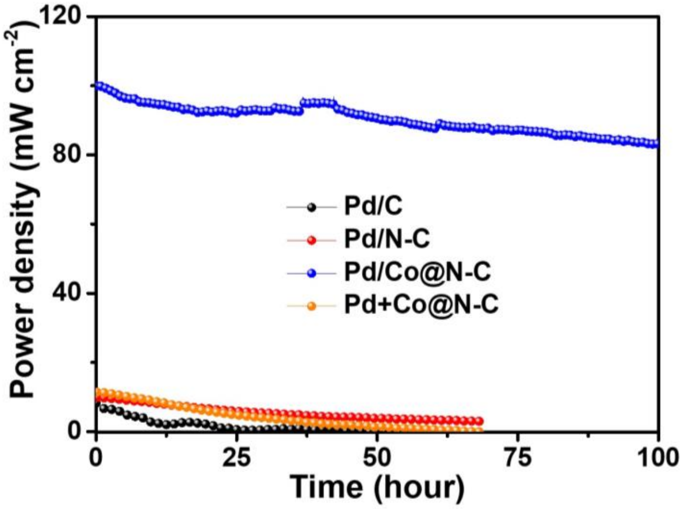

Supplementary Fig. 50. Discharge curves for DEFCs at 0.4 V with different catalysts. Pd loading is 0.1 mg cm^{-2} on MEA. The Pd/Co@N-C shows much higher activity and stability than other control samples.

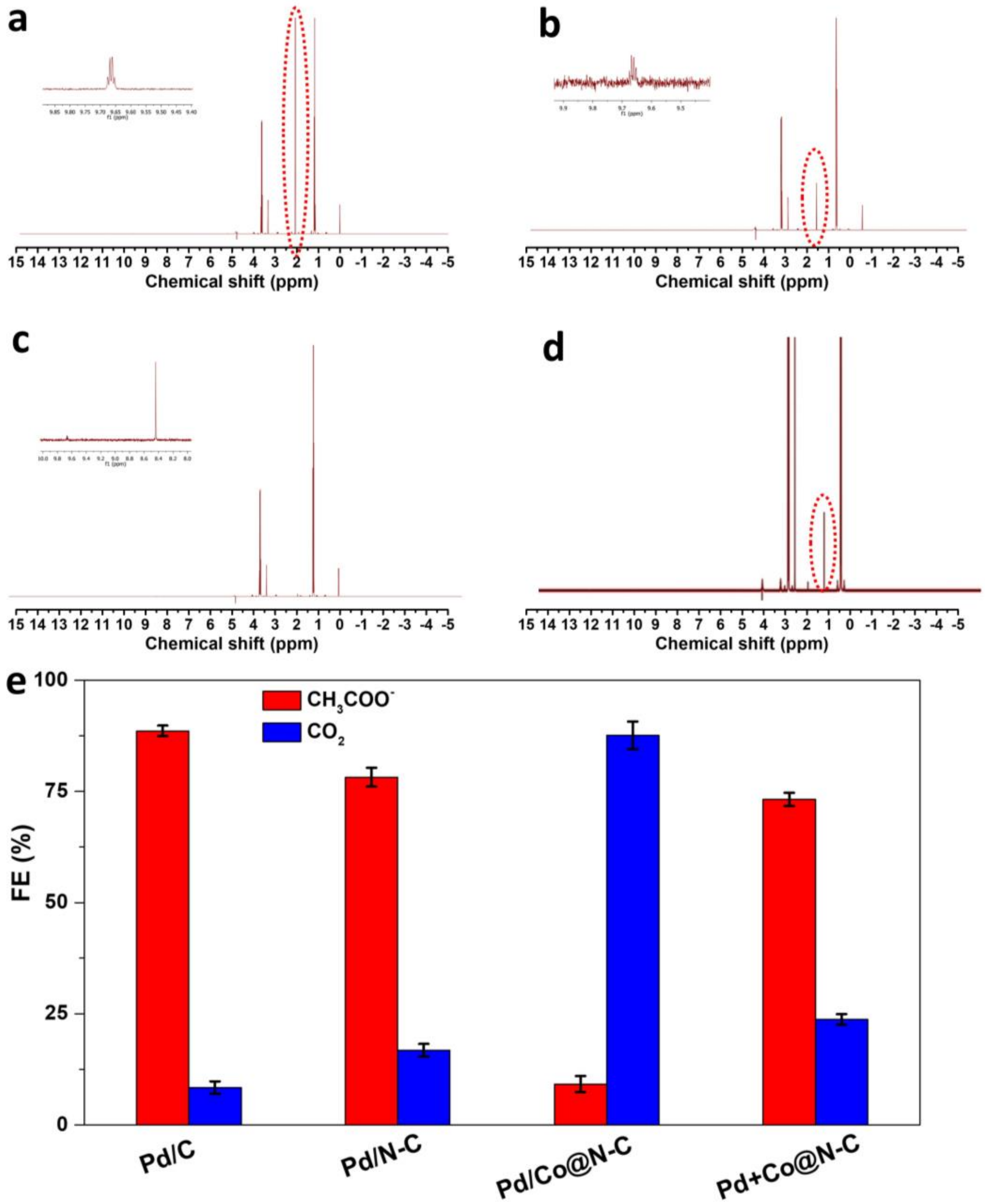

Supplementary Fig. 51. FE of acetate and CO_2 on DEFCs. ^{1}H-NMR spectra from the anode exhaust were collected at 0.4 V for 24 hours of operation of DEFCs with **(a)** Pd/C, **(b)** Pd/N-C, **(c)** Pd/Co@N-C, and **(d)** Pd+Co@N-C as the anode catalysts in 2 M EtOH aqueous solution. The peak at ~2.1 ppm is the characteristic peak of acetic acid (CH_3COOH) formed by the incomplete EOR. In contrast, no peaks at ~2.1 ppm can be found on Pd/Co@N-C, indicating the complete EOR on the Pd/Co@N-C. **(e)** The FE of acetate and CO_2 on DEFCs. The error bars in (e) represent the s.d. of three independent tests, and the data are presented as mean values ±s.d.

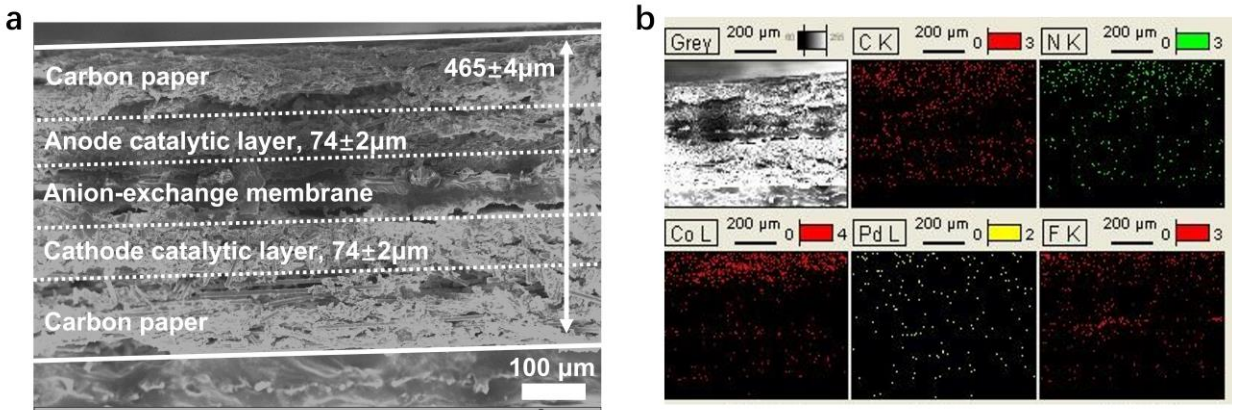

Supplementary Fig. 52. Cross-sectional SEM image of the MEA. (a) SEM and corresponding **(b)** EDX-mapping images of freshly prepared MEA. The F element was from the AEM membrane. No delamination phenomenon was found, indicating good contact between the catalyst and membrane. With a such thin catalytic layer, water can migrate from anode to cathode through AEM. While the cathode is feeding with 100% relative humidity of O_2, which can ensure unrestrained movement of OH^- in the catalytic layer from cathode to anode.

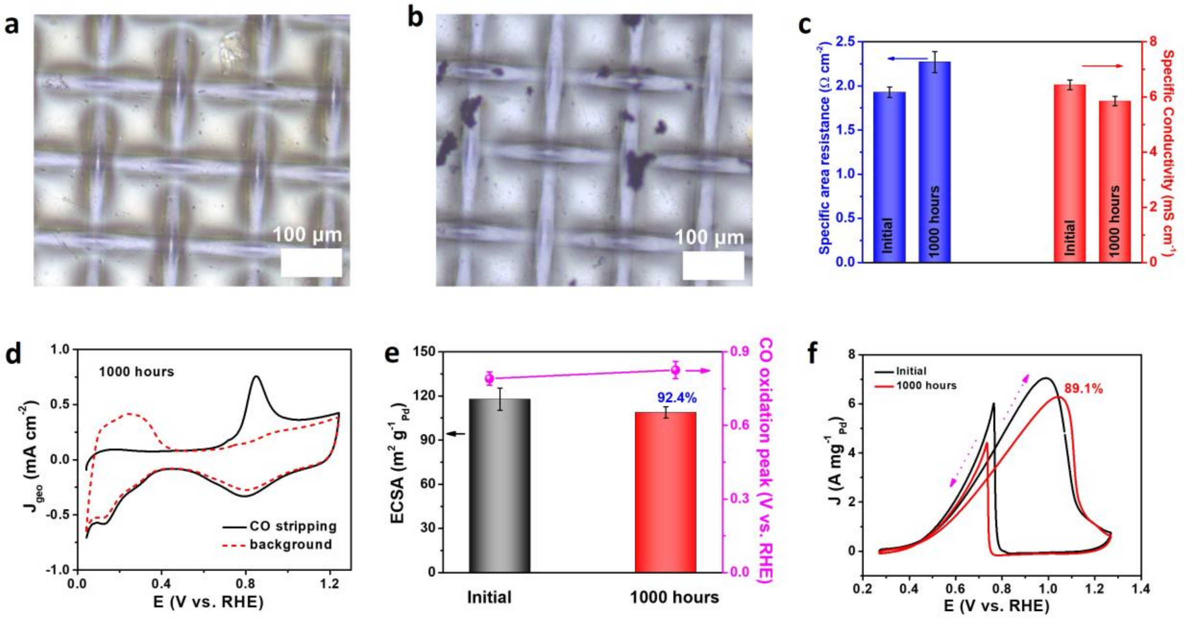

Supplementary Fig. 53. Effect of carbonates on the membrane and catalyst. Optical images of the anion-exchange membrane **(a)** before and **(b)** after 1000 hours test. The black areas in (b) were Pd/Co@N-C catalyst, which was strongly contacted with the membrane due to hot press during the preparation of MEA. **(c)** Specific area resistance (left axis) and specific conductivity (right axis) of AEM before and 1000 hours test. **(d)** CO stripping CV curves of Pd/Co@N-C after 1000 hours stability test. The CV curves were conducted in 0.1 M KOH at a scan rate of 20 mV s^{-1}. **(e)** ECSA (left axis) and CO oxidation peak potential (right axis) of Pd/Co@N-C before and after 1000 hours stability test, the ECSA was calculated from the charge integration of CO stripping that derived from (d) and **Supplementary Fig. 24c**. **(f)** CV curves for EOR before and after 1000 hours stability test in N_2-saturated 1M KOH + 1M EtOH at a scan rate of 50 mV s^{-1}. The mass activity retention ratio of Pd/Co@N-C after 1000 hours of stability test was 89.1%, indicating the negligible effect of carbonate on the catalyst. The error bars in (c) and (e) represent the s.d. of three independent measurements, and the data were presented as mean values ±s.d.

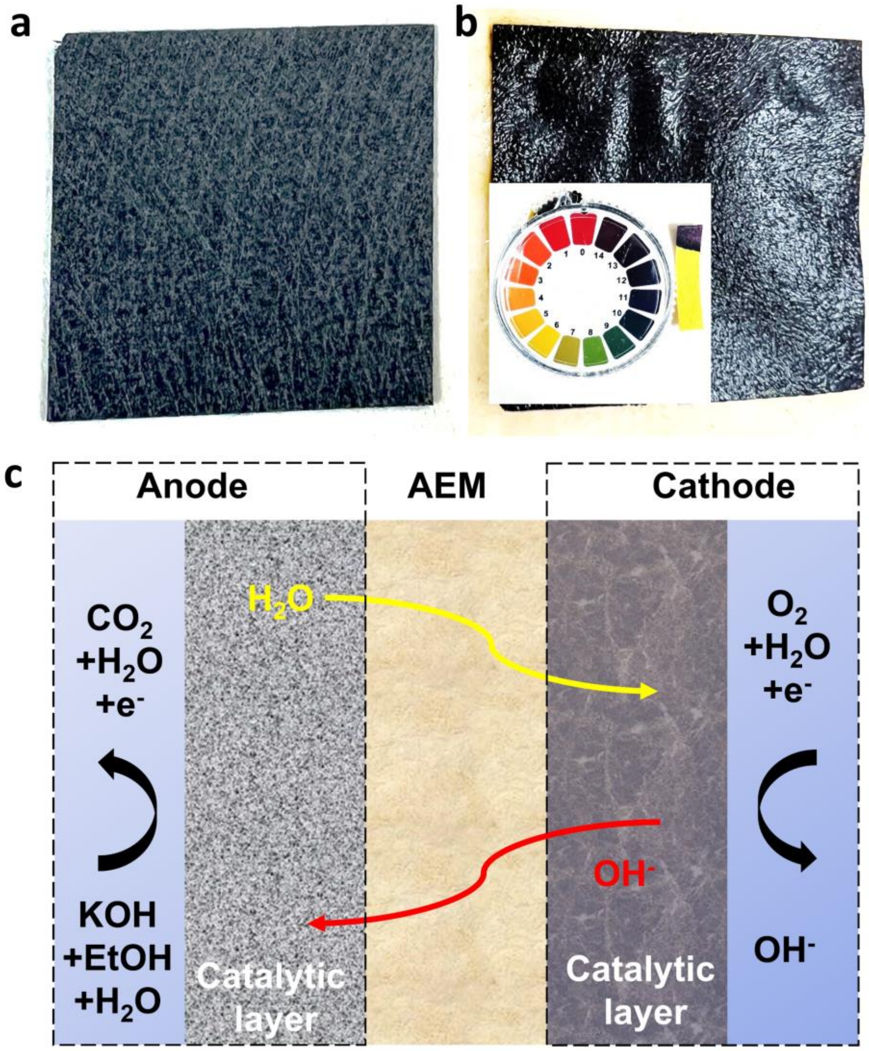

Supplementary Fig. 54. The movement of H_2O and OH^- in the catalytic layer. (a) A piece of fresh carbon paper and **(b)** cathode catalytic layer (inset shows the corresponding pH) after 1000 hours stability test, a fully wetted cathode catalyst layer with a pH > 11 was found. **(c)** A schematic of DEFC that shows the movement of water (H_2O) and hydroxide radical (OH^-). The anode is fed with 1 M KOH + 2 M EtOH solution; thus, the water can move from the anode to the cathode through AEM (yellow arrows). The cathode was fed with 100% relative humidity of O_2, which can ensure unrestrained movement of OH^- in the catalytic layer from cathode to anode (red arrows).

Supplementary Tables

Supplementary Table 1. The element contents of Pd, Co, N, and C in Pd/Co@N-C.

Element	at% (XPS)	wt% (XPS)	at% (EDX)	wt% (EDX)	Average (at%)	Average (wt%)
**Pd**	0.62	4.80	0.66	5.14	0.64	4.97
**Co**	2.45	10.48	2.30	9.85	2.375	10.165
**N**	2.83	2.87	2.26	2.30	2.545	2.585
**C**	94.10	81.85	94.78	82.71	94.44	82.28

Supplementary Table 2. The metal content (wt%) determined by the ICP analysis for different catalysts ('-' represents "cannot be detected"). The error bars represent the s.d. of three independent tests and data are presented as mean values+/- s.d.

Sample	Pd	Co	Zn
**Pd/Co@N-C**	4.98±0.01	10.38±0.01	0.02±0.01
**Commercial Pd/C (10 wt%)**	9.96±0.02	-	-
**Pd/N-C**	5.01±0.01	-	0.03±0.01
**Pd+Co@N-C**	4.97±0.02	10.55±0.01	0.02±0.01
**Pd/Co-N-C**	5.25±0.01	9.49±0.02	-
**Pd/Co@C**	5.03±0.01	11.37±0.01	-

Supplementary Table 3. The position of Pd 3d, work function (ϕ), and content of Pd^0 of the samples ('-' represents unknown data). Based on the NIST XPS Database (https://srdata.nist.gov/xps/EngElmSrchQuery.aspx?EType=PE&CSOpt=Retri_ex_dat&Elm=Pd), the location of metallic Pd ranges from the 339.3~341.1 eV (Pd $3d_{3/2}$) and 335.0~336.0 eV (Pd $3d_{5/2}$). The predominant presence of the Pd in all samples is the metallic phase (Pd^0), except the commercial Pd/C is mainly in the oxidized state (Pd^{2+}).

Samples	Pd $3d_{5/2}$ (eV)	Pd $3d_{3/2}$ (eV)	ϕ (eV)	Content of Pd^0
Pd/Co@N-C	335.8	341.1	5.17	87.2%
Pd/N-C	335.3	340.6	5.29	73.3%
Commercial Pd/C	335.3	340.6	5.37	35.6%
Pd NPs	-	-	5.08	-
Pd+Co@N-C	335.3	340.6	5.40	70.7%
Pd/Co@C	335.3	340.6	5.40	75.4%
O-Pd/Co@N-C	335.3	340.6	-	42.2%

Supplementary Table 4. The predicted number of electrons transferred from the Pd cluster to the graphene layer and calculated adsorption energies for Pd NPs adsorbed on p-pydN, pydN, and grapN sites in the graphene layer. Here, adsorption energy was calculated as the energy difference between the adsorbate system and the corresponding isolated system. A more negative value of the adsorption energy represents a stronger interaction between the Pd cluster and the graphene layer.

	grapN site	pydN site	p-pydN site
Adsorption energy (eV)	-1.49	-2.13	-2.70
Number of electrons transferred	1.03	1.06	1.61

Supplementary Table 5. The ECSA, mass activity, and specific activity of benchmarking Pd- and Pt-based catalysts for EOR in alkaline solutions ('-' represents unknown data; the EOR activity was compared at peak current density).

**Catalyst**	**ECSA (m^2 g^{-1})**	**Mass activity (A mg^{-1})**	**Specific activity (mA cm^{-2})**	**Condition (all at room temperature)**	**Reference**
Pd/Co@N-C	115.48	7.06	6.11	1.0 M KOH + 1.0 M EtOH 50 mV s^{-1}	This work
Pd/C	49.05	1.14	3.31		
Pd/N-C	57.9	1.64	2.83		
Pd+Co@N-C	58.20	1.46	2.51		
Pd/$Ni(OH)_2$/rGO	40.3	1.5	*3.72*	1.0 M KOH + 1.0 M EtOH; 50 mV s^{-1}	*Adv. Mater.* **2017,**29, 1703057
Pd@CoP NSs/CFC	40.3	1.41	*3.49*	1.0 M KOH + 1.0 M EtOH; 50 mV s^{-1}	*ACS Catal.***2016**, 6, 7962
Pd NFMs	57.29	0.55	*0.96*	0.5M NaOH + 1.0 M EtOH; 50 mV s^{-1}	*Adv. Mater.* **2012**, 24, 1594
PdCo NTAs/CFC	50.13	1.56	*3.11*	1.0 M KOH + 1.0 M EtOH; 50 mV s^{-1}	*Angew. Chem. Int. Ed.* **2015**, 54, 3669
Au/Pd bimetallic NCs	20	1.06	*5.5*	0.1 M KOH + 0.5 M EtOH; 50 mV s^{-1}	*Angew. Chem. Int. Ed.* **2013**, 52, 645
Pt/Pd bimetallic nanotubes	48.7	1.00	*2.05*	0.5 M NaOH + 0.5M EtOH; 50 mV s^{-1}	*Energy Environ. Sci.* **2010**, 3, 1307
Pd/PANI/Pd SNTAs	35	0.35	*1.0*	1.0 M NaOH + 1.0 M EtOH; 50 mV s^{-1}	*J. Am. Chem. Soc.* **2013**, 135, 10703
Pd–Ni–P nanocatalysts	63.22	4.95	*7.84*	1.0 M NaOH + 1.0 M EtOH; 100 mV s^{-1}	*Nat. Commun.* **2017**, 8, 14136
Pd NMA	-	3.08	-	1.0 M KOH + 1.0 M EtOH; 50 mV s^{-1}	*Adv. Mater.* **2007**, 19, 4256
Pd-Ni_2P/C	87.09	3.49	*4.01*	0.5 M KOH + 1.0 M EtOH; 50 mV s^{-1}	*ChemSusChem* **2014**, 7, 3374
Pd_5Au_1	86.40	1.74	*2.01*	1.0 M KOH + 1.0 M EtOH; 50 mV s^{-1}	*Catal. Sci. Technol.* **2016**, 6, 5397
PtPdAu/C	69.3	1.56	*2.25*	0.5 M NaOH + 1.0 M EtOH; 50 mV s^{-1}	*J.Phy. Chem.C* **2011**, 115, 15324
$Pd_{73}Cu_{27}$	-	~3.0	-	1.0 M KOH + 1.0 M EtOH; 50 mV s^{-1}	*J. Mater. Chem. A* **2014**, 2, 20933
Pd_2Ni_1/C	68.0	1.20	*1.76*	1.0 M NaOH + 1.0 M EtOH; 50 mV s^{-1}	*Int. J. Hydrogen Energy* **2011**, 36, 12686
PdCuCo NCs/C	-	7.72	-	1.0 M NaOH + 1.0 M EtOH; 50 mV s^{-1}	*Angew. Chem. Int. Ed.* **2016**, 55, 9030

Pd/a-$SrRuO_3$	77.29	4.0	5.17	1.0 M KOH + 1.0 M EtOH; 10 mV s^{-1}	*Nano Energy* **2020**, 67, 104247
Pd/Ni–SnO_2/C	33.8	3.1	9.17	1.0 M KOH + 1.0 M EtOH; 50 mV s^{-1}	*Catal. Sci. Technol.* **2020**, 10, 4099
Pd@5%NP/VC	78.12	0.80	1.02	0.5 M KOH + 0.5 M EtOH; 50 mV s^{-1}	*Int. J. Hydrogen Energy* **2020**, 45, 11116
$Pd_{85}Ni_{10}Bi_5$/C	50.0±1 3.9	2.68	0.53	1.0 M KOH + 1.0 M EtOH; 10 mV s^{-1}	*Electrocatalysis* **2020**, 11, 203
Pd/C–N,P,S	76.31	1.68	2.20	1.0 M NaOH + 1.0 M EtOH; 50 mV s^{-1}	*Catalysts* **2019**, 9, 114
$Pd_1Pt_{1.03}$/GA	-	3.41	-	1.0 M KOH + 1.0 M EtOH; 50 mV s^{-1}	*J. Power Sources* **2019**, 413, 98
PdAg sNWs	25.9	2.84	10.96	1.0 M KOH + 1.0 M EtOH; 50 mV s^{-1}	*Appl. Catal. B: Environ.* **2019**, 249, 116
PdP_2 NCs/rGO	105.1	1.6	1.52	0.5 M KOH + 0.5 M EtOH; 50 mV s^{-1}	*Appl. Catal. B: Environ.* **2019**, 242, 258
PdRh NBs	14.2	0.68	4.78	1.0 M KOH + 1.0 M EtOH; 50 mV s^{-1}	*Nanoscale* **2019**, 11, 2974
Pdcube-PANI HNSs	33.0	1.47	4.45	0.1 M KOH + 0.5 M EtOH; 50 mV s^{-1}	*J. Mater. Chem. A* **2019**, 7, 22029
Pd-Ag/G	92.1	5.2	5.64	1.0 M KOH + 1.0 M EtOH; 50 mV s^{-1}	*Ultrasonics sonochemistry* **2019**, 58, 104616
$Pd_{90}Au_{10}$/CNT	-	1.05	-	1.0 M KOH + 1.0 M EtOH; 50 mV s^{-1}	*Int. J. Hydrogen Energy* **2019**, 44, 11734
$Pd_7Ag_2Sn_2$/CNT	15.34	2.3	14.99	1.0 M KOH + 0.5 M EtOH; 50 mV s^{-1}	*Catal. Lett.* **2018**, 148, 1190
Pd/$_3$DNCNTs	20.1	0.77	3.83	1.0 M KOH + 1.0 M EtOH; 50 mV s^{-1}	*ACS Sustain. Chem. Engin.* **2018**, 6, 7918
Pt-$Bi(OH)_3$	50.6	6.87	13.57	1.0 M KOH + 0.5 M EtOH; 50 mV s^{-1}	*Nano Research* **2020**, 13, 265
Pt_1Co_1 nanowires	-	2.2	-	1.0 M KOH + 0.5 M EtOH; 50 mV s^{-1}	Ionics **2020**, 26, 3091
PdPtNi NSs	68.58	1.19	1.73	1.0 M KOH + 0.5 M EtOH; 50 mV s^{-1}	*J. Alloys Comp.* **2020**, 830, 154671
ultrasmall Pt NPs	30	4.9	16.33	0.5 M KOH + 0.5 M EtOH; 50 mV s^{-1}	*Int. J. Hydrogen Energy* **2020**, 45, 4341
Pt/Faujasite-C	102.6	0.84	0.82	0.5 M KOH + 0.5 M EtOH; 50 mV s^{-1}	*Int. J. Hydrogen Energy* **2019**, 44, 12365
Pt@HfSx/CNT	70	0.077	0.11	0.1 M KOH + 1.0 M EtOH; 50 mV s^{-1}	*J. Power Sources* **2019**, 410-411, 204
PtSn	66	0.67	1.01	0.2 M KOH + 0.2 M EtOH; 50 mV s^{-1}	*J. Colloid. Interface Sci.* **2019**, 545, 54
$Pt_{0.24}Cu_{0.76}$/C	82.2	0.616	0.75	0.1 M KOH + 1.0 M EtOH; 50 mV s^{-1}	*Int. J. Hydrogen Energy* **2019**, 44, 5970

$Pt_{1-x-y}Ir_xNi_y$	72.9	3.8	5.21	1.0 M KOH + 1.0 M EtOH; 50 mV s^{-1}	*Electrochem. Commun.* **2019**, 101, 61
Pt_2Bi	-	5.95	-	1.0 M NaOH + 1.0 M EtOH; 50 mV s^{-1}	*Nano Research* **2018**, 12, 429
$Pt/CoNiO_2$	66.4	1.136	1.71	1.0 M KOH + 0.5 M EtOH; 50 mV s^{-1}	*Sust. Energy Fuels* **2018**, 2, 229

Supplementary Table 6. Half-wave potential ($E_{1/2}$) and mass activity at 1600 rpm of benchmarking PGMs-based and non-PGMs catalysts for ORR in alkaline solutions ('-' represents unknown data, the mass activity of non-PGMs based materials was unavailable in the references).

Catalyst	$E_{1/2}$ (V vs. RHE)	Mass activity @ 0.9 V (A mg^{-1}) (A mg^{-1}_{PGMs})	Reference	Condition (room temperature, O_2 saturated electrolyte)
Pd/Co@N-C	0.880	0.94	This work	0.1 M KOH; 5 mV s^{-1}; 1600 rpm
Pd/C	0.790	0.12		
Pd/N-C	0.832	0.14		
Pd+Co@N-C	0.830	0.13		
PdMo metallene/C	0.95	16.37	*Nature* **2019**, 574, 81	0.1 M KOH; 20 mV s^{-1}; 1600 rpm
Pd metallene/C	0.89	0.65		
PdCuCo NCs/C	0.872	0.13	*Angew. Chem. Int. Ed.* **2016**, 55, 9030	0.1 M NaOH; 10 mV s^{-1}; 1600 rpm
60 wt.% Pd/GNS	0.85	0.84 @0.85V	*Electrochem. Commun.* **2011**, 13, 182	0.1 M NaOH; 10 mV s^{-1}; 1600 rpm
Au@Pd CSNTs	0.85	-	*Nano Research* **2014**, 7, 1205	1.0 M KOH; 5 mV s^{-1}; 1600 rpm
Amorphous Pd-P NPS	0.85	2.21 @0.85V	*J. Am. Chem. Soc.* **2014**, 136, 5217	0.1 M KOH; 10 mV s^{-1}; 1600 rpm
Pt-Pd	0.77	-	*Int. J. Hydrogen Energy* **2013**, 38, 12657	0.1 M KOH; 5 mV s^{-1}; 1600 rpm
PdAuCu *i*NPs	-	1.781	*Nano Energy* **2016**, 29, 268	1.0 M KOH; 5 mV s^{-1}; 900 rpm
30%Pd-Mn_2O_3	0.812	-	*J. Mater. Chem. A* **2014**, 2, 1272	0.1 M KOH; 10 mV s^{-1}; 1600 rpm
m-Pd_3Au/CNT	0.768	-	*Int. J. Hydrogen Energy* **2010**, 35, 9693	1.0 M KOH; 5 mV s^{-1}; 3600 rpm
palladium nanocubes/C	0.84	0.101	*Electrochem. Commun.* **2016**, 64, 9	0.1 M KOH; 10 mV s^{-1}; 1900 rpm
Ag@Pd/MW NTs	0.75	0.157	*Appl. Catal. B: Enviro.* **2013**, 138, 285	1.0 M KOH; 5 mV s^{-1}; 1600 rpm
Pd-HTRuSe(2:1)/C	0.785	-	*Fuel Cells* **2012**, 12, 963	0.1 M KOH; 3 mV s^{-1}; 1600 rpm
8.8 wt.% Pd@MnO_2/C	0.810	0.42	*J. Power Sources* **2011**, 196, 4491	0.1 M KOH; 10 mV s^{-1}; 2500 rpm

PdFe/C<br>PdMn/C	0.85<br>0.86	-	*J. Mater. Chem. A* **2016**, 4, 8337	0.1 M KOH; 5 mV s^{-1}; 1600 rpm
Core-shell Ni@Pd_3	~0.86	~0.04	*J. Mater. Chem. A* **2017**, 5, 9233	0.1 M KOH; 5 mV s^{-1}; 1600 rpm
Pd-B/C	0.883	0.170	*J. Phys. Chem. C* **2017**, 121, 3416	0.1 M KOH; 10 mV s^{-1}; 1600 rpm
Au@$Pd_{0.1}$	0.91	0.29	*ACS Catal.* **2018**, 8, 11287	0.1 M KOH; 5 mV s^{-1}; 1600 rpm
Pd nanoclusters/CNS	~0.82	0.286	*ChemElectroChem* **2017**, 4, 1349	0.1 M KOH; 10 mV s^{-1}; 1600 rpm
Pd@Ni–B/C (amorphous)	0.853	-	*RSC Adv.* **2014**, 4, 51126	1.0 M KOH; 10 mV s^{-1}; 900 rpm
Pd-HPW-CMK	0.84	-	*Adv. Energy Mater.***2015**, 5, 1401186	0.1 M KOH; 5 mV s^{-1}; 1600 rpm
Fe_2/Co_1-GNCL	0.846		*Angew. Chem. Int. Ed.* 2020,59, 16013	0.1 M KOH; 5 mV s^{-1}; 1600 rpm
Co/CNFs (900)	0.896 -		*Adv. Mater.* 2019, 31, 1808043	0.1 M KOH; 10 mV s^{-1}; 1600 rpm
Zn6Co_Fe	0.89 -		*J. Am. Chem. Soc.* 2019, 141, 10744	0.1 M NaOH; 10 mV s^{-1}; 1600 rpm
FeNIP/NCH	0.75 -		*J. Am. Chem. Soc.* 2019, 141, 7906	0.1 M KOH; 10 mV s^{-1}; 1600 rpm
Mn-Co	0.80 -		*Nat. Commun.* 2019, 10, 1506	1.0 M KOH; 5 mV s^{-1}; 1600 rpm
Zn-N-C-1	0.873 -		*Angew. Chem. Int. Ed.* 2019, 58, 7035	0.1 M KOH; 10 mV s^{-1}; 1600 rpm
N/Fe-CG	0.85 -		*Nano Energy* 2019, 56, 524	0.1 M KOH; 10 mV s^{-1}; 1600 rpm
G-Co*x*Fe1-*x* alloy	0.8 -		*Adv. Energy Mater.* 2019, 10, 1903215	0.1 M KOH; 5 mV s^{-1}; 1600 rpm
CoFe20@CC	0.86 -		*Adv. Mater.* 2019, 31, 1904689	0.1 M KOH; 5 mV s^{-1}; 1600 rpm
Fe-N-C HNSs	0.87 -		*Adv. Mater.* 2019, 31, 1806312	0.1 M KOH; 5 mV s^{-1}; 1600 rpm
NCo@CNT-NF700	0.861 -		*J. Am. Chem. Soc.* 2018, 140, 15393	0.1 M KOH; 10 mV s^{-1}; 1600 rpm
SA-Fe-HPC	0.81 -		*Angew. Chem. Int. Ed.* 2018, 57, 9038	0.1 M KOH; 5 mV s^{-1}; 1600 rpm
NiCo/NLG-270	0.82 -		*Adv. Mater.* 2018, 30, 1800005	0.1 M KOH; 5 mV s^{-1}; 1600 rpm
(Fe,Co)/CNT	0.954 -		*Energy Environ. Sci.* 2018, 11, 3375	0.1 M KOH; 10 mV s^{-1}; 1600 rpm
Fe-N_4 SAs/NPC	0.885 -		*Angew. Chem. Int. Ed* 2018, 57, 8614	1.0 M KOH; 10 mV s^{-1}; 1600 rpm
Fe-ISA/SNC	0.896 -		*Adv. Mater.* 2018, 30, 1800588	0.1 M KOH; 10 mV s^{-1}; 1600 rpm
FeCl1N4/CNS	0.921 -		*Energy Environ. Sci.* 2018, 11, 2348	0.1 M KOH; 10 mV s^{-1}; 1600 rpm
Fe-SAs/NPS-HC	0.912 -		*Nat. Commun.* 2018, 9, 5422	0.1 M KOH; 10 mV s^{-1}; 1600 rpm

Supplementary Table 7. Stability comparison of Pd/Co@N-C with other works in fuel cells.

**Samples**	**Test time**	**Current density retention**	**Decay rate**	**References**
Pd/Co@N-C as both anode and cathode	100 hours (0.1 mg_{Pd} cm^{-2})<br>1000 hours (1 mg_{Pd} cm^{-2})	87.2%<br>92.1%	0.167 mW cm^{-2} h^{-1}<br>0.035 mW cm^{-2} h^{-1}	This work
PtRu/C anode<br>Pt/C cathode	1000 hours	-	19.2 mW cm^{-2} h^{-1}	*Nat. Commun.* **2020**, *11*, 3561
P/C anode<br>Co-N-C cathode	27 hours	67%	-	*Angew. Chem. Int. Ed.* **2021**, *60*, 9516
Pt/C anode<br>20Mn-NC-second cathode	20 hours	78%	-	*Nat. Catal.* **2018**, *1*, 935
Pt/C anode<br>TPI@Z8(SiO2)-650-C cathode	20 hours	60%	-	*Nat. Catal.* **2019**, *2*, 259.
P/C anode<br>1.5Fe-ZIF cathode	100 hours	~30%	-	*Energy Environ. Sci.* **2019**, *12*, 2548
P/C anode<br>Fe_2N_6 cathode	20 hours	42%	-	*Matter* **2020**, *3*, 509
P/C anode<br>$Fe-N_4$/HOPC-c-1000 cathode	100 hours	53%	-	*Angew. Chem. Int. Ed.* **2020**, *59*, 2688.
P/C anode<br>Mn-N-C-HCl-800/1000 cathode	20 hours	80%	-	*ACS Catal.* **2020**, *10*, 10523
P/C anode<br>Co-N-C@F127 cathode	20 hours	45%	-	*Energy Environ. Sci.* **2019**, *12*, 250
P/C anode anode<br>HP-FeN_4 cathode	20 hours	66%	-	*Energy Environ. Sci* **2020**, *13*, 111-118
P/C anode<br>Co(mIm)-NC(1.0) cathode	100 hours	79.5%	-	*Nat. Catal.* **2020**, *3*, 1044
P/C anode<br>0.17CVD/Fe-N-C-kat cathode	100 hours	~55.6%	-	*Angew. Chem. Int. Ed.* **2020**, *59*, 21698

Supplementary Table 8. The maximum power density of benchmarking catalysts for DEFCs (the catalyst loading was calculated based on the loading of PGMs, '-' represents unknown data).

Anode catalyst	Cathode catalyst	Test condition	Power density		Reference
			**W mg^{-1}**	**mW cm^{-2}**	
Pd/Co@N-C<br>1.0 mg cm^{-2}	Pd/Co@N-C<br>1.0 mg cm^{-2}	60 ^{o}C; 1 M KOH + 2M EtOH<br>without backpressure	0.44	438	This work
Pd/N&F-C<br>0.3 mg cm^{-2}	Pd/N&F-C<br>0.3 mg cm^{-2}	60 ^{o}C; 1 M KOH + 2M EtOH<br>without backpressure	1.9	570	*Nature Energy* **2021**, *6*, 1144
60% Pt/C<br>0.5 mg cm^{-2}	60% Pt/C<br>0.5 mg cm^{-2}	23 ^{o}C; 3 M H2SO4 + 0.5 M V^{2+}<br>250 mL min^{-1} O_2	0.58	293	*Cell Reports Physical Science* **2020**, *1*, 100102
PdAg/CNT<br>1 mg cm^{-2}	ACTA 4020<br>3 mg cm^{-2}	60 ^{o}C; 6 M KOH + 3 M Glycerol<br>200 mL min^{-1} O_2	0.22	217	*Renewable Energy* **2017**, *105*, 647-655
SD-PtCoPt-CNT<br>0.5 mg cm^{-2}	Acta 4020<br>3 mg cm^{-2}	80 ^{o}C; 6 M KOH + 3 M Crude glycerol; 200 mL min^{-1} O_2	0.54	268.5	*Green Chemistry* **2013**, *15*, 1133-1137
Pd black<br>2 mg cm^{-2}	Pt black<br>2 mg cm^{-2}	60 ^{o}C; 2 M KOH + 1M EtOH<br>100 sccm O_2	0.072	143	*Fuel Cells* **2014**, *14*, 834
Pd@C/Ni<br>5 mg cm^{-2}	Acta 4020<br>2 mg cm^{-2}	60 ^{o}C; 5 M KOH + 3M EtOH<br>100 sccm O_2	0.04	202	*ACS Sustain. Chem. Eng.* **2019**, *7*, 11186
Pd_2Ru/C<br>1.04 mg cm^{-2}	MnO_2/carbon<br>4 mg cm^{-2}	90 ^{o}C; 1 M KOH + 2 M EtOH<br>100 mL min^{-1} O_2	0.13	140	*Appl. Catal. B: Environ.* **2018**, 224, 602
PdNi/C<br>1 mg cm^{-2}	60% Pt/C<br>3.9 mg cm^{-2}	60 ^{o}C; 5M NaOH + 3 M EtOH	0.36	360	*Int. J. Hydrogen Energy* **2011**, *36*, 9994
m-Pd_3Au<br>1 mg cm^{-2}	Nickel foam<br>2 mg cm^{-2}	60 ^{o}C; 5 M KOH + 3 M EtOH	0.185	185	*Int. J. Hydrogen Energy* **2010**, 35, 9693
Ni@Au@Pd/rGO<br>0.5 mg cm^{-2}	20 wt% Pt/C<br>1 mg cm^{-2}	80 ^{o}C; 2 M KOH + 1 M EtOH	0.3716	185.78	*Electrochimica Acta* **2018**, 271, 1
Pd-Ni_2P/C<br>0.8 cm^{-2}	Pt black<br>4 mg cm^{-2}	60 ^{o}C; 2 M KOH + 2 M EtOH	0.113	90	*ChemSusChem* **2014**, 7, 3374
Pd–(Ni–Zn)/C<br>2 mg cm^{-2}	Fe–Co cathodes<br>-	60 ^{o}C; 2 M KOH +<br>10 wt% EtOH	0.06	120	*Electrochem. Commun.* **2009**, 11, 1077
Pd NP/C	-	50 ^{o}C; 3M EtOH	-	12	*J. Power Sources* **2014**, 269, 370
Pt/C<br>1 mg cm^{-2}	Pd-Co-Mo/C<br>1 mg cm^{-2}	90 ^{o}C; 1 M EtOH	0.01	10	*Electrochimica Acta* **2010**, 55, 3002
Pd_2Ni_3/C<br>1 mg cm^{-2}	HYPERMEC^{TM}<br>-	60 ^{o}C; 3 M KOH + 5 M EtOH	0.09	90	*J. Power Sources* **2010**, 195, 1001
Pt_1Sn_1/C<br>1.3 mg cm^{-2}	Pt/C<br>1 mg cm^{-2}	90 ^{o}C; 1 M EtOH	0.04	52	*J. Power Sources* **2004**, 131, 217

Pd on Ni foam 2 mg cm^{-2}	Fe-Co YPERMEC™ K14	60 ºC; 5 M KOH + 3 M EtOH	0.051	102	*Int. J. Hydrogen Energy* **2011**, 36, 7707
Pd_3Ru/C 2 mg cm^{-2}	MnO_2 and active carbon	60 ºC; 3 M KOH + 3 M EtOH	0.062	123	*J. Power Sources* **2013**, 241, 696
Pd/C 1.5 mg cm^{-2}	Fe-Co YPERMEC™ K14	60 ºC; 1 M KOH + 3 M EtOH	0.037	56	*Int. J. Hydrogen Energy* **2012**, 37, 4413
$Pd_1Pt_{0.98}$/GA/NF	-	25 ºC; 5 M KOH + 3 M EtOH	-	3.6	*Solid State Sci.* **2018**, 75, 21
PdAu/C	-	40 ºC; 0.5 M NaOH + 1 M EtOH	-	40	*J. Power Sources* **2017**, 361, 276
45% PtRu/C 2 mg cm^{-2}	40% Pt/C 1 mg cm^{-2}	40 ºC; 2 M KOH + 2 M EtOH	0.0168	33.65	*Int. J. Hydrogen Energy* **2011**, 36, 5104
Pd-CeO_2/C 1 mg cm^{-2}	Fe-Co/C 2 mg cm^{-2}	25 ºC; 2 M KOH + 2 M EtOH	0.032	32	*ChemCatChem* **2015**, 7, 2214
20 wt%Pd/C 1 mg cm^{-2}	40 wt% Pt/C 2.0 mg cm^{-2}	60 ºC; 3 M KOH + 3 M EtOH	0.056	56.3	*Int. J. Hydrogen Energy* **2020**, 45, 19801
Pd_1N_{b1}/C 1 mg cm^{-2}	Pt/C 1.0 mg cm^{-2}	70 ºC; 1 M KOH + 2 M EtOH	0.027	27	*J. Electroanal. Chem.* **2020**, 858, 113824
Pd-Au aerogel 2 mg cm^{-2}	HypermecTM K14 4 mg cm^{-2}	40 ºC; 1 M KOH + 1 M EtOH	0.0156	31.2	*ACS Appl. Energy Mater.* **2020**, 3, 7527
Pd_1Sn_3/C 1 mg cm^{-2}	Pt/C 1 mg cm^{-2}	80 ºC; 1 M KOH + 2 M EtOH	0.042	42	*Renewable Energy* **2020**, 158, 49
Pt-Ru/CAB-H 1 mg cm^{-2}	Pt/CHSA 1 mg cm^{-2}	80 ºC; 2 M EtOH	0.0162	16.23	*Int. J. Hydrogen Energy* **2020**, 45, 574
Pd_1Nb_1/C 1 mg cm^{-2}	20 wt% Pt/C 1 mg cm^{-2}	50 ºC; 1 M KOH + 2 M EtOH	0.0181	18.11	*Int. J. Hydrogen Energy* **2018**, 43, 4505
Pt-Ru/C 2 mg cm^{-2}	Fe-N-C 2.5 mg cm^{-2}	80 ºC; 1 M KOH + 1 M EtOH	0.031	62	*Renewable Energy* **2018**, 115, 226
Pd_2Ru/C 3.48 mg cm^{-2}	MnO_2/C 3.48 mg cm^{-2}	70 ºC; 0.1 M KOH + 2 M EtOH	0.01322	46	*Appl. Catal. B: Environ.* **2018**, 224, 602
PdNi/EGO 1 mg cm^{-2}	40 wt% Pt/C 1 mg cm^{-2}	50 ºC; 1 M NaOH + 1 M EtOH	0.0166	16.6	*Appl. Catal. A: General* **2017**, 531, 29
20 wt% Pd/C 2.56 mg cm^{-2}	80 wt% (Bg-CA-M)-Fe/N/C 2.56 mg cm^{-2}	90 ºC; 1 M KOH + 2 M EtOH	0.025	64	*Carbon* **2017**, 125, 605
Pt-Ru/C 45 wt.% 1.33 mg cm^{-2}	Fe-N/C 2.5 mg cm^{-2}	80 ºC; 2 M KOH + 2 M EtOH	0.05474	72.8	*Appl. Catal. B: Environ.* **2017**, 205, 637
PtCu/C 1 mg cm^{-2}	Pt black 1 mg cm^{-2}	60 ºC; 1 M KOH + 3 M EtOH	0.0152	15.2	*Int. J. Hydrogen Energy* **2017**, 42, 27919

PtRu/C (40 wt.% Pt, 20 wt.% Ru)<br>1.5 mg cm^{-2}	$La_{0.7}Sr_{0.3}(Fe_{0.2}Co_{0.8})O_3$<br>45 mg cm^{-2}	60 ºC; 6 M KOH + 1 M EtOH	0.0184	27.6	*Electrochimica Acta* **2017**, 228, 325
PdSn/C<br>1 mg cm^{-2}	20 wt% Pt/C<br>1 mg cm^{-2}	100 ºC; 6 M KOH + 2 M EtOH	0.0272	27.2	*Int. J. Hydrogen Energy* **2016**, 41, 6457
$Pd_{12}Ru/C$<br>1.33 mg cm^{-2}	40 wt% Pt/C<br>1 mg cm^{-2}	90 ºC; 2 M KOH + 2 M EtOH	0.0729	97	*Int. J. Hydrogen Energy* **2016**, 41, 8954

Supplementary Note 1. The reaction process on different catalysts and the anode reaction, cathode reaction, and overall reaction in DEFCs

On the surfaces of Pd/C, Pd/N-C, and Pd+Co@N-C, the EOR follows the C2-4e pathway (where * is an active site; "C2" and "4" represent the number of carbon atoms in the final products and the number of electrons transferred per EtOH molecule, respectively):

$$* + OH^- \rightarrow *\text{-}OH_{ads} + e^- \quad (S1)$$

$$*\text{-}(CH_3CH_2OH)_{ads} + 3OH^- \rightarrow *\text{-}(CH_3CO)_{ads} + 3H_2O + 3e^- \quad (S2)$$

$$*\text{-}(CH_3CO)_{ads} + *\text{-}OH_{ads} \rightarrow CH_3COOH + 2* \text{ (RDS)} \quad (S3)$$

$$CH_3COOH + OH^- \rightarrow CH_3COO^- + H_2O \quad (S4)$$

$$\text{Total reaction: } CH_3CH_2OH + 5OH^- \rightarrow CH_3COO^- + 4H_2O + 4e^- \quad (S5)$$

While on the surface of the Pd/Co@N-C, the EOR follows the C1-12e pathway, where *is an active site; "C1" and "12" represent the number of carbon atoms in the final products and the number of electrons transferred per EtOH molecule, respectively :

$$*\text{-}(CH_3CH_2OH)_{ads} \rightarrow *\text{-}(CH_x)_{ads} + *\text{-}CO_{ads} + (6\text{-}x)H_{ads} \quad (S6)$$

$$*\text{-}(CH_x)_{ads} + (4+x)\, OH^- \rightarrow CO_2 + (2+x)\, H_2O + (4+x)\, e^- \quad (S7)$$

$$(6\text{-}x)\, H_{ads} + (6\text{-}x)\, OH^- \rightarrow (6\text{-}x)\, H_2O + (6\text{-}x)\, e^- \quad (S8)$$

$$*\text{-}CO_{ads} + 2\, OH^- \rightarrow CO_2 + H_2O + 2\, e^- \text{ (RDS)} \quad (S9)$$

$$\text{Total reaction: } CH_3CH_2OH + 12\, OH^- \rightarrow 2\, CO_2 + 9\, H_2O + 12\, e^- \quad (S10)$$

The anode, cathode, and overall reaction for alkaline direct EtOH fuel cells are shown in the following equations (standard conditions, E^0 represents the standard thermodynamic potential vs. standard hydrogen electrode, SHE):

$$\text{Anode: } C_2H_5OH + 12OH^- + 12e^- \rightarrow 2CO_2 + 9H_2O + 12e^-,\ E^0 = -0.74 \text{ V} \quad (S11)$$

$$\text{Cathode: } 1/2O_2 + H_2O + 2e^- \rightarrow 2OH^-,\ E^0 = 0.40 \text{ V} \quad (S12)$$

$$\text{Overall: } C_2H_5OH + 3O_2 \rightarrow 2CO_2 + 3H_2O,\ E^0 = 1.14 \text{ V} \quad (S13)$$

Supplementary Note 2. Comparison of ECSAs calculated from CO stripping method and Randles-Sevcik equation

The Randles-Sevcik equation is listed as follows:

$$I_p = (2.69 * 10^5) n^{3/2} A D^{1/2} C v^{1/2} \quad \text{(S14)}$$

where I_p is the peak current (A), n is the number of electrons participating in the reaction and here n = 1, A is the ECSA of the electrode (cm^2), D is the diffusion coefficient of $K_3[Fe(CN_6)]$ ($6.67*10^{-6}$ cm^{-2} s^{-1}), and C is the concentration of the molecule in the solution (mol L^{-1}). Based on the above Randles-Sevcik equation, the ECSA (A, cm^{-2}) is proportional to the value $I_p/v^{1/2}$, which is Randles's slope shown in Supplementary Fig. 26e-f, j. The ECSA is 0.8957, 0.9154, 2.1281, 1.0784, and 0.4530 cm^2 for Pd/C, Pd/N-C, Pd/Co@N-C, Pd+Co@N-C, and Co@N-C, respectively. Correspondingly, the ECSA normalized to the mass of Pd is 57.1, 58.3, 135.5, 68.7 m^2 g^{-1} (Supplementary Fig. 26k, right) for Pd/C, Pd/N-C, Pd/Co@N-C, Pd+Co@N-C, respectively. These values match well with the CO stripping results (Supplementary Fig. 26k, left). The ECSA value calculated from the Randles-Sevcik equation (135.5 m^2 g^{-1}) is a little higher than CO stripping method (117.9 m^2 g^{-1}), thus the electronic effect indeed affects the CO adsorption on the catalyst surface and causes an underestimation of the ECSA of the as-prepared catalyst. While considering the same trend when calculating the ECSA with CO stripping and the Randles-Sevcik equation of all catalysts, it will not affect any conclusion made in this work.

Supplementary Note 3. Determination of potential ranges of EOR Faradic efficiency in three-electrode half cell

We monitored the anode potential during the fuel cell test using an external reference electrode (Int. J. Hydrogen Energy 2012, 37, 2559-2570; J. Appl. Electrochem. 2013, 43, 1069-1078) to determine the range of the potential that should be applied in the three-electrode half-cell. As shown in Supplementary Fig. 34a, the cell voltage ranged from the open-circuit voltage (~1.06 V) to 0.21 V in a real fuel cell device, corresponding to the anode potentials range from 0 V to 0.70 V_{RHE} in a half-cell. Too high potentials (> 0.8 V_{RHE}) are useless in real DEFCs, while

too low potentials (<0.4 V_{RHE}) give a very low current density. Thus, four potentials, that is 0.4, 0.5, 0.6, and 0.7 V_{RHE} were selected to test the FE (Fig. 3f) for all four catalysts. The charge-to-product balance from EtOH to carbonate (Supplementary Fig. 34c) and acetate (Supplementary Fig. 34d) at 0.4 V_{RHE} on the four electrodes has been verified and used as representative potential. As shown in Supplementary Fig. 34b, on Pd/Co@N-C, the EOR at 0.4 V_{RHE} shows a high FE of EtOH to CO_2 (FE_{CO2} > 80%) and a low $FE_{acetate}$ (< 17%). In contrast, The FE_{CO2} at 0.4 V_{RHE} on Pd/C, Pd/N-C, and Pd+Co@N-C are 3.6%, 7.5%, and 18.4%, respectively. Correspondingly, all these three control samples have a high $FE_{acetate}$ of 95.4%, 85.3%, and 66.9%, respectively. These results indicate that the C-C bond was broken (complete 12e pathway) at low potential (0.4 V_{RHE}) on the Pd/Co@N-C. While the three other control samples just through a 4e incomplete pathway for EOR, no matter at low potential (0.4_{RHE}, Supplementary Fig. 34b) or high potentials (0.5 to 0.7 V_{RHE}, Fig. 3f).

Supplementary Note 4. Theoretical study of EOR

In our previous study (ACS Appl. Mater. Interfaces 2021, 13, 16602-16610), we have identified the CO oxidation to COOH as the rate-determining step for EOR to CO_2 on Pd(111) surface. Consequently, the enthalpy change for CO oxidation to the COOH step was used to describe the EOR activity and a low value of enthalpy change indicates a high activity of EOR. Considering the electron-deficient state of Pd NPs in the Pd/Co@N-C catalyst, we adopted the OH^- covered Pd(111) surface to model the Pd NPs in Pd/Co@N-C catalyst. The EOR activity on Pd(111) and OH-Pd(111) surface was determined by the enthalpy change for CO oxidation to COOH. The enthalpy change for CO oxidation to COOH step on OH-Pd(111) surface was predicted to be 1.28 eV, which is lower than that of 1.35 eV on the Pd(111) surface (Supplementary Fig. 36). This result indicates that the OH-Pd(111) surface shows a higher EOR activity than Pd(111) surface via accelerating the rate-determining step of EOR, thus explaining the enhanced EOR activity of Pd/Co@N-C catalyst (Fig. 3).

Supplementary Note 5. The EOR activity of Au film and the ATR-SEIRAS of Au film

To prove that the Au layer has a negligible impact on Pd/Co@N-C when testing the ATR-SEIRAS, we do the following two experiments.

First, we tested the EOR performance of Au film. As can be seen in Supplementary Fig. 40a, the Au film electrode has EOR activity in an alkaline solution. However, the peak potential for EOR on Au film was located at ~1.2 V_{RHE}. This potential is much higher than Pd/Co@N-C and other Pd-based reference catalysts (0.8-1.0 V_{RHE}), and thus beyond the potential range that we studied. Besides, the EOR performance was compared in Supplementary Fig. 40b, the Au film electrode shows one order of magnitude lower current density (0.62 A mg^{-1}_{Au}) than Pd/Co@N-C (7.06 A mg^{-1}_{Pd}). Based on the much lower EOR activity and much higher oxidation potential of Au film than Pd/Co@N-C, it is believed that the Au film has negligible impact on the reported results.

Second, we further performed the ATR-SEIRAS test on the Au/Si prism for EOR in an alkaline solution. As can be seen from Supplementary Fig. 40c-d, the spectra at different potentials (from 0 to 1.0 V_{RHE}) in 0.1 M KOH and 1 M EtOH show no obvious changes compared to that in 0.1 M KOH, indicating that almost no EOR activity on the Au/Si prism electrode before 1.0 V_{RHE}.

Based on the above, we believe that the peaks discussed are the information from Pd/Co@N-C catalyst, rather than from Au. The Au film/ disk electrode was widely used because it can provide a good signal-to-noise ratio (SNR), and thus the best spectroscopic result can be presented.

Supplementary Note 6. Confirmation of the C-C bond cleavage by isotopically labeled measurement.

The 1-^{13}C labeled EtOH ($CH_3{}^{13}CH_2OH$, >98%, purchased from Cambridge Isotope Laboratories, Inc.) was used to test the EOR. As can be seen from Supplementary Fig. 43a, the EOR CV curves tested in 1M KOH+ 1M $CH_3{}^{13}CH_2OH$ show almost the same performance as

that tested in 1M KOH+ 1M CH_3CH_2OH (Supplementary Fig. 28). Stable current density can be found for 12 hours stability test (Supplementary Fig. 43b), while at 1.1 V_{RHE}, a quickly decreased current density at the initial stage (before 2 hours) was found due to the surface passivation of Pd/Co@N-C at high potential. Supplementary Fig. 43c shows the isotopically labeled ^{13}C-NMR measurement. The fresh-prepared electrolyte (1.0 M KOH + 1.0 M $CH_3{}^{13}CH_2OH$) shows only two peaks at 18.5 and 59 ppm, which are attributed to the $-^{13}CH_2$ and $-CH_3$ in $CH_3{}^{13}CH_2OH$ (Biosens Bioelectron, 2020, 154, 112077; Electrochimica Acta, 2020, 331, 135254; Electrocatalysis, 2016, 8, 95-102; Chemical Communications, 2019, 55, 6042-6045). Besides these two peaks, two new peaks appear at 171 and 181 ppm after 12h stability tests appeared at different potentials, which can be attributed to the $^{13}CO_3^{2-}$ (due to the reaction of $^{13}CO_2$ and OH^-) and $CH_3{}^{13}COO^-$ as the complete and incomplete reaction of $CH_3{}^{13}CH_2OH$ oxidation reaction (Biosens Bioelectron, 2020, 154, 112077; Electrochimica Acta, 2020, 331, 135254; Electrocatalysis, 2016, 8, 95-102; Chemical communications, 2019, 55, 6042-6045). The peak intensity at 171 ppm is always much higher than at 181 ppm from 0.5 V to 1.1 V_{RHE}, indicating that the complete EOR is dominant on Pd/Co@N-C. The peak intensity reaches the maximum at 0.9 V_{RHE} and decreases at 1.1 V_{RHE}, which is consistent with the CV and i-t results. Thus, the isotopically labeled measurement further confirmed that (i) $^{13}CO_2$ ($^{13}CO_3^{2-}$ at 171 ppm) is the main EOR product; (ii) the peak intensity at 171 ppm is penitential-dependent, strongly demonstrates that the $^{13}CO_3^{2-}$ came from the complete oxidation of EtOH (1-^{13}C), rather than from contaminating of atmosphere or support corrosion.

Supplementary Note 7. Effect of carbonates produced from EOR on the membrane and catalyst

After 1000 hours of long-term stability test, the MEA was disassembled, and the reacted catalyst on the anode catalytic layer and anion-exchange membrane (AEM) were studied. The SEM images (Supplementary Fig. 53a-b) show that the AEM pores were negligibly blocked after 1000 hours test, suggesting the minimum negative effect of carbonates (CO_3^{2-}) on the membrane.

In addition, the specific area resistance and conductivity of the membrane just slightly increased and decreased respectively after 1000 hours test (Supplementary Fig. 53c), further demonstrating that the CO_3^{2-} has an inappreciable effect on the membrane. It seems that most CO_3^{2-} was exist in the electrolyte, as we found that the pH of the initial electrolyte was 13.9, while it decreased to 13.1 after 1000 hours of stability test, which can be due to the consumption of OH^- that reacted with the CO_2. The decreased pH and the potential membrane blocking can be effectively prevented by replacing electrolytes periodically, increasing the flow rate of electrolytes, or replacing a new membrane, which has been confirmed by our recent work (Nature Energy, 2021, 6, 1144-1153).

Besides, the effect of CO_3^{2-} on the catalyst was further evaluated. The CO stripping after 1000 hours of stability was tested to evaluate the ECSA and anti-CO poison ability of the reacted Pd/Co@N-C catalyst. the ECSA retention was 92.4% after 1000 hours test (Supplementary Fig. 53d, left axis). While the CO oxidation peaks just have a positive of ca. 30 mV after 1000 hours stability (0.82 V) test compared with the fresh sample (Supplementary Fig. 53d, right axis). These results indicate that the carbonates (CO_3^{2-}) may have a negative effect on reducing the active sites. However, due to the unique semi-embedded structure of Pd in the carbon layers, the catalytic activity for EOR (Supplementary Fig. 53f) was well kept, even after 1000 hours of stability, the activity retentions for EOR are still 89.1% and matches well with the ECSA results.

In all, the carbonates (CO_3^{2-}) that are produced from EOR just have negligible influence on the membrane and catalyst.

Supplementary Note 8. The movement of water (H_2O) and hydroxide radical (OH^-) in MEA and catalytic layer.

As can be seen from Supplementary Fig. 52, the thickness of the catalytic layer was about 74±2 μm. With such a thin layer, it has negligible mass and electron transfer resistance. Even though the KOH solution is not fed in the cathode, compared to the initial carbon paper (Supplementary Fig. 54a), a fully wetted cathode catalyst layer (with a pH > 11, inset in

Supplementary Fig. 54b) was found after 1000 hours long-term stability test, indicating that water can reach the entire cathode catalytic layer from the anode, as verified by the optical photograph shown in Supplementary Fig. 54b, this phenomenon matches well with recent review paper (Journal of Power Sources 2017, 341, 199-211) that water will transfer from anode to cathode (Supplementary Fig. 54c). Besides, the AEM has been fully swelled with water before operation since it was stored in ultrapure water before use, and 100% relative humidity of O_2 was fed to the cathode, which can ensure unrestrained movement of OH^- in the catalytic layer, and the fuel cells that using Nafion solution as binder can work smoothly.
